# Molecular pathology associated with altered synaptic transcriptome in the dorsolateral prefrontal cortex of depressed subjects

**DOI:** 10.1038/s41398-020-01159-9

**Published:** 2021-01-22

**Authors:** Yuta Yoshino, Bhaskar Roy, Nilesh Kumar, M. Shahid Mukhtar, Yogesh Dwivedi

**Affiliations:** 1 Department of Psychiatry and Behavioral Neurobiology, University of Alabama at Birmingham, Birmingham, AL 35294 USA; 2 Department of Biology, University of Alabama at Birmingham, Birmingham, AL 35294 USA

**Keywords:** Depression, Molecular neuroscience

## Abstract

Disrupted synaptic plasticity is the hallmark of major depressive disorder (MDD), with accompanying changes at the molecular and cellular levels. Often, the maladaptive molecular changes at the synapse are the result of global transcriptional reprogramming dictated by activity-dependent synaptic modulation. Thus far, no study has directly studied the transcriptome-wide expression changes locally at the synapse in MDD brain. Here, we have examined altered synaptic transcriptomics and their functional relevance in MDD with a focus on the dorsolateral prefrontal cortex (dlPFC). RNA was isolated from total fraction and purified synaptosomes of dlPFC from well-matched 15 non-psychiatric controls and 15 MDD subjects. Transcriptomic changes in synaptic and total fractions were detected by next-generation RNA-sequencing (NGS) and analyzed independently. The ratio of synaptic/total fraction was estimated to evaluate a shift in gene expression ratio in MDD subjects. Bioinformatics and network analyses were used to determine the biological relevance of transcriptomic changes in both total and synaptic fractions based on gene–gene network, gene ontology (GO), and pathway prediction algorithms. A total of 14,005 genes were detected in total fraction. A total of 104 genes were differentially regulated (73 upregulated and 31 downregulated) in MDD group based on 1.3-fold change threshold and *p* < 0.05 criteria. In synaptosomes, out of 13,236 detectable genes, 234 were upregulated and 60 were downregulated (>1.3-fold, *p* < 0.05). Several of these altered genes were validated independently by a quantitative polymerase chain reaction (qPCR). GO revealed an association with immune system processes and cell death. Moreover, a cluster of genes belonged to the nervous system development, and psychological disorders were discovered using gene–gene network analysis. The ratio of synaptic/total fraction showed a shift in expression of 119 genes in MDD subjects, which were primarily associated with neuroinflammation, interleukin signaling, and cell death. Our results suggest not only large-scale gene expression changes in synaptosomes, but also a shift in the expression of genes from total to synaptic fractions of dlPFC of MDD subjects with their potential role in immunomodulation and cell death. Our findings provide new insights into the understanding of transcriptomic regulation at the synapse and their possible role in MDD pathogenesis.

## Introduction

Major depressive disorder (MDD) is a common illness worldwide with a lifetime prevalence of 10.8%^1^. MDD is associated with increased morbidity and mortality and is a major risk factor for both suicidal ideation and attempt^2^. In fact, MDD patients frequently display affective dysregulated temperament and more than half of the MDD patients with such a temperament show higher levels of hopelessness and are at high suicide risk^3^. In addition, sensory perception has been implicated in the emotional processes of MDD patients and is often associated with clinical outcome. Any interventions should consider the unique sensory profiles of depressed individuals^4^. Interestingly, a high proportion of MDD patients show an inadequate response to antidepressants and ~30% of MDD patients do not respond to such treatment at all^5^. This disproportionate treatment response rate underscores the need to further understand key molecular and neurobiological risk factors in order to provide newer targets for effective drug development.

Synaptic plasticity is a central mechanism of neural adaption^6^. It has been shown that synaptic pathology due to reduced synaptic plasticity may play a significant role in the onset and development of MDD^7–9^. This is evident from various studies showing the loss of synaptic connections and impaired synaptogenesis in several brain areas of MDD subjects^7,10,11^. In the MDD brain, the deficits in synaptic plasticity often result from long-lasting maladaptive changes towards stress responses^12,13^. The lower number of synapses and reduced ability to re-pattern the synaptic connections are the hallmark of synaptic pathology. Many of these structural and functional deficits at the synapse are the direct outcome of abnormal gene expression regulation in their local environment^11,14^. Most often, the activity-dependent gene regulation at synapse results in repatterning of synaptic plasticity with a major impact on cognitive functions. Early onset of cognitive impairment has been shown to be associated with altered synaptic plasticity and enhanced GluA1 expression in the hippocampus of rats with depression-like behavior^15^. Similarly, the differential expression of postsynaptic NMDA (*N*-methyl-d-aspartate) and AMPA (α-amino-3-hydroxy-5-methyl-4-isoxasole-propionic acid) receptor subunits in the hippocampus and prefrontal cortex (PFC) has been reported in the rat model of depression^16^. Downregulation of genes encoding for the GABAergic and dopaminergic synapses as well as synaptic vesicle cycle and neuronal growth have been reported in the PFC of mice showing depression-like behavior^17^. Recently, using a single-cell transcriptomic approach, a study found the role of activity-dependent synaptic transmission in altering the synaptic efficacy in PFC of MDD subjects^18^. The study showed an overall enrichment of gene functions related to synaptic plasticity and potentiation in dlPFC of MDD subjects who died during their first episode^18^. In the rodent model, repeated stress causes loss of dendritic spines in the PFC and hippocampus^19,20^. Studies have also found morphometric changes in pyramidal neurons of PFC^21^ and hippocampus^22^ of MDD subjects. Microarray-based expression studies in dlPFC^11^ and hippocampus^23^ have also shown the loss of excitatory synapses with decreased expression of synapse-related genes in MDD subjects.

From the above studies, it is clearly evident that MDD is linked to altered synaptic plasticity; however, so far, there has been no significant report investigating large-scale transcriptomic changes at the synaptic level and associated molecular pathology in MDD. We hypothesize that MDD will be associated with large-scale changes as well as shifts in transcriptome at the synapse, which in turn will participate in the disease process by altering key physiological functions in the brain. In this study, we have comprehensively examined transcriptomic changes both in total and synaptosomal fractions of dlPFC. More specifically, a shift in gene expression ratios between the two fractions were used to elucidate the role of these specific genes at the functional level in MDD pathogenesis. Our findings suggest that in dlPFC of MDD brain, there were large-scale changes in the expression of genes both in total and synaptic fractions. Functionally, these genes were associated with response to a stimulus, immune system processes, and cell death. We also found that a significant number of genes showed a shift in their expression ratios from total to synaptic fractions and a majority of them were functionally related to cell death. Overall, our study provided insights into altered transcriptomic profiles both globally and at the synapse and their possible role in MDD pathogenesis.

## Methods

### Subjects

The study comes under exemption 4 and was approved by the Institutional Review Board of the University of Alabama at Birmingham. The study was performed in dlPFC (BA46) obtained from nonpsychiatric control subjects (referred to hereafter as controls) and MDD subjects. Postmortem brain samples were obtained from the Alabama Brain Collection cohort and the Maryland Brain Collection cohort. Demographic and clinical data are shown in Table S1. Demographic data include age, gender, postmortem interval (PMI), brain pH, race, cause of death, history of drug abuse, alcohol abuse, antidepressant medication at the time of death, and cause of death. Family members/informants signed written informed consent. Psychiatric diagnoses of the subjects were made by psychological autopsy using the Diagnostic and Statistical Manual of Mental Disorders, fourth edition (DSM-IV) criteria by means of Structured Clinical Interview for DSM-IV Axis I Disorders (SCID-I) interviews^24^. SCID-I is a structured diagnostic procedure to elicit diagnostic information employing proxy-based interviews complemented with medical and coroner records, followed by a consensus diagnosis reached by a panel of clinicians using the DSM-IV criteria. We used a total of 30 samples, although their power value was not estimated based on preobtained effect size. As can be seen in Table S1, there were eight males and seven females in the control group and seven males and eight females in the MDD group. All subjects were White Caucasians, except one subject who was African American in the MDD group. None of the subjects had died by suicide. In the MDD group, out of 15, 6 subjects showed positive antidepressant toxicology at the time of death. There were no significant differences in age (*p* = 0.87), PMI (*p* = 0.87), brain pH (*p* = 0.40) between control and MDD subjects.

The PFC samples were cut out of the coronal sections by a fine microdissecting (Graefe) knife under a stereomicroscope with low magnification. BA46 was taken just dorsal to the frontopolar area including the most polar portion of the superior and partly the middle frontal gyrus between the superior and intermediate frontal sulci. In the sections of the dissected cortical area, the gray and white matters were separated. The tissues were chopped into smaller pieces after flash-frozen in isopentane at −80 °C and later stored at −80 °C until use. All the experiments were performed in a blinded fashion.

### Synaptosome preparation

Synaptosomes were prepared by the methods reported earlier^25^ with slight modifications. About 100 mg tissue samples were homogenized manually by Fisherbrand™ RNase-Free disposable pellet pestles (Fisher Scientific, USA) in homogenizing buffer (HB) buffer (50 mM HEPES, pH 7.5, 125 mM NaCl, 100 mM sucrose, 10 mM EDTA, pH 8.0, 2 mM phenylmethylsulfonyl fluoride (PMSF), 1× Halt protease inhibitor cocktail, 160 U/ml RNaseOUT). Lysates were partially collected as a total fraction. After centrifugation at 20,000 × *g* for 20 min at 4 °C, the supernatant was collected (S fraction). The pellet was resuspended with HB and used in sucrose gradient centrifugation to obtain synaptosome fraction with 0.32, 0.85, 1.0, and 1.2 M sucrose in 1 mM NaHCO_3_ and 160 U/ml RNaseOUT. A synaptic fraction was recovered in 1.0–1.2 M interface after centrifugation at 200,000 × *g* for 2 h at 4 °C. Purified synaptic fractions were stored at −80 °C.

### Western blot-based synaptosome validation

Protein lysates were prepared by methanol/chloroform precipitation and dissolved in RIPA buffer [Tris-HCl (pH 8.0) 50 mM, NaCl 150 mM, NP-40 1%, sodium deoxycholate 0.5%, sodium dodecyl sulfate (SDS) 0.1%, EDTA 2.5 mM supplemented with protease inhibitors, 1 mM PMSF, and 25 μm of MG-132]. Lysates containing 20 µg were subject to immunoblot analysis after resolving electrophoretically on denatured discontinuous SDS-polyacrylamide gel. The following primary antibodies were used for validation with optimal concentrations [proliferating cell nuclear antigen (PCNA; 1:2000, Cell Signaling Technology #2586), postsynaptic density (PSD-95; 1:1000, Cell Signaling Technology #2507), and synapsin I (1:3000, Cell Signaling Technology #5297)]. Horseradish peroxidase-conjugated secondary antibody (Applied Biological Materials Inc., Canada) was used separately for each primary antibody.

### RNA isolation

TRIzol® (Invitrogen, USA) was used to isolate RNA from both purified synaptosomes and total tissue homogenates following the method described earlier^26^. RNA purity was checked by NanoDrop (260/280 nm; cutoff ≥1.8) and their integrity by agarose gel electrophoresis (Fig. S1A).

### Construction and sequencing of RNA-sequencing (RNA-seq) Ilumina library for total fraction and synaptosomal RNAs

Approximately 1–2 µg total RNA of each sample was used for RNA-seq library preparation. Briefly, messenger RNA (mRNA) was isolated from total RNA with NEBNext® Poly(A) mRNA magnetic isolation module (New England Biolabs, USA). Alternatively, ribosomal RNA (rRNA) was removed from total RNA with a RiboZero magnetic gold kit (Epicenter, USA). The enriched mRNA or rRNA depleted RNA was used for RNA-seq library preparation using KAPA stranded RNA-seq library prep kit (Illumina, USA). The library preparation procedure included: (1) fragmentation of RNA molecules; (2) reverse transcription (RT) to synthesis first-strand complementary DNA (cDNA); (3) second-strand cDNA synthesis incorporating dUTP; (4) end-repair and A-tailing of the double-stranded cDNA; (5) Illumina compatible adapter ligation; and (6) PCR amplification and purification. The completed libraries were qualified on Agilent 2100 Bioanalyzer for concentration, fragment size distribution (400–600 bp), and adapter dimer contamination. The amount was determined by absolute quantification qPCR (quantitative polymerase chain reaction) method. The barcoded libraries were mixed in equal amounts and used for sequencing. The DNA fragments in well-mixed libraries were denatured with 0.1 M NaOH to generate single-stranded DNA molecules, loaded onto channels of the flow cell at 8 pM concentration, and amplified in situ using TruSeq SR Cluster Kit v3-cBot-HS (Illumina, USA). Sequencing was carried out using Illumina HiSeq4000, according to the manufacturer’s instructions. Sequencing was carried out by running 150 cycles.

### Bio-computational analysis of RNA-seq data

Raw data files in FASTQ format were generated from the Illumina sequencer. To examine the sequencing quality, the quality score plot of each sample was plotted. Sequence quality was examined using the FastQC software. After quality control, the fragments were 5′, 3′-adaptor trimmed and filtered ≤20 bp reads with the cutadapt software. The trimmed reads were aligned to a reference genome with the Hisat 2 software. Based on alignment statistical analysis (mapping ratio, rRNA/mitochondrial RNA content, fragment sequence bias), it was determined whether the results could be used for subsequent data analysis. The expression levels [FPKM (Fragments Per Kilobase of transcript per Million mapped reads) value] of known genes and transcripts were calculated using ballgown through the transcript abundances estimated with StringTie. The number of identified genes per group was calculated based on the mean of FPKM in group ≥0.5. Heatmap and *k*-means clustering were performed to visualize the expressed genes using iDEP tool suit^27^. Differentially expressed gene analysis was performed with R package ballgown. Expressed genes were used to create Volcano plots using R (v.3.6.3) library. The threshold for *p* value cutoff of the expressed gene was assigned ≤0.05. Up- and downregulated genes are depicted by red and green colors dots, respectively. The remaining insignificant genes are depicted as dark-gray dots. The *x*- and *y*-axis correspond to log 2 fold change (FC) value and the mean expression value of log 10 (*p* value), respectively.

### Determination of the relative expression ratio (synaptic vs. total fractions)

The ratio of synaptic/total fraction of each gene was calculated in control and MDD subjects separately using normalized FPKM values. Next, these values were used for comparison of the ratio between control and MDD subjects. Step plot was created using python matplotlib package depicting the ratio of respective classes.

### cDNA synthesis and qPCR

About 500 ng RNA was used to synthesize cDNA using M-MLV Reverse Transcriptase (Invitrogen, USA) and oligo (dT) primer as previously described (29361849). We randomly selected 16 significantly altered genes (eight each from total and synaptic fractions) from RNA-seq data for further qPCR validation. The relative transcript expression was measured with 1× EvaGreen qPCR master mix (Applied Biological Material Inc., Canada), and 0.8 μM each of gene-specific forward and reverse primers (Table S2). Twenty-fold diluted cDNA was used as a template to conduct qPCR with the following protocol: initial denaturation at 95 °C for 10 min, repeating 40 cycles of denaturation at 95 °C for 10 s, primer annealing at 60 °C for 15 s, and an elongation at 72 °C for 20 s. To exclude the possibility of primer dimer formation and secondary product amplification, EvaGreen specific dissociation curve analysis was performed following an initial denaturation at 72 °C for 1 min, annealing at 55 °C for 30 s, and repeat denaturation step at 95 °C for 30 s. Glyceraldehyde 3-phosphate dehydrogenase (*GAPDH*), β-actin (*ACTB*), and *18s rRNA* were used as housekeeping genes and their geometric means were used as the reference value. FC was calculated following Livak’s ΔΔCt method^28^.

### Functional annotation and the pathway analysis

GO annotations of biological process (BP), molecular function (MF), and cellular component (CC) were performed by the Protein ANalysis THrough Evolutionary Relationships (PANTHER) v14.1 (http://www.pantherdb.org/). We also picked up PANTHER Pathways, an original pathway analyzed by PANTHER with *p* values < 0.05 and false discovery rate < 0.05.

### Core analysis and construction of gene–gene network

Ingenuity Pathway Analysis software (IPA; Qiagen, USA) was utilized for generating a module for the functional enrichment of target genes to decipher their role in the canonical pathways, molecular networks, and disease pathways using Fisher’s exact test. *P* value threshold was set at ⩽0.05. Gene–gene network was also constructed by IPA software with the following criteria: data sources, all; confidence level, experimentally observed; species, all; tissues and cell lines, all; relationship types, all; publication date range, January 1974–December 2020; node types, all; biofluids, all; disease, neurological disease and psychological disorders. Briefly, the individual networks were ranked by the score in the *p* value calculation of IPA assay, which calculated the probability of the queried genes that were part of a network found randomly therein. For this purpose, the right-tailed Fisher’s exact test was performed, and scores were a negative exponent of the *p* values. If the number of query genes in the network was higher, it would reflect a high score or low *p* value. Finally, all the individual networks were merged into a gene–gene network, and nodes were clustered or grouped according to the IPA network analysis result, i.e., diseases and function modules. Further, spot plot was created using exported enrichment data from IPA. The size of the spot was relative to the normalized and negatively transformed enrichment *p* values. Cytoscape platform (3.8.0)^29^ was used to visualize the networks.

### Statistical analysis

Statistical analyses were conducted using the SPSS software (v.25; IBM, USA). Shapiro–Wilk test was used to assess the normality of the data. The average differences of age, PMI, and brain pH were assessed by the Student’s *t* test. Differences in gender, drug abuse, alcohol abuse, and history of antidepressant medication were analyzed by Fisher’s exact test. The average differences in gene expressions and ratios were compared by the Student’s *t* test. The correlation between the FC of RNA-seq and qPCR was calculated by using the Pearson’s correlation coefficient. Correlations of gene expressions with covariates were also conducted with the Pearson’s correlation coefficient. Statistical significance was set at the 95% level (*p* = 0.05).

## Results

### Synaptosome isolation and validation

To understand the synaptosome-associated transcription, we isolated synaptosome by sucrose gradient centrifugation method. Subsequently, we validated the purity of synaptosomes by performing a Western blot analysis. As shown in Fig. S1B, synapsin I was present in all three fractions (total, S, and synaptic fraction); PSD-95 was highly enriched in synaptic fraction and absent in the S fraction; and PCNA, a nuclear marker, was absent in the synaptic fraction, but present in total and S fractions. The expression (FC) of PCNA (FC: 1.06, *p* = 0.53), PSD-95 (FC: 0.92, *p* = 0.49), and synapsin I (FC: 1.23, *p* = 0.12) were not significantly different between MDD and control subjects. Our results are consistent with a previous synaptosome preparation study from our laboratory using human brain^25^.

### Global gene expression analysis in the total fraction

**Fig. 1:**
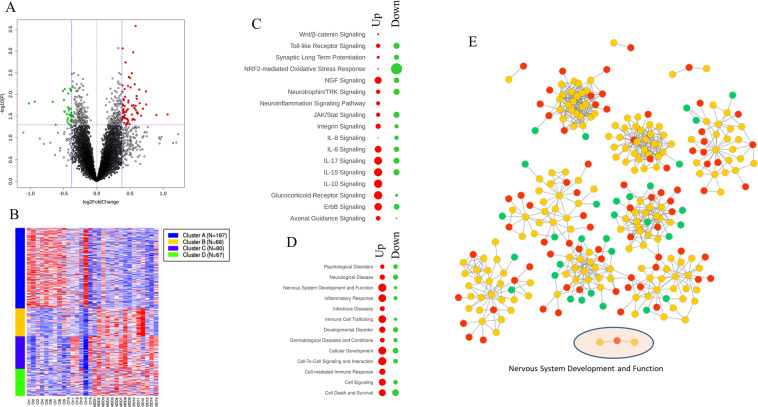
Volcano plot and heatmap based on total fraction RNA-seq data. **A** Volcano plot was created by all differentially expressed genes (14,005 genes). *Y*-axis shows the mean expression value of log 10 (*p* value), and the *x*-axis displays the log 2-fold change value. Blue vertical line means 1.3-fold change [log 2 (0.584)]. The purple horizontal line means *p* = 0.05 [−log 10 (1.30)]. **B** *k*-Means clustering was done over heatmap of only significant (*p* < 0.05) genes made over standard deviation normalization (212 upregulated and 199 downregulated genes). Canonical pathway, disease and function, and gene–gene networks were analyzed using 73 upregulated and 31 downregulated genes (>1.3-fold). The significant results of the canonical pathway (**C**) and disease and function (**D**) are shown in red (up) and green (down) circles. Spots/circles size is the function of −log(base = 10) of Fisher’s exact test enrichment *p* value. **E** IPA gene–gene network yielded 13 disease function modules. All 13 subnetwork were merged to build one network. Red nodes represent upregulated genes, whereas green nodes represent downregulated genes. Other genes are presented in orange color. Ct control, MDD major depressive disorder.

A total of 14,005 genes were found to be expressed in the total fraction. A Volcano plot was created with all the differentially expressed genes (Fig. 1a). A total of 212 (1.51%) and 199 (1.42%) genes were significantly (*p* < 0.05) up- and downregulated, respectively. Highly altered genes (top and bottom 20) are listed in Table 1, while a list of all significantly altered genes is provided in Table S3. Additionally, applying a *k*-Means clustering algorithm grouped those differentially expressed genes into four distinct clusters (Fig. 1b).

**Table 1:** Significantly altered genes (top and bottom 20 genes) in MDD subjects compared to controls in the total fraction.

Gene symbol	Ensemble ID	Locus	Fold change	*p* Value	*q* Value
Top 20 upregulated genes					
*TMEM189-UBE2V1*	ENSG00000124208.16_3	Chr20:48,697,661–48,770,174	2.093301568	0.028970933	0.998154148
*HNRNPUL2-BSCL2*	ENSG00000234857.2_3	Chr11:62,457,747–62,494,856	1.862368887	0.029806405	0.998154148
*SPI1*	ENSG00000066336.11_2	Chr11:47,376,411–47,400,127	1.674116789	0.016973309	0.998154148
*RASD1*	ENSG00000108551.4_2	Chr17:17,397,751–17,399,709	1.671422929	0.008487038	0.998154148
*HIST1H1D*	ENSG00000124575.6_2	Chr6:26,234,496–26,235,161	1.6182264	0.021265941	0.998154148
*HSPA2*	ENSG00000126803.9_3	Chr14:65,002,623–65,012,891	1.587199534	0.03068192	0.998154148
*OLFML3*	ENSG00000116774.11_2	Chr1:114,522,013–114,578,194	1.566558294	0.015948973	0.998154148
*H1FX*	ENSG00000184897.5_2	Chr3:129,033,614–129,035,120	1.557723521	0.007713264	0.998154148
*TMEM119*	ENSG00000183160.8_4	Chr12:108,983,622–108,992,096	1.533970067	0.027726499	0.998154148
*SYN2*	ENSG00000157152.16_3	Chr3:12,045,876–12,232,900	1.529728606	0.025668161	0.998154148
*SLC2A5*	ENSG00000142583.17_3	Chr1:9,095,166–9,148,537	1.524777663	0.0259714	0.998154148
*MGP*	ENSG00000111341.9_2	Chr12:15,034,115–15,038,860	1.520322977	0.026291557	0.998154148
*CD248*	ENSG00000174807.3_2	Chr11:66,081,958–66,084,515	1.517306136	0.012513065	0.998154148
*SYN1*	ENSG00000008056.13_3	ChrX:47,431,297–47,479,342	1.514899142	0.010820886	0.998154148
*ADAMTS2*	ENSG00000087116.14_3	Chr5:178,537,852–178,772,431	1.504012857	0.023024431	0.998154148
*SELENON*	ENSG00000162430.16_3	Chr1:26,126,667–26,144,715	1.502459486	0.018145924	0.998154148
*EHD1*	ENSG00000110047.17_2	Chr11:64,619,114–64,655,768	1.499851368	0.00026562	0.998154148
*ELK1*	ENSG00000126767.17_2	ChrX:47,494,920–47,510,003	1.485837489	0.005823038	0.998154148
*OLFML2B*	ENSG00000162745.10_2	Chr1:161,952,982–161,993,644	1.484633409	0.038133823	0.998154148
*IGFBP4*	ENSG00000141753.6_2	Chr17:38,599,713–38,613,983	1.484402089	0.020842833	0.998154148
Bottom 20 downregulated genes					
*OTOGL*	ENSG00000165899.10_2	Chr12:80,603,233–80,772,870	0.758383143	0.0073387	0.998154148
*FP15737*	ENSG00000215298.3	Chr8:23,430,157–23,432,974	0.751653359	0.029617859	0.998154148
*ACAD11*	ENSG00000240303.7_3	Chr3:132,276,982–132,379,567	0.750470175	0.048358226	0.998154148
*RBBP4*	ENSG00000162521.18_3	Chr1:33,116,743–33,151,812	0.749822389	0.020815195	0.998154148
*HSPD1*	ENSG00000144381.16_3	Chr2:198,351,305–198,381,461	0.745771365	0.023242035	0.998154148
*TOGARAM1*	ENSG00000198718.12_3	Chr14:45,431,411–45,543,634	0.743039018	0.009521289	0.998154148
*GSTM5*	ENSG00000134201.10_3	Chr1:110,254,877–110,318,050	0.740939805	0.035152072	0.998154148
*FSBP*	ENSG00000265817.2_3	Chr1:95,384,398–95,449,180	0.739829097	0.019761943	0.998154148
*PPARA*	ENSG00000186951.16_3	Chr22:46,546,424-46,639,653	0.736169585	0.044314345	0.998154148
*QSER1*	ENSG00000060749.14_2	Chr11:32,914,724–33,014,862	0.735464221	0.038886837	0.998154148
*FABP6*	ENSG00000170231.15_2	Chr5:159,614,374–159,665,742	0.731863888	0.028418238	0.998154148
*ZNF709*	ENSG00000242852.6_3	Chr19:12,571,998–12,624,668	0.71556927	0.007476814	0.998154148
*RALB*	ENSG00000144118.13_3	Chr2:120,997,640–121,052,289	0.714954298	0.025116048	0.998154148
*DUSP6*	ENSG00000139318.7_2	Chr12:89,741,009–89,747,048	0.713571153	0.024744029	0.998154148
*MDM4*	ENSG00000198625.12_2	Chr1:204,485,507–204,527,248	0.70941623	0.013522487	0.998154148
*FCF1*	ENSG00000119616.11_2	Chr14:75,179,847–75,205,323	0.702405537	0.008291242	0.998154148
*NME1-NME2*	ENSG00000011052.21_3	Chr17:49,230,951–49,249,105	0.651236153	0.049573833	0.998154148
*MKLN1*	ENSG00000128585.17_3	Chr7:130,794,855–131,181,395	0.630768248	0.014691013	0.998154148
*AL662899.3*	ENSG00000263020.6_4	Chr6:31,633,879–31,641,323	0.522821095	0.014578824	0.998154148
*AD000671.1*	ENSG00000188223.9_4	Chr19:36,236,579–36,245,420	0.491543271	0.015996805	0.998154148

### Core analysis and gene–gene network construction with significantly up- and downregulated genes in the total fraction

Genes that showed highly altered expression levels (73 upregulated and 31 downregulated genes) were used for functional prediction analysis using the IPA software. In the canonical pathway analysis, several immune-related pathways emerged, based on altered genes both in up- and downregulated groups (Fig. 1c). These included neuroinflammation and interleukin pathways, as well as glucocorticoid signaling. In addition, synaptic functions such as long-term potentiation, axon guidance, and neurotrophin signaling were also found on the list. Under disease and function modules, several important pathways, including inflammatory response, cell-mediated immune response, neurological pathway, psychological pathway, and nervous system development, showed significant enrichment for both up- and downregulated gene categories (Fig. 1d). To get insights into the potential disease or specific gene function, the target genes were subsequently mapped over the networks available in the Ingenuity database. Specifically, a gene–gene interaction map was created using up- and downregulated gene sets from the nervous system development and function to demonstrate their connectivity in a functional network (Fig. 1e). In the map, upregulated genes are represented with red nodes, whereas green nodes are representative of downregulated genes. Functional analysis revealed that diverse clusters in this gene–gene map were enriched in psychological disorders, neurological disease, and nervous system development and function categories.

### Global gene expression analysis in the synaptic fraction

**Fig. 2:**
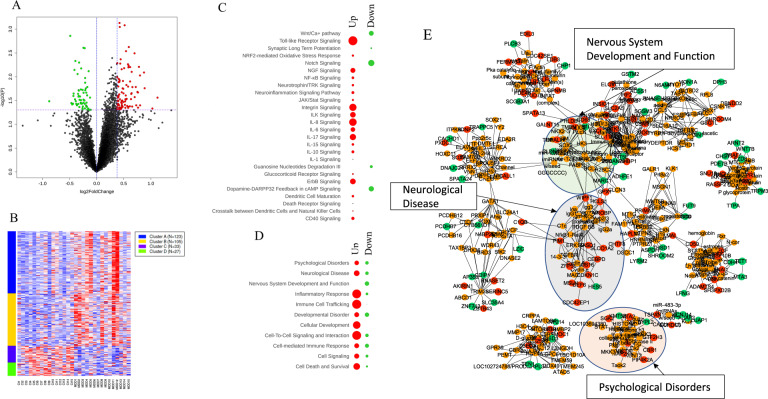
Volcano plot and heatmap based on synaptic fraction RNA-seq data. **A** The Volcano plot was created by all differentially expressed genes (13,236 genes). *Y*-axis shows the mean expression value of log 10 (*p* value), and the *x*-axis displays the log 2 fold change value. The blue vertical line means 1.3-fold change line [log 2 (0.584)]. The purple horizontal line means *p* = 0.05 [−log 10 (1.30)]. **B** *k*-Means clustering was done over heatmap of only significant (*p* < 0.05) genes made over standard deviation normalization (234 upregulated and 60 downregulated genes). Canonical pathway, disease and function, and gene–gene network were analyzed using 93 upregulated genes (1.3-fold) and all downregulated genes (60 genes). Significant results from canonical pathway (**C**) and disease and function (**D**) are shown in red (up) and green (down) circles. Spots/circles size is a function of −log(base = 10) of Fisher’s exact test enrichment *p* value. **E** IPA gene–gene network yielded 12 diseases function modules. All 12 subnetwork were merged to build one network. Red nodes represent upregulated genes, whereas green nodes represent downregulated genes. Other genes are presented in orange color. Ct control, MDD major depressive disorder.

A total of 13,236 genes were expressed in the synaptic fraction. The Volcano plot was created with all the differentially expressed genes Fig. 2a. An MDD-specific up- and down-regulation was found in 234 (1.77%) and 60 (0.45%) genes, respectively. The highly altered genes (top and bottom 20) are presented in Table 2, and all significantly altered genes are listed in Table S4, respectively. Based on significantly changed genes (234 upregulated and 60 downregulated), a heatmap was constructed with *k*-means clustering, which showed four distinct gene clusters (Fig. 2b).

**Table 2:** Significantly altered genes in MDD subjects compared to control subjects in synaptosome (top and bottom 20).

Gene symbol	Ensemble ID	Locus	Fold change	*p* Value	*q* Value
Top 20 upregulated genes					
*ZFP36*	ENSG00000128016.5_2	Chr19:39,897,453–399,00,052	2.1776576	0.0275288	0.999912394
*C1QB*	ENSG00000173369.15_2	Chr1:22,979,255–22,988,031	2.0394579	0.0465707	0.999912394
*CHTF8*	ENSG00000168802.12_3	Chr16:69,151,913–69,166,487	1.8470062	0.0174307	0.999912394
*RASD1*	ENSG00000108551.4_2	Chr17:17,397,751–17,399,709	1.8322651	0.0081353	0.999912394
*CEBPD*	ENSG00000221869.4_2	Chr8:48,649,471–48,651,648	1.7427126	0.0399716	0.999912394
*TMEM88B*	ENSG00000205116.3_2	Chr1:1,361,508–1,363,167	1.7405742	0.0093953	0.999912394
*KIF19*	ENSG00000196169.14_2	Chr17:72,322,349–72,351,959	1.7176642	0.0061926	0.999912394
*MOG*	ENSG00000204655.11_3	Chr6:29,624,758–29,640,149	1.6982876	0.0446928	0.999912394
*S100A9*	ENSG00000163220.10_2	Chr1:153,330,330–153,333,503	1.6963496	0.0214211	0.999912394
*RGS16*	ENSG00000143333.6_2	Chr1:182,567,758–182,573,543	1.6860759	0.020914	0.999912394
*HAPLN2*	ENSG00000132702.12_2	Chr1:156,589,086–156,595,517	1.660522	0.0399065	0.999912394
*CX3CL1*	ENSG00000006210.6_2	Chr16:57,406,370–57,418,960	1.6509677	0.0164296	0.999912394
*GALNT15*	ENSG00000131386.17_2	Chr3:16,216,156–16,273,499	1.6278187	0.0268205	0.999912394
*SLC2A5*	ENSG00000142583.17_3	Chr1:9,095,166–9,148,537	1.6249166	0.003212	0.999912394
*ADAMTS4*	ENSG00000158859.9_2	Chr1:161,154,098–161,168,846	1.6133472	0.0502441	0.999912394
*ADNP2*	ENSG00000101544.8_2	Chr18:77,866,915–77,905,406	1.5810882	0.0164974	0.999912394
*CD68*	ENSG00000129226.13_3	Chr17:7,482,785–7,485,431	1.5771296	0.0423986	0.999912394
*FPR1*	ENSG00000171051.8_3	Chr19:52,248,425–52,307,363	1.5668887	0.007887	0.999912394
*CDC42EP1*	ENSG00000128283.6_4	Chr22:37,956,454–37,965,412	1.5649011	0.0222008	0.999912394
*TYROBP*	ENSG00000011600.11_2	Chr19:36,395,303–36,399,197	1.5585077	0.022949	0.999912394
Top 20 downregulated genes					
*CDH4*	ENSG00000179242.15_4	Chr20:59,827,482–60,515,673	0.7962247	0.019576	0.999912394
*CLDN10*	ENSG00000134873.9_2	Chr13:96,085,858–96,232,013	0.7947775	0.0254516	0.999912394
*N6AMT1*	ENSG00000156239.11_2	Chr21:30,244,513–30,257,693	0.794196	0.0192057	0.999912394
*FUT9*	ENSG00000172461.10_2	Chr6:96,463,860–96,663,488	0.7924129	0.0261338	0.999912394
*FZD8*	ENSG00000177283.7_3	Chr10:35,927,177–35,931,206	0.7911683	0.0373176	0.999912394
*MTA3*	ENSG00000057935.13_2	Chr2:42,721,709–42,984,087	0.7883872	0.0229091	0.999912394
*CISD3*	ENSG00000277972.1_2	Chr17:36,886,488–36,891,297	0.7833351	0.0087768	0.999912394
*WNT7B*	ENSG00000188064.9_3	Chr22:46,316,242–46,373,009	0.7770486	0.0398209	0.999912394
*MRPL34*	ENSG00000130312.6_3	Chr19:17,403,418–17,417,652	0.7698633	0.0371592	0.999912394
*ASPDH*	ENSG00000204653.9_3	Chr19:51,014,857–51,017,947	0.7658521	0.0327412	0.999912394
*TTPA*	ENSG00000137561.4_2	Chr8:63,961,112–63,998,612	0.7616707	0.0046591	0.999912394
*LFNG*	ENSG00000106003.12_2	Chr7:2,552,163–2,568,811	0.7578215	0.0298689	0.999912394
*KCNJ14*	ENSG00000182324.6_3	Chr19:48,958,766–48,970,237	0.7523269	0.0071809	0.999912394
*CHST7*	ENSG00000147119.3_2	ChrX:46,433,219–46,457,843	0.7443651	0.0226256	0.999912394
*CYB561D1*	ENSG00000174151.14_4	Chr1:110,036,674–110,045,554	0.7427098	0.0254082	0.999912394
*TEN1*	ENSG00000257949.6_3	Chr17:73,975,301–73,996,667	0.7386488	0.0370837	0.999912394
*CHP1*	ENSG00000187446.11_2	Chr15:41,523,037–41,574,088	0.7310931	0.0382482	0.999912394
*ARNT2*	ENSG00000172379.20_3	Chr15:80,696,692–80,890,278	0.7235799	0.0358096	0.999912394
*ACSS1*	ENSG00000154930.14_2	Chr20:24,986,866–25,039,616	0.7132899	0.0013848	0.999912394
*HES5*	ENSG00000197921.5_2	Chr1:2,460,184–2,461,684	0.5447051	0.0328057	0.999912394

### Core analysis and gene–gene network construction with significantly up- and downregulated genes in the synaptic fraction

IPA-based core functional analysis was done using 93 upregulated genes (>1.3-fold) and all downregulated genes (60 genes). In the result of the canonical pathway, several immune-related pathways such as nuclear factor-κB (NF-κB), Janus kinase/signal transducers and activators of transcription (JAK/STAT) pathways, NF-E2 p45-related factor 2 (Nrf2)-mediated oxidative stress response, and interleukin-1 (IL-1), IL-6, IL-10, and Toll-like receptor (TLR) signaling were observed in the upregulated group. CD40 signaling component from adaptive immunoresponse also discovered (Fig. 2c). In terms of disease and function, inflammatory response, immune cell trafficking, cell death, and survival appeared prominently in the upregulated gene group compared to downregulated ones. In addition, nervous system development and function, neurological disease, and psychological disorders were significantly associated with differentially regulated genes (Fig. 2d). Using the functional connectivity of each gene that belongs to these three clusters, gene–gene networks were created (Fig. 2e). In this network, 14 disease function modules were used as a subnetwork. Finally, all 14 subnetworks were merged together to build one cohesive network. Red nodes are upregulated genes, whereas green nodes are downregulated genes. Other nodes are represented in orange.

### Functional annotation

Next, we followed a gene set enrichment analysis to determine the shared biological functions of differentially regulated genes based on significant GO terms. The GO analysis was conducted based on upregulated 93 genes (>1.3-fold) in the synaptic fraction. In all, 150 GO terms in BP, 5 GO terms in MF, and 2 GO terms in CC were found. All of them were statistically significant (Table S5). In BP, several GO terms were found associated with response to a stimulus, immune system process, and cell death. These three terms were further divided into clusters by using the program associated with GO Tree View of Gene Ontology Browser (http://www.informatics.jax.org/vocab/gene_ontology/). As can be seen in Table S6, a large number of genes were significantly associated with innate immune response, regulation of inflammatory response, glial cell activation, immune response-regulating signaling pathway, and immune response-activating signal transduction. In terms of MF GO terms, several protein binding GO terms were found (Table S5). Of them, TLR binding (GO:0035325) had the highest fold enrichment score. In CC GO terms, podosome (GO:0002102) and secretory granule membrane (GO:0030667) showed statistical significance.

### Validation of gene expression data with qPCR in the total and synaptic fractions

Based on significant expression dysregulation, we randomly selected eight genes (five upregulated: *RASD1*, *H1FX*, *SYN1*, *IGFBP4*, *SYN2*; three downregulated: *HPSD1*, *RBBP4*, *ZNF32*) from the total fraction and eight genes (four upregulated: *TLR2*, *ZFP36*, *IRF1*, *ELK1*; four downregulated: *HES5*, *ASPDH*, *CYB561D1*, *FUT9*) from synaptic fraction-based RNA-seq data. The geometric means (*GAPDH*, *ACTB*, and *18s rRNA*) were not significantly different between control and MDD groups in both total (*t* = 1.324, d.f. = 28, *p* = 0.196) and synaptic (*t* = 0.906, d.f. = 28, *p* = 0.373) fractions. The mRNA expression level of *RBBP4* (*p* = 0.027) was significantly changed in MDD subjects, as shown in Fig. S2A. The mRNA expression of *H1FX* (*p* = 0.061), *SYN1* (*p* = 0.335), *IGFBP4* (*p* = 0.125), *SYN2* (*p* = 0.899), *HPSD1* (*p* = 0.430), and *ZNF32* (*p* = 0.438) had similar trends with those of RNA-seq, but they did not reach to the significance levels. mRNA expression levels of *TLR2* (*p* = 0.023), *ZFP36* (*p* = 0.031), *ELK1* (*p* = 0.004), *HES5* (*p* = 0.014), and *ASPDH* (*p* = 0.015) were significantly changed in MDD subjects as shown in Fig. S2B. The mRNA expression of *IRF1* (*p* = 0.07), *CYB561D1* (*p* = 0.09), and *FUT9* (*p* = 0.08) had similar trends with those of RNA-seq data, but marginally missed significance levels. The fold change (MDD/control) of qPCR result was significantly correlated with that of RNA-seq data in the total (*r* = 0.827; *p* = 0.011; Fig. S2C) and synaptosomes (*r* = 0.973; *p* < 0.001; Fig. S2D) fractions.

The mRNA expression of genes in both total and synaptic fractions was evaluated for their association with covariates, such as age, gender, brain pH, PMI, antidepressant exposure, and alcohol abuse. None of the changes was associated with covariates except for *RBBP4* (*p* = 0.001) and *ZFN32* (0.04) in total fraction and *CYB561D1* (*p* = 0.05) in synaptic fraction (Tables S7 and S8).

### Shift in gene expression ratios between synaptic vs. total fractions in MDD subjects and associated functional annotations

Gene enrichment ratio was performed for all 13,178 genes between total and synaptic fractions. The individual relative ratio of genes had a wide range in the control group (Fig. S3). A majority of ratios were <1.0 (median ratio = 0.89, >1.0: *n* = 2902, <1.0: *n* = 10,276). In addition, 0.43% of ratio showed enrichment >1.5-fold (0.24% >2-fold) and 8.64% showed depletion >1.5-fold (1.76% >2-fold). The highest ratio was found in the ratio of *EPDR* gene, which was 8.9-fold enriched in the synaptic fraction than the total fraction. On the other hand, the lowest ratio was found in *C7orf55-LUC7L2* (7.28-fold less abundant in the synaptic fraction). The top and bottom 20 genes displaying high and low gene enrichment ratios are shown in Table S9. When control and MDD subjects were compared, a large number of genes (119 genes, *p* < 0.05) showed a significant shift in their ratios in MDD subjects (Table 3). These genes were further subjected to core analysis for canonical pathway using IPA. The results demonstrated six different immune-related pathways, including neuroinflammation, IL signaling (IL-6, IL-8, IL-17, and IL-15), TLR signaling, and NF-κB signaling. Several synaptic terms (e.g., long-term potentiation, axon guidance, neurotrophic signaling) and cell death (death receptor signaling) were also uncovered from this analysis (Fig. S4).

**Table 3:** Shift in gene expression ratios between synaptic vs. total fractions in MDD subjects.

Gene symbol	Ensembl ID	Control (Ct) ratio	MDD ratio	MDD/Ct	*p* Value
*AC010531.1*	ENSG00000131152.4_4	1.109117	0.451195	0.406806	0.020
*AC068987.1*	ENSG00000260415.3	0.617041	1.035928	1.678864	0.025
*AC092835.1*	ENSG00000233757.6_3	0.740429	0.890826	1.203121	0.040
*ACAD11*	ENSG00000240303.7_3	0.442945	0.759169	1.713913	0.043
*ACTL6A*	ENSG00000136518.16_3	0.588869	0.83387	1.416053	0.035
*ANKRD20A2*	ENSG00000183148.6_3	0.328245	0.533641	1.625738	0.044
*AP000350.4*	ENSG00000251357.4_4	0.794562	0.645044	0.811824	0.017
*APOL4*	ENSG00000100336.17_3	0.76277	1.415282	1.855449	0.033
*ARHGAP24*	ENSG00000138639.17_2	0.856571	1.10586	1.291032	0.028
*ATP10B*	ENSG00000118322.12_2	0.668256	0.949887	1.421441	0.037
*BBS4*	ENSG00000140463.13_3	0.727548	0.934154	1.283976	0.027
*BCL7B*	ENSG00000106635.7_2	1.074546	1.000904	0.931467	0.042
*C14orf119*	ENSG00000179933.5_2	0.73052	0.86649	1.186128	0.024
*CACNB3*	ENSG00000167535.7_2	1.092732	0.961579	0.879977	0.029
*CAPN3*	ENSG00000092529.23_3	0.618857	0.8293	1.340051	0.042
*CDIP1*	ENSG00000089486.16_3	1.043427	0.963377	0.923281	0.032
*CDK5R2*	ENSG00000171450.5_2	0.981479	0.925658	0.943125	0.034
*CREM*	ENSG00000095794.19_4	0.76757	0.877173	1.142792	0.048
*CRISPLD1*	ENSG00000121005.8_2	0.546605	0.845734	1.547248	0.041
*CEP85*	ENSG00000130695.14_2	0.770408	0.951118	1.234563	0.038
*CNDP2*	ENSG00000133313.14_3	1.032092	0.902963	0.874886	0.036
*CWC25*	ENSG00000273559.4_2	0.732397	0.952897	1.301066	0.047
*CX3CL1*	ENSG00000006210.6_2	0.649123	0.903662	1.392127	0.013
*CYP46A1*	ENSG00000036530.8_2	0.988763	0.906198	0.916496	0.038
*DAXX*	ENSG00000204209.11_4	1.068359	0.967248	0.905358	0.021
*DDX3Y*	ENSG00000067048.16_3	0.78034	0.833528	1.06816	0.044
*DIABLO*	ENSG00000184047.16_3	0.835929	0.982002	1.174744	0.043
*DNAH9*	ENSG00000007174.17_3	0.661659	0.889836	1.344856	0.049
*DPH2*	ENSG00000132768.13_2	1.084157	0.956665	0.882405	0.016
*EFCAB11*	ENSG00000140025.15_3	0.785504	1.073849	1.367084	0.039
*EHD1*	ENSG00000110047.17_2	1.109755	0.898865	0.809967	0.006
*ENDOU*	ENSG00000111405.8_2	1.002033	1.600795	1.597547	0.044
*FAM212A*	ENSG00000185614.4_2	0.936095	0.711428	0.759995	0.025
*FAM219A*	ENSG00000164970.14_2	1.143265	1.012943	0.886009	0.034
*FAM78A*	ENSG00000126882.12_2	1.158076	1.008606	0.870932	0.040
*FBXL18*	ENSG00000155034.18_3	1.340458	1.095543	0.81729	0.024
*FCF1*	ENSG00000119616.11_2	0.75	1.20057	1.600761	0.033
*FGD1*	ENSG00000102302.7_2	1.08939	1.007333	0.924676	0.046
*GADD45A*	ENSG00000116717.11_2	0.898648	1.091988	1.215146	0.045
*GALK2*	ENSG00000156958.14_3	0.713817	0.875241	1.226142	0.035
*GALNT4*	ENSG00000257594.3_3	0.565474	0.923666	1.633437	0.032
*GDI1*	ENSG00000203879.11_2	0.985422	0.90179	0.915131	0.044
*GPIHBP1*	ENSG00000277494.1_2	0.564343	0.769699	1.363885	0.048
*GSG1L*	ENSG00000169181.12_4	0.885812	1.14154	1.288693	0.003
*GUCA1B*	ENSG00000112599.8_2	0.862628	1.521691	1.764017	0.009
*HAUS7*	ENSG00000213397.10_4	0.615618	0.86666	1.407789	0.047
*HSPA2*	ENSG00000126803.9_3	1.29328	0.91394	0.706684	0.041
*IGSF21*	ENSG00000117154.11_3	0.998621	0.940739	0.942038	0.031
*IMMT*	ENSG00000132305.20_2	0.811733	0.939611	1.157537	0.027
*INPP1*	ENSG00000151689.12_2	0.702529	0.885719	1.260758	0.018
*JMJD4*	ENSG00000081692.12_2	0.911505	1.218259	1.336535	0.004
*KIAA0040*	ENSG00000235750.9_2	0.381431	0.789758	2.070513	0.023
*KLHL20*	ENSG00000076321.10_3	0.810558	1.045121	1.289384	0.015
*KRBA2*	ENSG00000184619.3_3	0.856094	1.106994	1.293075	0.001
*LACTB2*	ENSG00000147592.8_2	0.672986	0.93313	1.386551	0.029
*LIMK1*	ENSG00000106683.14_2	1.229904	1.050647	0.854251	0.019
*LLGL2*	ENSG00000073350.13_2	0.529035	0.768594	1.452823	0.029
*LRP2*	ENSG00000081479.12_2	0.440017	0.777273	1.766463	0.037
*LSM1*	ENSG00000175324.9_2	0.948025	1.037139	1.093999	0.047
*MAP2K2*	ENSG00000126934.13_2	1.043799	0.978676	0.937609	0.039
*MOG*	ENSG00000204655.11_3	0.737132	0.937363	1.271635	0.027
*MSH6*	ENSG00000116062.14_3	0.749917	0.972315	1.296563	0.041
*MTA3*	ENSG00000057935.13_2	1.279879	1.067502	0.834065	0.015
*MTO1*	ENSG00000135297.15_2	0.723714	0.916889	1.266921	0.020
*NDE1*	ENSG00000072864.14_3	0.630604	0.935422	1.483376	0.007
*ODC1*	ENSG00000115758.12_3	0.948832	1.050529	1.10718	0.021
*OSMR*	ENSG00000145623.12_3	0.492132	0.750504	1.525004	0.015
*PARP12*	ENSG00000059378.12_2	0.524304	0.786383	1.499861	0.022
*PCCB*	ENSG00000114054.13_3	0.870068	0.946768	1.088155	0.036
*PHLDA2*	ENSG00000181649.5_2	1.002686	0.690281	0.688432	0.041
*PLSCR1*	ENSG00000188313.12_3	0.593825	0.791404	1.332722	0.025
*PM20D2*	ENSG00000146281.5_2	0.834077	0.986752	1.183046	0.043
*POPDC3*	ENSG00000132429.9_2	0.716706	0.938912	1.310038	0.011
*PPARA*	ENSG00000186951.16_3	0.907818	1.409824	1.552982	0.048
*PPIE*	ENSG00000084072.16_3	0.722037	0.916081	1.268745	0.010
*PPP1R18*	ENSG00000146112.11_2	1.126685	0.970509	0.861385	0.037
*PSENEN*	ENSG00000205155.7_3	0.79529	0.877975	1.103969	0.040
*PTP4A3*	ENSG00000184489.11_4	0.96628	0.871331	0.901738	0.044
*RBBP4*	ENSG00000162521.18_3	0.852028	1.003293	1.177535	0.046
*RFT1*	ENSG00000163933.9_3	0.881272	1.058105	1.200657	0.025
*RGS16*	ENSG00000143333.6_2	0.747131	1.081041	1.446923	0.022
*RHOQ*	ENSG00000119729.11_3	1.32783	1.141333	0.859548	0.043
*RILPL2*	ENSG00000150977.10_2	1.145368	1.009566	0.881434	0.027
*RIPK1*	ENSG00000137275.13_2	0.795016	0.952675	1.19831	0.016
*RNASE4*	ENSG00000258818.3_3	0.496841	0.945895	1.903816	0.042
*RNF214*	ENSG00000167257.10_3	1.022524	0.873818	0.85457	0.004
*RPL6*	ENSG00000089009.15_2	0.945796	1.022728	1.081341	0.027
*S100PBP*	ENSG00000116497.17_3	0.597426	0.997503	1.669666	0.032
*SCO2*	ENSG00000130489.14_3	0.898872	0.774805	0.861975	0.020
*SEC13*	ENSG00000157020.17_3	0.799129	0.926857	1.159834	0.046
*SEMA6A*	ENSG00000092421.16_2	0.733411	0.921617	1.256617	0.036
*SH3BP4*	ENSG00000130147.15_3	0.782312	0.975496	1.246939	0.015
*SHC4*	ENSG00000185634.11_2	0.705852	1.152284	1.632473	0.042
*SLC39A7*	ENSG00000112473.17_3	0.843186	0.94314	1.118543	0.045
*SMKR1*	ENSG00000240204.2_2	0.938199	1.101026	1.173553	0.034
*SMNDC1*	ENSG00000119953.12_2	0.673909	0.853418	1.266369	0.033
*SPAG1*	ENSG00000104450.12_2	0.696272	0.947067	1.360198	0.034
*STUM*	ENSG00000203685.9_3	1.103296	1.36013	1.232788	0.023
*SUSD3*	ENSG00000157303.10_3	0.944125	0.582786	0.617277	0.017
*SYPL2*	ENSG00000143028.8_2	0.253054	0.164774	0.651142	0.009
*TAF6*	ENSG00000106290.14_3	1.070497	0.988082	0.923012	0.022
*TAF6L*	ENSG00000162227.7_3	0.925703	0.798461	0.862545	0.046
*TBC1D3*	ENSG00000197681.8	0.528187	1.078091	2.041115	0.046
*TMEM138*	ENSG00000149483.11_2	0.545755	0.801324	1.468284	0.010
*TMEM230*	ENSG00000089063.14_2	0.870686	0.938659	1.078069	0.038
*TMEM250*	ENSG00000238227.7_4	1.29612	1.078574	0.832156	0.018
*TNFSF13*	ENSG00000161955.16_3	0.991214	1.133431	1.143478	0.049
*TRAPPC5*	ENSG00000181029.8_3	1.232167	0.971181	0.788189	0.037
*TRIM16*	ENSG00000221926.11_4	0.72783	1.020225	1.401735	0.046
*TULP3*	ENSG00000078246.16_3	0.823316	1.013812	1.231377	0.045
*TVP23C-CDRT4*	ENSG00000259024.6_3	0.645766	1.116121	1.728368	0.028
*TXNIP*	ENSG00000265972.5_2	0.798444	0.891237	1.116217	0.049
*U2AF1L5*	ENSG00000275895.6_4	0.661736	0.927801	1.40207	0.007
*ZC3H12A*	ENSG00000163874.10_3	0.46926	0.950929	2.026445	0.018
*ZNF229*	ENSG00000278318.4_3	0.903331	1.162093	1.286453	0.035
*ZNF334*	ENSG00000198185.11_3	0.537001	0.790072	1.471268	0.047
*ZNF610*	ENSG00000167554.14_2	0.647825	0.887647	1.370194	0.019
*ZNF823*	ENSG00000197933.12_3	0.694311	0.896145	1.290696	0.047
*ZNF84*	ENSG00000198040.10_3	0.566025	1.072708	1.89516	0.040

## Discussion

### Key findings

To our knowledge, this is the first large-scale transcriptomic study in humans that not only examined the overall gene expression changes in dlPFC of MDD subjects, but also paid close attention to genes that were expressed at the synapse. The study also focused on whether gene pools realigned at the synapse. We found large-scale changes in the transcriptome at the synapse of dlPFC from MDD subjects. Interestingly, there was a shift in gene expression ratio when the synaptic fraction was compared with the total fraction. GO, derived from these genes, revealed an association with immune system processes and cell death. In addition, gene–gene network analysis showed a cluster of genes that belonged to nervous system development and psychological disorders. On the other hand, genes, that showed a shift in the synaptic fraction, were primarily associated with neuroinflammation, IL signaling, and cell death.

We chose dlPFC because of its critical role in MDD pathogenesis^30^. dlPFC receives input from specific sensory cortices and is densely interconnected with premotor areas and involved in executive and cognitive functions, such as intention formation, goal-directed action, and attentional control^31^. Functional imaging studies have shown that at resting state, dlPFC is hypoactive in MDD patients^32^, but demonstrates greater task-related activation associated with working memory and cognition^33,34^. Interestingly, dlPFC control of cognitive function is associated with the regulation of negative emotion, a feature found to be dysregulated in MDD subjects^35,36^. dlPFC hypoactivity in MDD is reversed by antidepressant treatment^37,38^. In addition, PFC areas, particularly, dlPFC is involved in activating the hypothalamic-pituitary-adrenal axis (HPA) in response to stress as well as in negative feedback regulation^39,40^. The role of HPA axis abnormalities is well documented in MDD pathogenesis^41,42^.

Our transcriptomic profiling using RNA-seq reliably detected 14,005 genes in the total fraction isolated from dlPFC. Of them, 411 genes were differentially regulated in MDD subjects with a similar number in up- or downregulated groups (212 upregulated and 199 downregulated). RNA-seq data from synaptic fraction showed that the gene pool at the synapse was as abundant as the total fraction where 13,236 genes were detected. Interestingly, contrary to the total fraction, the number of genes that were upregulated in MDD subjects were much higher than the downregulated ones (234 upregulated and 60 downregulated), suggesting a contrasting pattern of gene regulation at the synapse. Our qPCR experiment not only validated expression changes in genes in both total and synaptic fractions, but also showed their significant correlation with RNA-seq data.

### Functional analysis

#### Immune response

Functional annotation and pathway analysis associated with RNA-seq data in MDD subjects for both total and synaptic fractions were quite revealing. It was observed that most of the genes that were upregulated in the total fraction were associated with immune functions or were involved in pathways that regulate the immune response. The pathways that were linked with immune system included TLRs, ILs, and NF-κB. Glucocorticoid receptor (GR) signaling also appeared on the list. Disease and function analyses indicated that dysregulated genes were closely associated with nervous system development and function, as well as psychological disorders along with developmental disorders. Gene–gene network interaction analysis further reiterated the notion that closely associated genes formed clusters and were associated with these disorders and functions. Surprisingly, synaptic genes that were found to be dysregulated in MDD subjects were also profoundly inclined towards the regulation of the immune system. For example, canonical pathways that were enriched for significantly altered synaptic genes were associated with IL (*IL-1*, *IL-6*, *IL-8*, *IL-10*, *IL-15*, and *IL-17*) and TLR signaling. Thus, the dysregulated genes in both total and synaptic fractions of MDD subjects were linked to immune response. The finding is not surprising given that poor immune functions and alterations in pathways that regulate them are the prominent features of MDD pathogenesis^43^, and defective inflammatory pathways are associated with poor treatment response to antidepressants in treatment-resistant depressed patients^44^. It has been shown that MDD is accompanied by systemic immune activation or an inflammatory response, which involves phagocytic cells, T cell activation, and an increased production of ILs^45,46^. Several human postmortem brain studies, including our own, have shown alterations in the transcript expression of several ILs and TLRs, as well as TNF-α^47–50^ in frontal cortical areas of MDD and suicide subjects. In fact, we have shown that proinflammatory *TNF-α* transcript is modulated differently in dlPFC of MDD subjects due to a defect in the binding of specific noncoding RNA to its 3′-untranslated region^47^.

Several other signaling pathways that are critical in immune regulation were also part of the functional response mediated by altered synaptic genes. These include CD40, NF-κB, and JAK/STAT pathways. CD40, a member of the TNF superfamily, is a receptor molecule expressed on the cell surface of activated T cells, B cells, and dendritic cells^51–53^. It is an important contributor to inflammatory processes in the central nervous system (CNS)^54^. On the other hand, NF-κB and JAK/STAT signaling pathways are involved in the development of the classical pathway of inflammation^55,56^. NF-κB is a critical mediator of stress-impaired neurogenesis and depressive behavior^57,58^, whereas JAK/STAT signaling pathway is activated by stress via acid sphingomyelinase^59^ and is involved in *N*-acetylcysteine-mediated antidepressant-like effects^60^.

#### Nrf2-mediated oxidative stress response

One of the pathways that was significantly related to altered synaptic genes was Nrf2-mediated oxidative stress response. Nrf2 is a transcription factor that regulates the expression of genes that protect cells from various injuries via their anti-inflammatory effects. Nrf2 prevents lipopolysaccharide-induced transcriptional upregulation of proinflammatory cytokines IL-6 and IL-1β^61^. Interestingly, the expression of *Nrf2* has been found to be lower in PFC of mice that show depression-like as well as learned-helpless behavior^62,63^. *Nrf2* knockout (KO) mice themselves exhibit depression-like phenotype and produce higher levels of proinflammatory cytokines^64^. Moreover, *Nrf2* KO mice show lower expression of brain-derived neurotrophic factor (BDNF) in frontal cortical and limbic brain areas. Agonist to TrkB, a receptor for BDNF, shows antidepressant effect in *Nrf2* KO mice. In addition, pretreatment with sulforaphane, a Nrf2 activator, prevents depression-like phenotype in mice after inflammation, or chronic social defeat stress. Lower expression of *Nrf2* has also been noted in the parietal cortex of MDD subjects. Nrf2 and NF-κB pathways cross-talk to each other such that in response to inflammatory stimuli, Nrf2 signaling inhibits the overproduction of proinflammatory cytokines and limits the activation of NF-κB, which is generally involved in the transcription of proinflammatory mediators such as IL-6, TNF-α, and IL-1^61^. Our finding that upregulated synaptic genes were related to pathways associated with Nrf2-mediated oxidative stress response is quite interesting. It is possible that alterations in *Nrf2* and mediated regulatory genes may be directly involved in MDD pathogenies via the production of proinflammatory cytokines.

#### GR signaling

GR signaling was also part of the compromised functional response associated with altered genes in MDD subjects. GR contributes to HPA hyperactivity reported in MDD^65,66^. Alterations in GR, which could be due to its reduced expression, nuclear translocation, binding affinity to ligands, or binding of glucocorticoid response elements to DNA, can lead to glucocorticoid resistance, which in turn, can induce susceptibility to exaggerated inflammatory responses, and consequently, depression^67^. Our earlier report shows that the expressions of GR and GR-inducible target gene *GILZ* were significantly altered in dlPFC of suicide subjects^68^. Interestingly, it has been proposed that excessive stress can alter the relationship between the innate immune and CNS activities^69^. Whether our finding of compromised GR signaling is directly related to pathways that regulate immune functions is not clear at present; however, it has been reported that excessive psychological stress can activate TLRs, NF-κB, and the inflammasome NLRP3, which in turn can induce the secretion of IL-1β, IL-6 and factors associated with innate immune response, causing symptoms characteristic of depressive illness such as dysphoria and anhedonia^70^. IL-6 and TNF-α are also involved in the induction of indoleamine 2,3-dioxygenase, which depletes tryptophan and induces depression^71,72^. Recently, we reported that the pharmacological manipulation of cellular stress pathways can induce depression phenotype in rats, which involved TNF-α, NF-κB, and TLRs^73^. Our findings of GR signaling along with immune response mediated through differentially regulated synaptic genes suggest that they may, either independently or in concert, be playing a critical role in compromised immune functions and may be responsible for the development of depressive behavior.

#### Death receptor signaling

The analysis of transcriptomic data also showed several cell death-related GO terms, including apoptotic process and cell death and survival. Altered synaptic genes were also associated with death receptor signaling. So far, there is no known report of the apoptotic pathology itself in the PFC of MDD subjects. However, previous gene expression studies in PFC of human^74^ and mouse^75^ have predicted the possible involvement of apoptotic pathways in depression. Further, transcription factor Kruppel-like factor 11 and transcriptional repressor protein, R1, which influence cell growth and survival, are significantly altered in the brain of rats exposed to chronic social defeat^76^ or in the PFC of MDD subjects^77^. Moreover, both preclinical and human postmortem brain studies show a loss of neuronal function in response to persistent stress leading to neuronal atrophy, which could be related to programed cell death^78^. It has also been shown that neurotrophic factor-activated signaling pathways are involved in the upregulation of antiapoptotic proteins Bcl-2, Bcl-xl, and Bax. Previous studies have shown that antidepressants produce their effects by inducing the expression of neurotrophins while targeting apoptotic proteins, that helped d in reversing the apoptosis-induced structural alterations in limbic areas^79–81^. Our present study is consistent with these findings and suggests that apoptotic signaling may be a part of the functional response associated with altered synaptic genes in MDD subjects.

#### Shift in gene expression at synapse

Ratios of gene transcripts between synaptic and total fractions showed that many genes either had low or high synaptic enrichment in the control group; however, a significant shift was noted in the expression of genes in the MDD group. These genes showed either significant depletion or augmentation in the MDD group, suggesting that there is a realignment of gene expression at synapse under MDD condition. This shift in the expression of genes may be considered a response to activity-dependent gene regulatory mechanism in MDD. Activity-dependent gene transcription is critical in determining dynamic changes in synaptic plasticity and efficacy, neural circuits, and behavior^82,83^. Further studies will be needed to examine factors that may be associated with such a shift in gene expression at the synapse. Interestingly, a majority of the shifted genes were primarily associated with death receptor signaling. These results indicate that disruption in gene availability at the synapse may possibly be involved in programmed cell death in MDD subjects, since signaling pathways associated with apoptosis are closely linked with synaptic loss.

#### Conclusion and relevance

Collectively, for the first time, our study provides direct evidence of altered expression and realignment of genes at the synapse in MDD brain. Functional analysis strongly suggests their involvement in regulating immune response. Given the reported role of immune dysfunction in MDD, our findings are quite pertinent and suggest that synaptic genes and associated signaling mechanisms may be involved in regulating immune response in MDD. Clinically, our study is quite relevant and suggests that targeting immune function will be key at the therapeutic level. Another crucial finding of this study is the dysregulation of genes involved in programmed cell death with possible involvement in synaptic scaling. Although further studies will be needed to examine the regulatory mechanisms associated with such changes at the synapse in MDD, the findings provide evidence that identifying molecules that can repair the signaling pathways organizing cell death will be important in developing novel therapeutic tools.

## Supplementary information

Supplementary section

## Supplementary information

**Supplementary Information** The online version contains supplementary material available (10.1038/s41398-020-01159-9).
