# Supplementary material for: Molecular pathology associated with altered synaptic transcriptome in the dorsolateral prefrontal cortex of depressed subjects
Source: Transl Psychiatry. 2021 Jan 22;11:73. doi: 10.1038/s41398-020-01159-9 (PMC7822869; doi:10.1038/s41398-020-01159-9)
Supplement: Supplementary file 1 — Supplementary section [file 41398_2020_1159_MOESM1_ESM.pdf]

**Supplementary Section**

# Molecular pathology associated with altered synaptic transcriptome in the dorsolateral prefrontal cortex of depressed subjects

Yuta Yoshino,[1] Bhaskar Roy,[1] Nilesh Kumar,[2] M. Shahid Mukhtar,[2] Yogesh Dwivedi[1*]

[1]Department of Psychiatry and Behavioral Neurobiology, University of Alabama at Birmingham, Birmingham, AL 35294, USA.

[2]Department of Biology, University of Alabama at Birmingham, Birmingham, AL 35294, USA.

**Running Title:** Synaptic transcriptome and MDD pathogenesis

**Keywords:** Human postmortem brain, dorsolateral prefrontal cortex, major depressive disorder, synaptosome, gene expression, transcriptome

***Corresponding author:**

Yogesh Dwivedi, Ph.D.
Elesabeth Ridgely Shook Professor
Director of Translational Research, UAB Mood Disorder Program
Co-Director, Depression and Suicide Center
Department of Psychiatry and Behavioral Neurobiology
University of Alabama at Birmingham
SC711 Sparks Center
1720 7th Avenue South
Birmingham, Alabama, USA

Phone: 01-205-975-8459

Email: yogeshdwivedi@uabmc.edu

**Table S1. Demographic and clinical characteristics of control and MDD subjects**

| | | Control | MDD | p-value |
|---|---|---|---|---|
| Number of subjects | | 15 | 15 | N/A |
| Age (Year) | | 57.0 ± 15.0 | 57.9 ± 14.0 | 0.87 |
| Gender | Male | 8 | 7 | N/A |
| | Female | 7 | 8 | |
| Postmortem interval (Hour) | | 18.1 ± 6.0 | 17.8 ± 6.8 | 0.87 |
| Brain pH | | 6.3 ± 0.8 | 6.5 ± 0.5 | 0.40 |
| Race (white: black:other) | | 14: 1: 0 | 15: 0: 0 | N/A |
| Drug abuse | | 0 | 1 | N/A |
| Alcohol abuse | | 0 | 3 | N/A |
| Antidepressant drugs | | 0 | 6 (Amitriptyline, paroxetine, alprazolam, quetiapine, quetiapine quetiapine, doxepine, bupropion, trazodone) | N/A |
| Suicide | | 0 | 0 | N/A |
| Cause of death | | Pulmonary embolism, lymphoma, cardiac failure, lung cancer, acute myocardial infarction, cardiopulmonary arrest, cerebrovascular accident, colon cancer, leukemia, renal failure, cardiac arrest | Cardiopulmonary arrest, pneumonia, cerebrovascular accident, cardiac failure, cardiac arrest, leukemia, heart disease, multiple vehicle accident, liver failure | |

Values denote mean ± standard deviation. MDD: Major Depressive Disorder, N/A: not applicable.

**Table S2. Primer sequences for qPCR-based mRNA expression analysis**

| Primers | | Sequences |
|---|---|---|
| RASD1 | Forward | TCTGCCCTCCCAAAACTAAGC |
| | Reverse | TCCATGTTGACACCGGAACT |
| H1FX | Forward | CGGGTCCTCTCAGTCTTTCC |
| | Reverse | CAAGGCCGAGAGCCAATAGA |
| SYN1 | Forward | CCCTACTGTTGAGCCCCTTC |
| | Reverse | ATCCTTGTGGGGCTAGGTGT |
| IGFBP4 | Forward | GACCCACTCCCAAAGCTCAG |
| | Reverse | CAACCAAGCAGATGGTGCAA |
| SYN2 | Forward | AGCTGACGGTGGTATTGCTT |
| | Reverse | GACTGACACCACCCTGTAGC |
| HPSD1 | Forward | TGCTGTCATTGTCCATGCCTA |
| | Reverse | ACATGGCTCTTGTACCCAGT |
| RBBP4 | Forward | TGGCACATCAATCCGCACT |
| | Reverse | GCATGGCTGGGCACTAAAAA |
| ZFN32 | Forward | GAGACACCCTATCTGTGCGG |
| | Reverse | AAGTGGTCAAAGGGTGAGCC |
| TLR2 | Forward | ACTACGTGGATGTACCGTCA |
| | Reverse | AAACAGCACCCCAGACAAAA |
| ZFP36 | Forward | TGCCAAACCCCACCCATAAA |
| | Reverse | TACAAGGGAAGCAGACGACC |
| IRF1 | Forward | CACCAGGGCTGTCAGTTGAT |
| | Reverse | TGGAGCAGCATCTGTGTGTT |
| ELK1 | Forward | TGGAGTTGGGAGGGAGGAAT |
| | Reverse | AAATCACCAACCCCCAGGTC |
| HES5 | Forward | AGAGTCCCTGCCGTTTTAGG |
| | Reverse | GCCCCGGCACTACAAATATC |
| ASPDH | Forward | GGCTTCGATGGGGTGATTGG |
| | Reverse | CCGCTCAGCTCTACATCCAC |
| CYB561D1 | Forward | ATGCTCCTGTGTCTGGTTGG |
| | Reverse | ACACCAGTGATAGGGACGGA |
| FUT9 | Forward | GCATCCTGGGAGCAAAGCAT |
| | Reverse | TGGTTGGTAAAGTCGGCGTC |

| | | |
|---|---|---|
| GAPDH | Forward | CCACATCGCTGAGACACCAT |
| | Reverse | AGTTAAAAGCAGCCCTGGTGA |
| ACTB | Forward | AGTCATTCCAAATATGAGATGCGTT |
| | Reverse | TGCTATCACCTCCCCTGTGT |
| 18s rRNA | Forward | CGGCGACGACCCATTCGAAC |
| | Reverse | GAATCGAACCCTGATTCCCCGTC |

**Table S3. All significantly up- and down-regulated genes in the total fraction**

| Gene symbol | Ensemble ID | Locus | Fold change | p-Value | q-Value |
|---|---|---|---|---|---|
| **Up-regulated genes** | | | | | |
| TMEM189-UBE2V1 | ENSG00000124208.16_3 | chr20:48697661-48770174 | 2.093301568 | 0.028970933 | 0.998154148 |
| HNRNPUL2-BSCL2 | ENSG00000234857.2_3 | chr11:62457747-62494856 | 1.862368887 | 0.029806405 | 0.998154148 |
| SPI1 | ENSG00000066336.11_2 | chr11:47376411-47400127 | 1.674116789 | 0.016973309 | 0.998154148 |
| RASD1 | ENSG00000108551.4_2 | chr17:17397751-17399709 | 1.671422929 | 0.008487038 | 0.998154148 |
| HIST1H1D | ENSG00000124575.6_2 | chr6:26234496-26235161 | 1.6182264 | 0.021265941 | 0.998154148 |
| HSPA2 | ENSG00000126803.9_3 | chr14:65002623-65012891 | 1.587199534 | 0.03068192 | 0.998154148 |
| OLFML3 | ENSG00000116774.11_2 | chr1:114522013-114578194 | 1.566558294 | 0.015948973 | 0.998154148 |
| H1FX | ENSG00000184897.5_2 | chr3:129033614-129035120 | 1.557723521 | 0.007713264 | 0.998154148 |
| TMEM119 | ENSG00000183160.8_4 | chr12:108983622-108992096 | 1.533970067 | 0.027726499 | 0.998154148 |
| SYN2 | ENSG00000157152.16_3 | chr3:12045876-12232900 | 1.529728606 | 0.025668161 | 0.998154148 |
| SLC2A5 | ENSG00000142583.17_3 | chr1:9095166-9148537 | 1.524777663 | 0.0259714 | 0.998154148 |
| MGP | ENSG00000111341.9_2 | chr12:15034115-15038860 | 1.520322977 | 0.026291557 | 0.998154148 |
| CD248 | ENSG00000174807.3_2 | chr11:66081958-66084515 | 1.517306136 | 0.012513065 | 0.998154148 |
| SYN1 | ENSG00000008056.13_3 | chrX:47431297-47479342 | 1.514899142 | 0.010820886 | 0.998154148 |
| ADAMTS2 | ENSG00000087116.14_3 | chr5:178537852-178772431 | 1.504012857 | 0.023024431 | 0.998154148 |
| SELENON | ENSG00000162430.16_3 | chr1:26126667-26144715 | 1.502459486 | 0.018145924 | 0.998154148 |
| EHD1 | ENSG00000110047.17_2 | chr11:64619114-64655768 | 1.499851368 | 0.00026562 | 0.998154148 |
| ELK1 | ENSG00000126767.17_2 | chrX:47494920-47510003 | 1.485837489 | 0.005823038 | 0.998154148 |
| OLFML2B | ENSG00000162745.10_2 | chr1:161952982-161993644 | 1.484633409 | 0.038133823 | 0.998154148 |
| IGFBP4 | ENSG00000141753.6_2 | chr17:38599713-38613983 | 1.484402089 | 0.020842833 | 0.998154148 |
| ABI3 | ENSG00000108798.8_3 | chr17:47287589-47300587 | 1.479444697 | 0.020125555 | 0.998154148 |
| RCN3 | ENSG00000142552.7_2 | chr19:50030875-50050219 | 1.468428048 | 0.017645622 | 0.998154148 |
| C1QL1 | ENSG00000131094.3_2 | chr17:43037061-43045439 | 1.462663912 | 0.001072586 | 0.998154148 |
| CXCL16 | ENSG00000161921.14_3 | chr17:4636821-4643217 | 1.459074103 | 0.036379211 | 0.998154148 |
| LY6H | ENSG00000176956.12_2 | chr8:144239331-144242128 | 1.451994387 | 0.038359705 | 0.998154148 |
| ZNF580 | ENSG00000213015.8_3 | chr19:56146382-56154835 | 1.451031456 | 0.018192279 | 0.998154148 |
| WAS | ENSG00000015285.10_2 | chrX:48534985-48549818 | 1.441728224 | 0.02891499 | 0.998154148 |
| CITED4 | ENSG00000179862.6_2 | chr1:41326723-41328038 | 1.441314287 | 0.017935839 | 0.998154148 |
| ANKRD63 | ENSG00000230778.1_3 | chr15:40573645-40574787 | 1.440832847 | 0.003236504 | 0.998154148 |
| CPVL | ENSG00000106066.14_3 | chr7:29034847-29235067 | 1.42116355 | 0.006864688 | 0.998154148 |

| | | | | | |
|---|---|---|---|---|---|
| MACROD1 | ENSG00000133315.10_3 | chr11:63766030-63933578 | 1.416687846 | 0.004172901 | 0.998154148 |
| TGFB1 | ENSG00000105329.9_2 | chr19:41807492-41859816 | 1.407438515 | 0.048264988 | 0.998154148 |
| UTS2R | ENSG00000181408.3_2 | chr17:80332106-80333462 | 1.396622228 | 0.003928235 | 0.998154148 |
| TMEM250 | ENSG00000238227.7_4 | chr9:139006427-139010709 | 1.391864223 | 0.013786587 | 0.998154148 |
| GPR150 | ENSG00000178015.4_2 | chr5:94955782-94957846 | 1.388163585 | 0.015277308 | 0.998154148 |
| CHTF8 | ENSG00000168802.12_3 | chr16:69151913-69166487 | 1.387747039 | 0.039828071 | 0.998154148 |
| APBB1IP | ENSG00000077420.15_2 | chr10:26727132-26856732 | 1.384906718 | 0.043917747 | 0.998154148 |
| BBC3 | ENSG00000105327.17_3 | chr19:47724079-47736023 | 1.384812475 | 0.006357654 | 0.998154148 |
| ELN | ENSG00000049540.16_3 | chr7:73442119-73484237 | 1.384729315 | 0.021770765 | 0.998154148 |
| RPRM | ENSG00000177519.3_3 | chr2:154333852-154335322 | 1.375723414 | 0.036936666 | 0.998154148 |
| RAMP2 | ENSG00000131477.10_2 | chr17:40910465-40915059 | 1.374749528 | 0.047896512 | 0.998154148 |
| PRRT1 | ENSG00000204314.10_2 | chr6:32116136-32122150 | 1.364997463 | 0.02346281 | 0.998154148 |
| MAFB | ENSG00000204103.3_2 | chr20:39314488-39317880 | 1.363526305 | 0.046257386 | 0.998154148 |
| RTP5 | ENSG00000188011.5_2 | chr2:242811752-242815975 | 1.358591765 | 0.025310842 | 0.998154148 |
| FAM219A | ENSG00000164970.14_2 | chr9:34398182-34458568 | 1.35646382 | 0.03572463 | 0.998154148 |
| PIK3IP1 | ENSG00000100100.12_3 | chr22:31677579-31688520 | 1.354405462 | 0.049762571 | 0.998154148 |
| LIN37 | ENSG00000267796.7_3 | chr19:36239262-36245420 | 1.349361952 | 0.001828053 | 0.998154148 |
| LYL1 | ENSG00000104903.4_2 | chr19:13209847-13213975 | 1.344030589 | 0.011272836 | 0.998154148 |
| LTBP2 | ENSG00000119681.11_3 | chr14:74964873-75079081 | 1.342039282 | 0.035090986 | 0.998154148 |
| CD37 | ENSG00000104894.11_2 | chr19:49838428-49846592 | 1.338776193 | 0.029333429 | 0.998154148 |
| CSF3R | ENSG00000119535.17_3 | chr1:36931644-36948879 | 1.33853256 | 0.018379734 | 0.998154148 |
| WIPF3 | ENSG00000122574.10_2 | chr7:29846102-29956682 | 1.33549892 | 0.007525526 | 0.998154148 |
| ATF5 | ENSG00000169136.10_3 | chr19:50431959-50437192 | 1.33248749 | 0.025364887 | 0.998154148 |
| PRR36 | ENSG00000183248.11_2 | chr19:7933605-7939264 | 1.32961583 | 0.045489483 | 0.998154148 |
| FAM212A | ENSG00000185614.4_2 | chr3:49840687-49842463 | 1.329368537 | 0.035084598 | 0.998154148 |
| SYK | ENSG00000165025.14_2 | chr9:93564069-93660831 | 1.328146351 | 0.03231606 | 0.998154148 |
| HK3 | ENSG00000160883.10_2 | chr5:176307870-176326333 | 1.32356396 | 0.013421451 | 0.998154148 |
| KCNQ1 | ENSG00000053918.15_3 | chr11:2465914-2870339 | 1.321441032 | 0.01270184 | 0.998154148 |
| NFAM1 | ENSG00000235568.6_2 | chr22:42776416-42828401 | 1.320979112 | 0.02182532 | 0.998154148 |
| PLPPR2 | ENSG00000105520.10_2 | chr19:11466062-11476374 | 1.319630942 | 0.032818037 | 0.998154148 |
| GYPC | ENSG00000136732.14_3 | chr2:127413509-127454251 | 1.31916622 | 0.024361129 | 0.998154148 |
| GMIP | ENSG00000089639.10_2 | chr19:19740285-19754476 | 1.315489247 | 0.026706664 | 0.998154148 |
| IL10RA | ENSG00000110324.10_3 | chr11:117857063-117873752 | 1.311978622 | 0.017003244 | 0.998154148 |
| DBN1 | ENSG00000113758.13_4 | chr5:176883609-176901402 | 1.309833374 | 0.017928644 | 0.998154148 |
| CHGA | ENSG00000100604.12_4 | chr14:93389425-93401638 | 1.309101639 | 0.023071499 | 0.998154148 |

| | | | | | |
|---|---|---|---|---|---|
| MOGS | ENSG00000115275.11_3 | chr2:74688184-74692537 | 1.308928336 | 0.00086767 | 0.998154148 |
| TRNP1 | ENSG00000253368.3_3 | chr1:27320198-27327389 | 1.308170907 | 0.006174039 | 0.998154148 |
| ADGRE5 | ENSG00000123146.19_3 | chr19:14491313-14519537 | 1.307845624 | 0.032162814 | 0.998154148 |
| ARHGAP4 | ENSG00000089820.15_3 | chrX:153172821-153200452 | 1.306522635 | 0.008621848 | 0.998154148 |
| SLC9A1 | ENSG00000090020.10_2 | chr1:27425306-27493472 | 1.304350275 | 0.034164689 | 0.998154148 |
| UCP2 | ENSG00000175567.8_3 | chr11:73685712-73694352 | 1.303259123 | 0.037705574 | 0.998154148 |
| HLA-DMB | ENSG00000242574.8_3 | chr6:32902406-32908847 | 1.295387797 | 0.040308265 | 0.998154148 |
| PPP1R18 | ENSG00000146112.11_2 | chr6:30644166-30655672 | 1.294455305 | 0.019709809 | 0.998154148 |
| OSCAR | ENSG00000170909.13_2 | chr19:54597933-54606000 | 1.294417495 | 0.01439321 | 0.998154148 |
| FBXL18 | ENSG00000155034.18_3 | chr7:5470966-5553429 | 1.294226449 | 0.043758874 | 0.998154148 |
| PNRC1 | ENSG00000146278.10_2 | chr6:89790470-89794879 | 1.293081969 | 0.016021982 | 0.998154148 |
| TOX2 | ENSG00000124191.17_2 | chr20:42543504-42698256 | 1.292475229 | 0.034361075 | 0.998154148 |
| TMEM184B | ENSG00000198792.12_3 | chr22:38615298-38669040 | 1.290343169 | 0.049882827 | 0.998154148 |
| TICAM1 | ENSG00000127666.9_2 | chr19:4815944-4831716 | 1.28926153 | 0.009868033 | 0.998154148 |
| PCDHGA12 | ENSG00000253159.2_3 | chr5:140810156-140892546 | 1.288172369 | 0.041662243 | 0.998154148 |
| ZNF687 | ENSG00000143373.17_2 | chr1:151254094-151264656 | 1.287295596 | 0.003434986 | 0.998154148 |
| UNC93B1 | ENSG00000110057.7_3 | chr11:67758575-67772452 | 1.286261619 | 0.036635069 | 0.998154148 |
| CMTM3 | ENSG00000140931.19_3 | chr16:66637777-66647795 | 1.285256771 | 0.038392308 | 0.998154148 |
| CD34 | ENSG00000174059.16_4 | chr1:208054317-208084747 | 1.284705424 | 0.042190788 | 0.998154148 |
| AC098850.4 | ENSG00000266302.6_4 | chr17:16593575-16707767 | 1.283861168 | 0.038597569 | 0.998154148 |
| PRAM1 | ENSG00000133246.11_2 | chr19:8554940-8567996 | 1.282315029 | 0.015367944 | 0.998154148 |
| SLC26A11 | ENSG00000181045.14_3 | chr17:78193498-78227299 | 1.279903414 | 0.034304306 | 0.998154148 |
| RIN1 | ENSG00000174791.10_2 | chr11:66097713-66104311 | 1.275899205 | 0.005839098 | 0.998154148 |
| KDELR1 | ENSG00000105438.8_2 | chr19:48885827-48894810 | 1.274793024 | 0.028182492 | 0.998154148 |
| CEBPA | ENSG00000245848.2_2 | chr19:33790840-33793470 | 1.273480242 | 0.045344695 | 0.998154148 |
| CORO7 | ENSG00000262246.5_4 | chr16:4404543-4475706 | 1.272891144 | 0.035288201 | 0.998154148 |
| TMEM175 | ENSG00000127419.16_3 | chr4:926175-952444 | 1.272863271 | 0.01349447 | 0.998154148 |
| MED25 | ENSG00000104973.16_3 | chr19:50321536-50342073 | 1.270681745 | 0.016535929 | 0.998154148 |
| TEF | ENSG00000167074.14_2 | chr22:41763337-41795330 | 1.269899857 | 0.029924034 | 0.998154148 |
| FYB1 | ENSG00000082074.15_4 | chr5:39105338-39274630 | 1.269899446 | 0.025860073 | 0.998154148 |
| TMUB1 | ENSG00000164897.12_2 | chr7:150778167-150780633 | 1.267646457 | 0.017216651 | 0.998154148 |
| FLOT1 | ENSG00000137312.14_2 | chr6:30695486-30710510 | 1.266533143 | 0.025381547 | 0.998154148 |
| WDR81 | ENSG00000167716.18_3 | chr17:1619817-1641893 | 1.265851309 | 0.010695608 | 0.998154148 |
| FAM234A | ENSG00000167930.15_3 | chr16:284545-318970 | 1.264069786 | 0.04141908 | 0.998154148 |
| ARHGEF25 | ENSG00000240771.6_2 | chr12:58003963-58013162 | 1.262795045 | 0.027200013 | 0.998154148 |

| | | | | | |
|---|---|---|---|---|---|
| CENPVL3 | ENSG00000224109.2_3 | chrX:51360907-51361770 | 1.262008089 | 0.003213071 | 0.998154148 |
| ECHDC3 | ENSG00000134463.14_3 | chr10:11784365-11806069 | 1.261969349 | 0.013525789 | 0.998154148 |
| GRAMD1A | ENSG00000089351.14_3 | chr19:35485688-35517375 | 1.261513464 | 0.024511043 | 0.998154148 |
| SNX12 | ENSG00000147164.11_3 | chrX:70276182-70293276 | 1.25945196 | 0.039287017 | 0.998154148 |
| YPEL3 | ENSG00000090238.11_3 | chr16:30103635-30108236 | 1.258496379 | 0.029936041 | 0.998154148 |
| SF3A2 | ENSG00000104897.9_4 | chr19:2236503-2248678 | 1.258299145 | 0.028933528 | 0.998154148 |
| CHMP1A | ENSG00000131165.14_2 | chr16:89710839-89724253 | 1.257199374 | 0.04261408 | 0.998154148 |
| MS4A6A | ENSG00000110077.14_4 | chr11:59939487-59952139 | 1.256880221 | 0.039990751 | 0.998154148 |
| ZDHHC22 | ENSG00000177108.5_3 | chr14:77597613-77609077 | 1.256360962 | 0.029680791 | 0.998154148 |
| VPREB3 | ENSG00000128218.7_2 | chr22:24094930-24096655 | 1.25634214 | 0.039219667 | 0.998154148 |
| EPN3 | ENSG00000049283.17_3 | chr17:48609904-48621111 | 1.256337547 | 0.016817627 | 0.998154148 |
| Z98749.3 | ENSG00000283900.1_2 | chr22:38687924-38794527 | 1.255492154 | 0.011959624 | 0.998154148 |
| CARMIL2 | ENSG00000159753.13_3 | chr16:67678822-67691472 | 1.254393365 | 0.048002407 | 0.998154148 |
| TNFRSF10C | ENSG00000173535.13_2 | chr8:22941868-22974950 | 1.253632774 | 0.00711036 | 0.998154148 |
| ZNF503 | ENSG00000165655.16_3 | chr10:77157588-77161664 | 1.252966607 | 0.005541633 | 0.998154148 |
| FRAT1 | ENSG00000165879.8_2 | chr10:99079024-99081672 | 1.25216659 | 0.000854185 | 0.998154148 |
| CPNE5 | ENSG00000124772.11_2 | chr6:36708552-36807778 | 1.251748722 | 0.03135507 | 0.998154148 |
| IRF2BPL | ENSG00000119669.4_2 | chr14:77490886-77495042 | 1.250817775 | 0.014276884 | 0.998154148 |
| TBXA2R | ENSG00000006638.11_2 | chr19:3594504-3606838 | 1.249929615 | 0.005014844 | 0.998154148 |
| MAPK8IP2 | ENSG00000008735.13_2 | chr22:51039114-51052409 | 1.249348402 | 0.035938689 | 0.998154148 |
| PLPP1 | ENSG00000067113.16_3 | chr5:54720682-54830878 | 1.248928976 | 0.030956819 | 0.998154148 |
| TBC1D10B | ENSG00000169221.13_2 | chr16:30368423-30381585 | 1.24884791 | 0.047418348 | 0.998154148 |
| IQSEC2 | ENSG00000124313.13_3 | chrX:53255010-53350526 | 1.246952196 | 0.040924259 | 0.998154148 |
| PILRA | ENSG00000085514.15_4 | chr7:99965153-99997722 | 1.245500464 | 0.045633292 | 0.998154148 |
| IRF3 | ENSG00000126456.15_3 | chr19:50162826-50169132 | 1.244512894 | 0.016169615 | 0.998154148 |
| H1F0 | ENSG00000189060.5_2 | chr22:38201100-38203443 | 1.242274521 | 0.019109489 | 0.998154148 |
| ZGPAT | ENSG00000197114.11_3 | chr20:62338817-62367494 | 1.242126229 | 0.047414749 | 0.998154148 |
| MAP2K2 | ENSG00000126934.13_2 | chr19:4090319-4124126 | 1.241750844 | 0.026266732 | 0.998154148 |
| ZC3H3 | ENSG00000014164.6_2 | chr8:144519825-144623623 | 1.241674282 | 0.032984036 | 0.998154148 |
| HSD11B2 | ENSG00000176387.6_2 | chr16:67464555-67471456 | 1.240862038 | 0.02640214 | 0.998154148 |
| MAP7D1 | ENSG00000116871.15_4 | chr1:36621180-36646450 | 1.240647838 | 0.046467479 | 0.998154148 |
| DMRTC1B | ENSG00000184911.14_4 | chrX:71996833-72068636 | 1.239681166 | 0.014131602 | 0.998154148 |
| DCAF15 | ENSG00000132017.10_3 | chr19:14063304-14072261 | 1.239627615 | 0.027237674 | 0.998154148 |
| NEURL2 | ENSG00000124257.6_2 | chr20:44517264-44519926 | 1.237648385 | 0.045806968 | 0.998154148 |
| MEF2D | ENSG00000116604.17_3 | chr1:156433519-156470620 | 1.236800814 | 0.022173543 | 0.998154148 |

| | | | | | |
|---|---|---|---|---|---|
| ATN1 | ENSG00000111676.14_2 | chr12:7033626-7051484 | 1.236547688 | 0.049947864 | 0.998154148 |
| FAM46B | ENSG00000158246.7_2 | chr1:27331511-27339327 | 1.236543611 | 0.038720862 | 0.998154148 |
| ZNF414 | ENSG00000133250.13_2 | chr19:8574562-8579048 | 1.236031808 | 0.038160921 | 0.998154148 |
| EPN1 | ENSG00000063245.14_3 | chr19:56186592-56221224 | 1.235537574 | 0.029422358 | 0.998154148 |
| RNF26 | ENSG00000173456.4_2 | chr11:119205237-119208023 | 1.233283602 | 0.034126728 | 0.998154148 |
| DMRTC1 | ENSG00000269502.5_3 | chrX:72091859-72163643 | 1.232011759 | 0.027055565 | 0.998154148 |
| HPCAL1 | ENSG00000115756.12_3 | chr2:10443015-10567743 | 1.231646799 | 0.046724564 | 0.998154148 |
| PARVG | ENSG00000138964.16_3 | chr22:44568836-44615413 | 1.231528349 | 0.032680199 | 0.998154148 |
| TIMM29 | ENSG00000142444.6_2 | chr19:11039409-11044211 | 1.231360932 | 0.032175143 | 0.998154148 |
| CHST1 | ENSG00000175264.7_2 | chr11:45670427-45687172 | 1.230542227 | 0.008799872 | 0.998154148 |
| LILRB4 | ENSG00000186818.12_3 | chr19:55155340-55181810 | 1.22892909 | 0.041270466 | 0.998154148 |
| FAM189B | ENSG00000160767.20_3 | chr1:155216996-155225274 | 1.227694877 | 0.04889218 | 0.998154148 |
| FAM214B | ENSG00000005238.19_2 | chr9:35104109-35116338 | 1.226832726 | 0.016761706 | 0.998154148 |
| LOXL1 | ENSG00000129038.15_2 | chr15:74218330-74244478 | 1.226007718 | 0.012127077 | 0.998154148 |
| PLCB2 | ENSG00000137841.11_3 | chr15:40570377-40600136 | 1.224290889 | 0.040442992 | 0.998154148 |
| CBX4 | ENSG00000141582.14_3 | chr17:77806955-77813228 | 1.219360485 | 0.018393571 | 0.998154148 |
| REEP4 | ENSG00000168476.11_3 | chr8:21995533-21999464 | 1.219224627 | 0.048826155 | 0.998154148 |
| TMEM219 | ENSG00000149932.16_2 | chr16:29952206-29984373 | 1.218901567 | 0.045031882 | 0.998154148 |
| NUCB1 | ENSG00000104805.15_4 | chr19:49403307-49426629 | 1.218433803 | 0.043787086 | 0.998154148 |
| SCAF1 | ENSG00000126461.14_4 | chr19:50145382-50161899 | 1.213257723 | 0.01796601 | 0.998154148 |
| CCDC92 | ENSG00000119242.8_3 | chr12:124403207-124457378 | 1.212157757 | 0.034289623 | 0.998154148 |
| RAB8A | ENSG00000167461.11_3 | chr19:16222439-16245044 | 1.210984893 | 0.04918032 | 0.998154148 |
| CCDC120 | ENSG00000147144.12_3 | chrX:48911101-48927509 | 1.2108742 | 0.023224527 | 0.998154148 |
| IFFO1 | ENSG00000010295.19_3 | chr12:6647541-6665249 | 1.210380167 | 0.026730574 | 0.998154148 |
| CC2D1A | ENSG00000132024.17_3 | chr19:14017014-14041692 | 1.210156098 | 0.034319422 | 0.998154148 |
| PRR19 | ENSG00000188368.9_4 | chr19:42806250-42814973 | 1.208071838 | 0.011187886 | 0.998154148 |
| ATXN2L | ENSG00000168488.18_3 | chr16:28834356-28848558 | 1.205998217 | 0.00826407 | 0.998154148 |
| MARCH9 | ENSG00000139266.5_2 | chr12:58148881-58154190 | 1.205933707 | 0.048561097 | 0.998154148 |
| FHL3 | ENSG00000183386.9_2 | chr1:38462442-38471278 | 1.205539088 | 0.049611821 | 0.998154148 |
| IL27RA | ENSG00000104998.3_2 | chr19:14142560-14164028 | 1.205413768 | 0.038545702 | 0.998154148 |
| ZNF777 | ENSG00000196453.7_2 | chr7:149128454-149158214 | 1.205124237 | 0.035023567 | 0.998154148 |
| BAG1 | ENSG00000107262.19_4 | chr9:33247818-33264718 | 1.20502387 | 0.03454745 | 0.998154148 |
| PALM3 | ENSG00000187867.8_2 | chr19:14164177-14169971 | 1.203993191 | 0.016492135 | 0.998154148 |
| EFEMP2 | ENSG00000172638.12_3 | chr11:65633912-65641063 | 1.203885941 | 0.0409432 | 0.998154148 |
| KDF1 | ENSG00000175707.8_2 | chr1:27276053-27286897 | 1.2031965 | 0.028941187 | 0.998154148 |

| | | | | | |
|---|---|---|---|---|---|
| FAIM2 | ENSG00000135472.8_2 | chr12:50260679-50298000 | 1.201533467 | 0.046380128 | 0.998154148 |
| AC011897.1 | ENSG00000265629.1 | chr2:14775215-14790933 | 1.200024946 | 0.047682398 | 0.998154148 |
| MISP3 | ENSG00000141854.9_3 | chr19:14183348-14185874 | 1.197526506 | 0.035501877 | 0.998154148 |
| STIM1 | ENSG00000167323.11_3 | chr11:3875757-4114440 | 1.19676625 | 0.04960973 | 0.998154148 |
| AQP9 | ENSG00000103569.9_3 | chr15:58430368-58478110 | 1.196477072 | 0.044175948 | 0.998154148 |
| FCHSD1 | ENSG00000197948.10_2 | chr5:141018869-141030986 | 1.195007475 | 0.026266905 | 0.998154148 |
| DENND1C | ENSG00000205744.9_3 | chr19:6467218-6482568 | 1.193688939 | 0.028619529 | 0.998154148 |
| DPH2 | ENSG00000132768.13_2 | chr1:44435672-44439041 | 1.192547301 | 0.028123128 | 0.998154148 |
| GOLGA7B | ENSG00000155265.10_2 | chr10:99609996-99631337 | 1.190980819 | 0.049999209 | 0.998154148 |
| AKAP8 | ENSG00000105127.8_2 | chr19:15464196-15490609 | 1.189835679 | 0.013836201 | 0.998154148 |
| AL049844.1 | ENSG00000257065.1_4 | chr6:144128229-144165754 | 1.189584861 | 0.031421592 | 0.998154148 |
| ZSWIM8 | ENSG00000214655.10_3 | chr10:75545340-75561555 | 1.188953944 | 0.045516401 | 0.998154148 |
| MINK1 | ENSG00000141503.15_3 | chr17:4736683-4801356 | 1.187427802 | 0.042404185 | 0.998154148 |
| KIAA1211 | ENSG00000109265.13_4 | chr4:56915239-57194791 | 1.180047447 | 0.039572492 | 0.998154148 |
| CNOT3 | ENSG00000088038.17_3 | chr19:54641444-54659419 | 1.179581851 | 0.022284019 | 0.998154148 |
| KCNK13 | ENSG00000152315.4_3 | chr14:90528109-90652201 | 1.179488039 | 0.046428605 | 0.998154148 |
| FIGNL2 | ENSG00000261308.2_2 | chr12:52211624-52242550 | 1.178955985 | 0.00427692 | 0.998154148 |
| SYVN1 | ENSG00000162298.18_3 | chr11:64889252-64902004 | 1.178337248 | 0.047129168 | 0.998154148 |
| USP2 | ENSG00000036672.15_3 | chr11:119225925-119252436 | 1.17750312 | 0.035917434 | 0.998154148 |
| CHERP | ENSG00000085872.14_3 | chr19:16628700-16653341 | 1.174176363 | 0.020499741 | 0.998154148 |
| MKL1 | ENSG00000196588.14_3 | chr22:40806285-41032706 | 1.172087662 | 0.040356872 | 0.998154148 |
| FRAT2 | ENSG00000181274.6_2 | chr10:99092254-99094466 | 1.170560935 | 0.039615989 | 0.998154148 |
| MAPK8IP1 | ENSG00000121653.11_2 | chr11:45907202-45928016 | 1.170325522 | 0.031905401 | 0.998154148 |
| PHF13 | ENSG00000116273.5_2 | chr1:6673745-6684093 | 1.17024421 | 0.009403881 | 0.998154148 |
| CDIPT | ENSG00000103502.13_2 | chr16:29869678-29875057 | 1.169565208 | 0.022727541 | 0.998154148 |
| RSPO4 | ENSG00000101282.8_2 | chr20:939095-982907 | 1.165890678 | 0.012402888 | 0.998154148 |
| VSTM2B | ENSG00000187135.7_2 | chr19:30017406-30055386 | 1.164958043 | 0.043166071 | 0.998154148 |
| ALDH3B1 | ENSG00000006534.15_2 | chr11:67776048-67796749 | 1.164038261 | 0.049171069 | 0.998154148 |
| GCK | ENSG00000106633.15_2 | chr7:44183870-44237769 | 1.16275176 | 0.038666542 | 0.998154148 |
| ADPRHL1 | ENSG00000153531.13_3 | chr13:114053925-114107839 | 1.16130856 | 0.045462974 | 0.998154148 |
| ZMYND15 | ENSG00000141497.13_2 | chr17:4643310-4649414 | 1.156489742 | 0.036519367 | 0.998154148 |
| CALHM2 | ENSG00000138172.10_2 | chr10:105206543-105212159 | 1.156295872 | 0.037591175 | 0.998154148 |
| TINCR | ENSG00000223573.6_3 | chr19:5558178-5568045 | 1.154064039 | 0.037996827 | 0.998154148 |
| HTR1D | ENSG00000179546.4_3 | chr1:23518388-23521222 | 1.152832868 | 0.007321327 | 0.998154148 |
| UNK | ENSG00000132478.9_3 | chr17:73780852-73821880 | 1.145287907 | 0.048767977 | 0.998154148 |

| | | | | | |
|---|---|---|---|---|---|
| C19orf57 | ENSG00000132016.11_2 | chr19:13993161-14017265 | 1.141675582 | 0.01483579 | 0.998154148 |
| ZDHHC7 | ENSG00000153786.12_3 | chr16:85007787-85045141 | 1.138777196 | 0.042348993 | 0.998154148 |
| PSD4 | ENSG00000125637.15_3 | chr2:113914902-113966973 | 1.136417941 | 0.032569402 | 0.998154148 |
| ARHGAP27 | ENSG00000159314.11_3 | chr17:43471268-43511787 | 1.13016636 | 0.047166984 | 0.998154148 |
| AC245033.1 | ENSG00000260836.1_4 | chr15:83208783-83224682 | 1.117217036 | 0.035776861 | 0.998154148 |
| SPPL3 | ENSG00000157837.15_3 | chr12:121200313-121342174 | 1.116406511 | 0.025117764 | 0.998154148 |
| CIZ1 | ENSG00000148337.20_3 | chr9:130928343-130966662 | 1.107052403 | 0.012425977 | 0.998154148 |
| **Down-regulated genes** | | | | | |
| ITGAE | ENSG00000083457.11_2 | chr17:3617922-3704537 | 0.937559176 | 0.039233262 | 0.998154148 |
| MTO1 | ENSG00000135297.15_2 | chr6:74171301-74218959 | 0.910465468 | 0.034373979 | 0.998154148 |
| PRDM5 | ENSG00000138738.10_3 | chr4:121606074-121844025 | 0.90842461 | 0.019908993 | 0.998154148 |
| CMC2 | ENSG00000103121.8_3 | chr16:81000345-81053875 | 0.904900431 | 0.010098337 | 0.998154148 |
| AFG1L | ENSG00000135537.16_4 | chr6:108616098-108847999 | 0.901369232 | 0.03136285 | 0.998154148 |
| CEP83 | ENSG00000173588.14_3 | chr12:94700225-94853764 | 0.901204785 | 0.041106844 | 0.998154148 |
| TECTA | ENSG00000109927.9_3 | chr11:120971882-121062202 | 0.90053653 | 0.044549134 | 0.998154148 |
| C1orf131 | ENSG00000143633.12_3 | chr1:231359509-231376933 | 0.899590718 | 0.024883933 | 0.998154148 |
| HAUS1 | ENSG00000152240.12_2 | chr18:43684298-43708299 | 0.897269659 | 0.032566167 | 0.998154148 |
| PIN4 | ENSG00000102309.12_3 | chrX:71401203-71522776 | 0.897010706 | 0.041540967 | 0.998154148 |
| AP3S2 | ENSG00000157823.16_3 | chr15:90373831-90437870 | 0.893331601 | 0.035023666 | 0.998154148 |
| TMEM116 | ENSG00000198270.12_4 | chr12:112369086-112450989 | 0.89259114 | 0.034579064 | 0.998154148 |
| EFCAB6 | ENSG00000186976.14_3 | chr22:43924624-44208217 | 0.891949763 | 0.048979378 | 0.998154148 |
| COG2 | ENSG00000135775.13_2 | chr1:230778235-230829728 | 0.891376761 | 0.049634007 | 0.998154148 |
| APLF | ENSG00000169621.9_4 | chr2:68694693-68882994 | 0.888731908 | 0.03550356 | 0.998154148 |
| PROM1 | ENSG00000007062.11_3 | chr4:15964699-16086001 | 0.887604983 | 0.045747121 | 0.998154148 |
| PCDHA8 | ENSG00000204962.5_3 | chr5:140220772-140391929 | 0.886855433 | 0.049626064 | 0.998154148 |
| GTF2E1 | ENSG00000153767.9_3 | chr3:120461484-120501916 | 0.883307979 | 0.043196557 | 0.998154148 |
| ZNF101 | ENSG00000181896.11_3 | chr19:19779605-19794318 | 0.883139347 | 0.02450328 | 0.998154148 |
| PTRH2 | ENSG00000141378.14_2 | chr17:57751997-57784987 | 0.882647994 | 0.025902767 | 0.998154148 |
| CBFA2T2 | ENSG00000078699.21_2 | chr20:32077881-32237842 | 0.881911109 | 0.044382759 | 0.998154148 |
| KIAA0907 | ENSG00000132680.10_3 | chr1:155882834-155904191 | 0.881518112 | 0.043701589 | 0.998154148 |
| HYKK | ENSG00000188266.13_2 | chr15:78799906-78829714 | 0.880072407 | 0.02921606 | 0.998154148 |
| ZNF229 | ENSG00000278318.4_3 | chr19:44921685-44952766 | 0.879799197 | 0.020186005 | 0.998154148 |
| B3GALNT2 | ENSG00000162885.12_2 | chr1:235613238-235667781 | 0.879109274 | 0.032853327 | 0.998154148 |
| NBPF3 | ENSG00000142794.18_3 | chr1:21766621-21811498 | 0.87876567 | 0.039145107 | 0.998154148 |
| C2orf42 | ENSG00000115998.7_3 | chr2:70377012-70475747 | 0.878397634 | 0.045605554 | 0.998154148 |

| | | | | | |
|---|---|---|---|---|---|
| COQ5 | ENSG00000110871.14_3 | chr12:120941077-120972237 | 0.877445918 | 0.04988016 | 0.998154148 |
| TCAIM | ENSG00000179152.19_2 | chr3:44379611-44450943 | 0.876255874 | 0.022788154 | 0.998154148 |
| ZGRF1 | ENSG00000138658.15_3 | chr4:113460489-113558151 | 0.874672595 | 0.041201931 | 0.998154148 |
| CCDC148 | ENSG00000153237.17_2 | chr2:159027593-159313265 | 0.874317573 | 0.040879138 | 0.998154148 |
| ZNF470 | ENSG00000197016.11_3 | chr19:57078880-57100279 | 0.873837764 | 0.033241111 | 0.998154148 |
| CLCN5 | ENSG00000171365.15_3 | chrX:49687225-49863892 | 0.872365383 | 0.015183493 | 0.998154148 |
| LLPH | ENSG00000139233.6_3 | chr12:66510335-66524548 | 0.868196543 | 0.007871643 | 0.998154148 |
| INTS6L | ENSG00000165359.15_3 | chrX:134654568-134716435 | 0.868061629 | 0.042315812 | 0.998154148 |
| SRBD1 | ENSG00000068784.12_2 | chr2:45615819-45839304 | 0.867425699 | 0.033605132 | 0.998154148 |
| ZNF420 | ENSG00000197050.10_3 | chr19:37498759-37621216 | 0.867158359 | 0.038245875 | 0.998154148 |
| ZNF28 | ENSG00000198538.10_3 | chr19:53300662-53360853 | 0.867002109 | 0.049143936 | 0.998154148 |
| BRAP | ENSG00000089234.15_3 | chr12:112079950-112123790 | 0.866498758 | 0.006329511 | 0.998154148 |
| COX20 | ENSG00000203667.9_2 | chr1:244998624-245008359 | 0.865615572 | 0.041589931 | 0.998154148 |
| ZNF330 | ENSG00000109445.10_3 | chr4:142142041-142155851 | 0.863654627 | 0.04641773 | 0.998154148 |
| ZNF804B | ENSG00000182348.6_2 | chr7:88388682-88966371 | 0.86354437 | 0.00437694 | 0.998154148 |
| WDR78 | ENSG00000152763.16_2 | chr1:67278568-67390570 | 0.863384059 | 0.027051146 | 0.998154148 |
| ST6GAL2 | ENSG00000144057.15_3 | chr2:107418056-107503564 | 0.863107417 | 0.036827037 | 0.998154148 |
| INTS13 | ENSG00000064102.14_2 | chr12:27058114-27091259 | 0.862602546 | 0.033613575 | 0.998154148 |
| ING3 | ENSG00000071243.15_2 | chr7:120590803-120617270 | 0.862200451 | 0.020259214 | 0.998154148 |
| FAM184B | ENSG00000047662.4_2 | chr4:17630929-17783135 | 0.861703971 | 0.030293451 | 0.998154148 |
| ZNF749 | ENSG00000186230.6_3 | chr19:57946697-57956853 | 0.861197561 | 0.049969053 | 0.998154148 |
| NRF1 | ENSG00000106459.14_2 | chr7:129251555-129396922 | 0.861126787 | 0.029941728 | 0.998154148 |
| ZNF571 | ENSG00000180479.13_2 | chr19:38045684-38085691 | 0.859541772 | 0.038220795 | 0.998154148 |
| HAT1 | ENSG00000128708.12_2 | chr2:172778958-172848596 | 0.859147955 | 0.032231134 | 0.998154148 |
| SNX25 | ENSG00000109762.15_2 | chr4:186125391-186291339 | 0.859118875 | 0.018687806 | 0.998154148 |
| SYDE2 | ENSG00000097096.8_2 | chr1:85622556-85666729 | 0.856872991 | 0.035663879 | 0.998154148 |
| EXOSC8 | ENSG00000120699.12_3 | chr13:37572953-37583750 | 0.8565462 | 0.042749302 | 0.998154148 |
| C5 | ENSG00000106804.7_2 | chr9:123714613-123812554 | 0.856292725 | 0.011729918 | 0.998154148 |
| WNK3 | ENSG00000196632.10_3 | chrX:54219256-54385075 | 0.855529679 | 0.036951048 | 0.998154148 |
| ZBTB26 | ENSG00000171448.8_2 | chr9:125677845-125693779 | 0.855460372 | 0.048767298 | 0.998154148 |
| ZNF143 | ENSG00000166478.9_3 | chr11:9481866-9550071 | 0.855069345 | 0.016542077 | 0.998154148 |
| TMEM138 | ENSG00000149483.11_2 | chr11:61129473-61136981 | 0.854565311 | 0.02570802 | 0.998154148 |
| RPIA | ENSG00000153574.8_2 | chr2:88991162-89050452 | 0.853072317 | 0.036995561 | 0.998154148 |
| SPRED2 | ENSG00000198369.9_3 | chr2:65537985-65659771 | 0.852810015 | 0.04134861 | 0.998154148 |
| ZNF549 | ENSG00000121406.8_2 | chr19:58038693-58068910 | 0.852415333 | 0.027971884 | 0.998154148 |

| | | | | | |
|---|---|---|---|---|---|
| TBCCD1 | ENSG00000113838.12_3 | chr3:186263862-186288332 | 0.852282031 | 0.022906767 | 0.998154148 |
| ZSCAN21 | ENSG00000166529.14_2 | chr7:99647397-99662661 | 0.852144054 | 0.005769904 | 0.998154148 |
| ZNF10 | ENSG00000256223.5_3 | chr12:133707161-133736051 | 0.852033858 | 0.049823058 | 0.998154148 |
| SEL1L3 | ENSG00000091490.10_3 | chr4:25749049-25865382 | 0.851896384 | 0.030046817 | 0.998154148 |
| GNPAT | ENSG00000116906.11_3 | chr1:231376953-231413719 | 0.851323487 | 0.046747047 | 0.998154148 |
| PYROXD1 | ENSG00000121350.15_2 | chr12:21590549-21624186 | 0.850813366 | 0.026805023 | 0.998154148 |
| DCAF17 | ENSG00000115827.13_3 | chr2:172290727-172341562 | 0.850640374 | 0.022532351 | 0.998154148 |
| DHFR2 | ENSG00000178700.7_3 | chr3:93766680-93782233 | 0.850253114 | 0.011485906 | 0.998154148 |
| ZNF322 | ENSG00000181315.10_3 | chr6:26634611-26659980 | 0.850106466 | 0.039396684 | 0.998154148 |
| WASHC3 | ENSG00000120860.10_3 | chr12:102406705-102455927 | 0.850001287 | 0.024321285 | 0.998154148 |
| S100PBP | ENSG00000116497.17_3 | chr1:33282368-33324480 | 0.849946658 | 0.045894145 | 0.998154148 |
| THADA | ENSG00000115970.18_3 | chr2:43457975-43823185 | 0.84859352 | 0.013666855 | 0.998154148 |
| NUDT15 | ENSG00000136159.3_2 | chr13:48611703-48621358 | 0.848000137 | 0.041287143 | 0.998154148 |
| CNTNAP3B | ENSG00000154529.10 | chr9:43684902-43924049 | 0.847475962 | 0.039200767 | 0.998154148 |
| POLR1B | ENSG00000125630.15_2 | chr2:113299492-113334727 | 0.847428265 | 0.021118645 | 0.998154148 |
| TTPA | ENSG00000137561.4_2 | chr8:63961112-63998612 | 0.84488278 | 0.037238954 | 0.998154148 |
| RBM34 | ENSG00000188739.14_3 | chr1:235294498-235324772 | 0.844852633 | 0.025783746 | 0.998154148 |
| SOX6 | ENSG00000110693.17_3 | chr11:15987995-16761138 | 0.844156122 | 0.016625836 | 0.998154148 |
| ZNF610 | ENSG00000167554.14_2 | chr19:52839498-52871031 | 0.843991324 | 0.024844326 | 0.998154148 |
| KIAA1143 | ENSG00000163807.5_2 | chr3:44779153-44803154 | 0.843458346 | 0.047586775 | 0.998154148 |
| FAAH2 | ENSG00000165591.6_2 | chrX:57313139-57515629 | 0.842938279 | 0.033542833 | 0.998154148 |
| UBXN8 | ENSG00000104691.14_3 | chr8:30589764-30624522 | 0.842705342 | 0.03706639 | 0.998154148 |
| NECTIN3 | ENSG00000177707.10_3 | chr3:110788918-110994410 | 0.841803775 | 0.021271187 | 0.998154148 |
| FXN | ENSG00000165060.11_3 | chr9:71650175-71715094 | 0.841145026 | 0.006870013 | 0.998154148 |
| MNAT1 | ENSG00000020426.10_3 | chr14:61201460-61436671 | 0.84060604 | 0.03447681 | 0.998154148 |
| SDHC | ENSG00000143252.14_2 | chr1:161284047-161345130 | 0.840134938 | 0.017708017 | 0.998154148 |
| ZNF761 | ENSG00000160336.14_3 | chr19:53935246-53961515 | 0.83957076 | 0.042689333 | 0.998154148 |
| ZNF468 | ENSG00000204604.9_3 | chr19:53341261-53360902 | 0.838670553 | 0.0214953 | 0.998154148 |
| KLHL20 | ENSG00000076321.10_3 | chr1:173684080-173755840 | 0.83782626 | 0.011677379 | 0.998154148 |
| U2AF1L5 | ENSG00000275895.6_4 | chr21:44513066-44527693 | 0.837420787 | 0.033815163 | 0.998154148 |
| NT5E | ENSG00000135318.11_3 | chr6:86159302-86205509 | 0.835936472 | 0.015883428 | 0.998154148 |
| ZNF415 | ENSG00000170954.11_4 | chr19:53611132-53636330 | 0.835774411 | 0.036544448 | 0.998154148 |
| ZNF883 | ENSG00000228623.5_1 | chr9:115720002-115812323 | 0.835513559 | 0.035661451 | 0.998154148 |
| BDNF | ENSG00000176697.18_3 | chr11:27676440-27743605 | 0.835200945 | 0.03308505 | 0.998154148 |
| TIMMDC1 | ENSG00000113845.9_2 | chr3:119217379-119243937 | 0.835075439 | 0.022781485 | 0.998154148 |

| | | | | | |
|---|---|---|---|---|---|
| EPB41L5 | ENSG00000115109.13_2 | chr2:120770581-120936695 | 0.834755246 | 0.039226633 | 0.998154148 |
| FAM206A | ENSG00000119328.11_2 | chr9:111696461-111713024 | 0.833922498 | 0.026167117 | 0.998154148 |
| PRKD1 | ENSG00000184304.14_4 | chr14:30045685-30661104 | 0.831883887 | 0.028437524 | 0.998154148 |
| CWC25 | ENSG00000273559.4_2 | chr17:36956687-36981734 | 0.831754483 | 0.010681401 | 0.998154148 |
| MARC1 | ENSG00000186205.12_3 | chr1:220960101-220992999 | 0.831715965 | 0.018499295 | 0.998154148 |
| KIAA0586 | ENSG00000100578.14_4 | chr14:58894103-59018007 | 0.831543164 | 0.034692463 | 0.998154148 |
| PLPBP | ENSG00000147471.11_4 | chr8:37620111-37637285 | 0.831473656 | 0.043022808 | 0.998154148 |
| LRP2BP | ENSG00000109771.15_2 | chr4:186285033-186317053 | 0.830633737 | 0.040152033 | 0.998154148 |
| FBXO16 | ENSG00000214050.7_3 | chr8:28205804-28347835 | 0.829598375 | 0.02478746 | 0.998154148 |
| PAAF1 | ENSG00000175575.12_2 | chr11:73587744-73642169 | 0.829479609 | 0.007253391 | 0.998154148 |
| KIF27 | ENSG00000165115.14_2 | chr9:86451613-86536380 | 0.828087838 | 0.030180324 | 0.998154148 |
| VWC2L | ENSG00000174453.9_3 | chr2:215275789-215443683 | 0.82793781 | 0.0294608 | 0.998154148 |
| PXMP2 | ENSG00000176894.9_3 | chr12:133264192-133281577 | 0.827659652 | 0.041337544 | 0.998154148 |
| TNNT1 | ENSG00000105048.16_2 | chr19:55644162-55660722 | 0.827234609 | 0.036510138 | 0.998154148 |
| DCHS2 | ENSG00000197410.12_1 | chr4:155153189-155412868 | 0.827081705 | 0.02899233 | 0.998154148 |
| BRD7 | ENSG00000166164.15_3 | chr16:50347398-50402845 | 0.826976017 | 0.034620297 | 0.998154148 |
| PEX12 | ENSG00000108733.9_3 | chr17:33901814-33905882 | 0.826933835 | 0.025111113 | 0.998154148 |
| ZNF562 | ENSG00000171466.9_3 | chr19:9752484-9785762 | 0.826073297 | 0.028263248 | 0.998154148 |
| MSH6 | ENSG00000116062.14_3 | chr2:47922669-48037240 | 0.824938523 | 0.023279416 | 0.998154148 |
| MRPS22 | ENSG00000175110.11_3 | chr3:138724648-139076065 | 0.823281569 | 0.003584798 | 0.998154148 |
| COMMD10 | ENSG00000145781.8_2 | chr5:115420688-115748459 | 0.82320552 | 0.019276292 | 0.998154148 |
| S100A13 | ENSG00000189171.14_3 | chr1:153591263-153603836 | 0.822323012 | 0.016706959 | 0.998154148 |
| TSPAN12 | ENSG00000106025.8_2 | chr7:120427374-120498456 | 0.821454208 | 0.032565656 | 0.998154148 |
| MRPL47 | ENSG00000136522.13_2 | chr3:179306073-179322442 | 0.820240262 | 0.034058717 | 0.998154148 |
| RBM28 | ENSG00000106344.8_3 | chr7:127937738-127983962 | 0.820027942 | 0.0409239 | 0.998154148 |
| SMG7 | ENSG00000116698.21_3 | chr1:183441351-183567381 | 0.819881274 | 0.032186278 | 0.998154148 |
| C12orf49 | ENSG00000111412.5_2 | chr12:117147990-117175875 | 0.819357723 | 0.017175047 | 0.998154148 |
| SLC25A33 | ENSG00000171612.6_3 | chr1:9599541-9645237 | 0.819276085 | 0.041476807 | 0.998154148 |
| LYRM2 | ENSG00000083099.10_2 | chr6:90277863-90348472 | 0.817864529 | 0.008061349 | 0.998154148 |
| PHACTR2 | ENSG00000112419.14_3 | chr6:143857982-144152322 | 0.817641259 | 0.02680242 | 0.998154148 |
| ADHFE1 | ENSG00000147576.15_3 | chr8:67342420-67383836 | 0.817189796 | 0.008036173 | 0.998154148 |
| ZNF334 | ENSG00000198185.11_3 | chr20:45128269-45142198 | 0.816737908 | 0.019624702 | 0.998154148 |
| TAF11 | ENSG00000064995.16_2 | chr6:34845555-34855866 | 0.816081323 | 0.025533362 | 0.998154148 |
| UBXN2A | ENSG00000173960.13_3 | chr2:24150155-24227779 | 0.815206282 | 0.008464835 | 0.998154148 |
| ZNF614 | ENSG00000142556.18_3 | chr19:52516018-52533493 | 0.812879662 | 0.011409011 | 0.998154148 |

| | | | | | |
|---|---|---|---|---|---|
| UTP6 | ENSG00000108651.9_2 | chr17:30187918-30228784 | 0.812707045 | 0.018120549 | 0.998154148 |
| PLCH1 | ENSG00000114805.16_3 | chr3:155093369-155462856 | 0.812145362 | 0.044987961 | 0.998154148 |
| PRELID3A | ENSG00000141391.13_3 | chr18:12407895-12432237 | 0.810555804 | 0.022078299 | 0.998154148 |
| ZFP3 | ENSG00000180787.5_2 | chr17:4981543-4999669 | 0.809872741 | 0.005821418 | 0.998154148 |
| NDUFAF7 | ENSG00000003509.15_2 | chr2:37458774-37480546 | 0.809738934 | 0.028668135 | 0.998154148 |
| LRRC6 | ENSG00000129295.8_2 | chr8:133584201-133687863 | 0.807175474 | 0.048213135 | 0.998154148 |
| ZW10 | ENSG00000086827.8_3 | chr11:113603909-113644533 | 0.807066572 | 0.014907383 | 0.998154148 |
| MIS12 | ENSG00000167842.15_2 | chr17:5389605-5394134 | 0.806987816 | 0.008428619 | 0.998154148 |
| TRIB2 | ENSG00000071575.11_2 | chr2:12857015-12882860 | 0.804887983 | 0.040491981 | 0.998154148 |
| AC004076.1 | ENSG00000268163.1_4 | chr19:57949338-57988904 | 0.804581253 | 0.017412907 | 0.998154148 |
| RGPD3 | ENSG00000153165.18_2 | chr2:107007746-107084832 | 0.803091307 | 0.043208367 | 0.998154148 |
| AL121845.3 | ENSG00000273154.3_4 | chr20:62340216-62370456 | 0.80126645 | 0.037421084 | 0.998154148 |
| SRP19 | ENSG00000153037.13_3 | chr5:112196919-112205485 | 0.800697338 | 0.0489681 | 0.998154148 |
| HSD17B6 | ENSG00000025423.11_3 | chr12:57145945-57181574 | 0.800373574 | 0.039449677 | 0.998154148 |
| AC018630.6 | ENSG00000275778.1_4 | chr12:10998448-11324197 | 0.800002824 | 0.015909879 | 0.998154148 |
| RPF2 | ENSG00000197498.12_2 | chr6:111303218-111349466 | 0.79908781 | 0.044183911 | 0.998154148 |
| PAK1IP1 | ENSG00000111845.4_2 | chr6:10694928-10710015 | 0.797721913 | 0.013261023 | 0.998154148 |
| VWA8 | ENSG00000102763.17_3 | chr13:42140973-42535256 | 0.797470431 | 0.007075448 | 0.998154148 |
| SPRY2 | ENSG00000136158.11_3 | chr13:80910111-80915086 | 0.795991401 | 0.011348033 | 0.998154148 |
| ETV1 | ENSG00000006468.13_2 | chr7:13930853-14031050 | 0.793589732 | 0.02766108 | 0.998154148 |
| PDSS1 | ENSG00000148459.15_3 | chr10:26986588-27035727 | 0.791703822 | 0.01275175 | 0.998154148 |
| ZNF302 | ENSG00000089335.20_3 | chr19:35168544-35177302 | 0.791464577 | 0.025360148 | 0.998154148 |
| USPL1 | ENSG00000132952.11_2 | chr13:31191830-31234907 | 0.788301055 | 0.041974079 | 0.998154148 |
| USP18 | ENSG00000184979.9_2 | chr22:18632666-18660164 | 0.787897784 | 0.003205706 | 0.998154148 |
| CEBPZ | ENSG00000115816.14_3 | chr2:37428755-37458856 | 0.787511339 | 0.045209765 | 0.998154148 |
| BLZF1 | ENSG00000117475.13_2 | chr1:169337208-169365778 | 0.786436941 | 0.033047401 | 0.998154148 |
| SELENOT | ENSG00000198843.12_3 | chr3:150320662-150348232 | 0.780810525 | 0.030589203 | 0.998154148 |
| MPHOSPH8 | ENSG00000196199.13_2 | chr13:20207821-20247599 | 0.778988661 | 0.039299165 | 0.998154148 |
| ZNF484 | ENSG00000127081.13_2 | chr9:95607313-95640320 | 0.778441553 | 0.032061778 | 0.998154148 |
| C8orf4 | ENSG00000176907.4_2 | chr8:40010974-40012827 | 0.77821368 | 0.006847759 | 0.998154148 |
| PDCD6IP | ENSG00000170248.13_2 | chr3:33839844-33911199 | 0.777223064 | 0.006442322 | 0.998154148 |
| TMEM254 | ENSG00000133678.13_3 | chr10:81838402-81852313 | 0.776132899 | 0.00496647 | 0.998154148 |
| TAS2R5 | ENSG00000127366.5_2 | chr7:141490017-141491167 | 0.775416025 | 0.042789881 | 0.998154148 |
| LRRC37A | ENSG00000176681.14_2 | chr17:44370099-44415160 | 0.774183314 | 0.0381071 | 0.998154148 |
| SLC4A8 | ENSG00000050438.16_2 | chr12:51785101-51909547 | 0.772767428 | 0.041781165 | 0.998154148 |

| | | | | | |
|---|---|---|---|---|---|
| DPH3 | ENSG00000154813.9_2 | chr3:16299485-16306479 | 0.771048729 | 0.00336707 | 0.998154148 |
| TMEM126A | ENSG00000171202.6_2 | chr11:85359011-85367591 | 0.768098469 | 0.029954166 | 0.998154148 |
| FGD6 | ENSG00000180263.13_3 | chr12:95470525-95611258 | 0.767550409 | 0.032563425 | 0.998154148 |
| BDH2 | ENSG00000164039.14_3 | chr4:104000592-104021040 | 0.76618433 | 0.026098732 | 0.998154148 |
| AL513165.2 | ENSG00000256966.6_4 | chr9:37512544-37592466 | 0.765501781 | 0.037636186 | 0.998154148 |
| ZNF32 | ENSG00000169740.13_2 | chr10:44139307-44144304 | 0.764235273 | 0.008265639 | 0.998154148 |
| ENDOU | ENSG00000111405.8_2 | chr12:48103517-48119350 | 0.76297606 | 0.008070261 | 0.998154148 |
| SLN | ENSG00000170290.3_2 | chr11:107578104-107590419 | 0.762697314 | 0.030907623 | 0.998154148 |
| OR2W3 | ENSG00000238243.3_2 | chr1:248058889-248059833 | 0.761683655 | 0.021253529 | 0.998154148 |
| DNAJA4 | ENSG00000140403.12_2 | chr15:78556428-78574538 | 0.76013059 | 0.040997887 | 0.998154148 |
| LRRC37A2 | ENSG00000238083.7_2 | chr17:44588877-44633016 | 0.75918872 | 0.029176016 | 0.998154148 |
| CCDC167 | ENSG00000198937.8_2 | chr6:37450696-37467698 | 0.758738525 | 0.049893105 | 0.998154148 |
| OTOGL | ENSG00000165899.10_2 | chr12:80603233-80772870 | 0.758383143 | 0.0073387 | 0.998154148 |
| FP15737 | ENSG00000215298.3 | chr8:23430157-23432974 | 0.751653359 | 0.029617859 | 0.998154148 |
| ACAD11 | ENSG00000240303.7_3 | chr3:132276982-132379567 | 0.750470175 | 0.048358226 | 0.998154148 |
| RBBP4 | ENSG00000162521.18_3 | chr1:33116743-33151812 | 0.749822389 | 0.020815195 | 0.998154148 |
| HSPD1 | ENSG00000144381.16_3 | chr2:198351305-198381461 | 0.745771365 | 0.023242035 | 0.998154148 |
| TOGARAM1 | ENSG00000198718.12_3 | chr14:45431411-45543634 | 0.743039018 | 0.009521289 | 0.998154148 |
| GSTM5 | ENSG00000134201.10_3 | chr1:110254877-110318050 | 0.740939805 | 0.035152072 | 0.998154148 |
| FSBP | ENSG00000265817.2_3 | chr8:95384398-95449180 | 0.739829097 | 0.019761943 | 0.998154148 |
| PPARA | ENSG00000186951.16_3 | chr22:46546424-46639653 | 0.736169585 | 0.044314345 | 0.998154148 |
| QSER1 | ENSG00000060749.14_2 | chr11:32914724-33014862 | 0.735464221 | 0.038886837 | 0.998154148 |
| FABP6 | ENSG00000170231.15_2 | chr5:159614374-159665742 | 0.731863888 | 0.028418238 | 0.998154148 |
| ZNF709 | ENSG00000242852.6_3 | chr19:12571998-12624668 | 0.71556927 | 0.007476814 | 0.998154148 |
| RALB | ENSG00000144118.13_3 | chr2:120997640-121052289 | 0.714954298 | 0.025116048 | 0.998154148 |
| DUSP6 | ENSG00000139318.7_2 | chr12:89741009-89747048 | 0.713571153 | 0.024744029 | 0.998154148 |
| MDM4 | ENSG00000198625.12_2 | chr1:204485507-204527248 | 0.70941623 | 0.013522487 | 0.998154148 |
| FCF1 | ENSG00000119616.11_2 | chr14:75179847-75205323 | 0.702405537 | 0.008291242 | 0.998154148 |
| NME1-NME2 | ENSG00000011052.21_3 | chr17:49230951-49249105 | 0.651236153 | 0.049573833 | 0.998154148 |
| MKLN1 | ENSG00000128585.17_3 | chr7:130794855-131181395 | 0.630768248 | 0.014691013 | 0.998154148 |
| AL662899.3 | ENSG00000263020.6_4 | chr6:31633879-31641323 | 0.522821095 | 0.014578824 | 0.998154148 |
| AD000671.1 | ENSG00000188223.9_4 | chr19:36236579-36245420 | 0.491543271 | 0.015996805 | 0.998154148 |

**Table S4. All significantly up- and down-regulated genes in the synaptic fraction**

| Gene symbol | Ensemble ID | Locus | Fold change | p-Value | q-Value |
|---|---|---|---|---|---|
| **Up-regulated genes** | | | | | |
| ZFP36 | ENSG00000128016.5_2 | chr19:39897453-39900052 | 2.1776576 | 0.0275288 | 0.999912394 |
| C1QB | ENSG00000173369.15_2 | chr1:22979255-22988031 | 2.0394579 | 0.0465707 | 0.999912394 |
| CHTF8 | ENSG00000168802.12_3 | chr16:69151913-69166487 | 1.8470062 | 0.0174307 | 0.999912394 |
| RASD1 | ENSG00000108551.4_2 | chr17:17397751-17399709 | 1.8322651 | 0.0081353 | 0.999912394 |
| CEBPD | ENSG00000221869.4_2 | chr8:48649471-48651648 | 1.7427126 | 0.0399716 | 0.999912394 |
| TMEM88B | ENSG00000205116.3_2 | chr1:1361508-1363167 | 1.7405742 | 0.0093953 | 0.999912394 |
| KIF19 | ENSG00000196169.14_2 | chr17:72322349-72351959 | 1.7176642 | 0.0061926 | 0.999912394 |
| MOG | ENSG00000204655.11_3 | chr6:29624758-29640149 | 1.6982876 | 0.0446928 | 0.999912394 |
| S100A9 | ENSG00000163220.10_2 | chr1:153330330-153333503 | 1.6963496 | 0.0214211 | 0.999912394 |
| RGS16 | ENSG00000143333.6_2 | chr1:182567758-182573543 | 1.6860759 | 0.020914 | 0.999912394 |
| HAPLN2 | ENSG00000132702.12_2 | chr1:156589086-156595517 | 1.660522 | 0.0399065 | 0.999912394 |
| CX3CL1 | ENSG00000006210.6_2 | chr16:57406370-57418960 | 1.6509677 | 0.0164296 | 0.999912394 |
| GALNT15 | ENSG00000131386.17_2 | chr3:16216156-16273499 | 1.6278187 | 0.0268205 | 0.999912394 |
| SLC2A5 | ENSG00000142583.17_3 | chr1:9095166-9148537 | 1.6249166 | 0.003212 | 0.999912394 |
| ADNP2 | ENSG00000101544.8_2 | chr18:77866915-77905406 | 1.5810882 | 0.0164974 | 0.999912394 |
| CD68 | ENSG00000129226.13_3 | chr17:7482785-7485431 | 1.5771296 | 0.0423986 | 0.999912394 |
| FPR1 | ENSG00000171051.8_3 | chr19:52248425-52307363 | 1.5668887 | 0.007887 | 0.999912394 |
| CDC42EP1 | ENSG00000128283.6_4 | chr22:37956454-37965412 | 1.5649011 | 0.0222008 | 0.999912394 |
| TYROBP | ENSG00000011600.11_2 | chr19:36395303-36399197 | 1.5585077 | 0.022949 | 0.999912394 |
| PRIMA1 | ENSG00000175785.12_2 | chr14:94184644-94254827 | 1.5531521 | 0.0295804 | 0.999912394 |
| SMOC1 | ENSG00000198732.10_2 | chr14:70320848-70499083 | 1.5367045 | 0.0062641 | 0.999912394 |
| HIST1H3D | ENSG00000197409.7_3 | chr6:26197068-26197478 | 1.5350196 | 0.0125672 | 0.999912394 |
| AL136376.1 | ENSG00000269279.1 | chr1:116915890-116926718 | 1.5307629 | 0.0418539 | 0.999912394 |
| CBR1 | ENSG00000159228.12_2 | chr21:37442239-37445464 | 1.5095717 | 0.0023686 | 0.999912394 |
| AC006486.1 | ENSG00000268643.1_4 | chr19:42738735-42759284 | 1.5061942 | 0.0058379 | 0.999912394 |
| PRR18 | ENSG00000176381.5_3 | chr6:166718788-166721936 | 1.5019354 | 0.0063391 | 0.999912394 |
| EDIL3 | ENSG00000164176.12_3 | chr5:83236373-83680611 | 1.4997978 | 0.0429222 | 0.999912394 |
| ITGB2 | ENSG00000160255.17_3 | chr21:46305868-46351904 | 1.4975076 | 0.0450782 | 0.999912394 |
| GPIHBP1 | ENSG00000277494.1_2 | chr8:144295068-144299044 | 1.4970042 | 0.0161025 | 0.999912394 |
| RTKN | ENSG00000114993.16_2 | chr2:74652963-74669549 | 1.4941954 | 0.0054524 | 0.999912394 |

| | | | | | |
|---|---|---|---|---|---|
| SLC11A1 | ENSG00000018280.16_2 | chr2:219246752-219261617 | 1.478482 | 0.0411049 | 0.999912394 |
| SPI1 | ENSG00000066336.11_2 | chr11:47376411-47400127 | 1.4699304 | 0.0291721 | 0.999912394 |
| LDB3 | ENSG00000122367.19_3 | chr10:88428206-88495825 | 1.4638139 | 0.0148954 | 0.999912394 |
| RHOU | ENSG00000116574.5_2 | chr1:228870824-228882416 | 1.4572597 | 0.0028964 | 0.999912394 |
| HCLS1 | ENSG00000180353.10_2 | chr3:121350246-121379774 | 1.4534723 | 0.0302514 | 0.999912394 |
| GPX1 | ENSG00000233276.3_3 | chr3:49394609-49396033 | 1.4522081 | 0.0247716 | 0.999912394 |
| AC073896.1 | ENSG00000144785.8_4 | chr12:56679700-56709843 | 1.4457229 | 0.0296458 | 0.999912394 |
| SH3PXD2B | ENSG00000174705.12_3 | chr5:171752004-171881527 | 1.4452584 | 0.0450207 | 0.999912394 |
| TSPAN15 | ENSG00000099282.9_3 | chr10:71211229-71267425 | 1.4395893 | 0.0331081 | 0.999912394 |
| FERMT3 | ENSG00000149781.12_3 | chr11:63974150-63991358 | 1.4365096 | 0.039016 | 0.999912394 |
| ECHDC3 | ENSG00000134463.14_3 | chr10:11784365-11806069 | 1.4311422 | 0.0008299 | 0.999912394 |
| DEF6 | ENSG00000023892.10_2 | chr6:35265629-35289548 | 1.422364 | 0.0065991 | 0.999912394 |
| PHLDB1 | ENSG00000019144.18_3 | chr11:118477155-118528748 | 1.411454 | 0.0223944 | 0.999912394 |
| CXCL16 | ENSG00000161921.14_3 | chr17:4636821-4643217 | 1.4082337 | 0.0339933 | 0.999912394 |
| HBEGF | ENSG00000113070.7_2 | chr5:139712428-139726216 | 1.4071037 | 0.0178945 | 0.999912394 |
| MAP2K3 | ENSG00000034152.18_3 | chr17:21187984-21218552 | 1.4012996 | 0.0122844 | 0.999912394 |
| AKIRIN1 | ENSG00000174574.15_3 | chr1:39456895-39471731 | 1.394551 | 0.0175371 | 0.999912394 |
| INSIG1 | ENSG00000186480.12_3 | chr7:155089486-155101945 | 1.3942983 | 0.0419544 | 0.999912394 |
| PIM1 | ENSG00000137193.13_2 | chr6:37137979-37143202 | 1.3910995 | 0.0439518 | 0.999912394 |
| CD59 | ENSG00000085063.14_3 | chr11:33719807-33757991 | 1.3892392 | 0.0317052 | 0.999912394 |
| OPALIN | ENSG00000197430.10_2 | chr10:98102973-98119122 | 1.3824273 | 0.0413411 | 0.999912394 |
| SHROOM4 | ENSG00000158352.15_2 | chrX:50334647-50557302 | 1.3807606 | 0.0400913 | 0.999912394 |
| DBNDD2 | ENSG00000244274.7_3 | chr20:44034697-44039250 | 1.3799971 | 0.0368782 | 0.999912394 |
| GPNMB | ENSG00000136235.15_3 | chr7:23275586-23314727 | 1.3790575 | 0.0212324 | 0.999912394 |
| TRAF4 | ENSG00000076604.14_3 | chr17:27071002-27077976 | 1.3779663 | 0.0069576 | 0.999912394 |
| SIGLEC9 | ENSG00000129450.8_2 | chr19:51628165-51639908 | 1.3767954 | 0.0321491 | 0.999912394 |
| GSN | ENSG00000148180.19_3 | chr9:123970072-124095121 | 1.3707911 | 0.0346081 | 0.999912394 |
| PDE4B | ENSG00000184588.17_3 | chr1:66258197-66840262 | 1.3698842 | 0.0454108 | 0.999912394 |
| ELOVL1 | ENSG00000066322.14_3 | chr1:43829068-43833745 | 1.3657649 | 0.047326 | 0.999912394 |
| H1F0 | ENSG00000189060.5_2 | chr22:38201100-38203443 | 1.3602022 | 0.0042927 | 0.999912394 |
| SGK2 | ENSG00000101049.14_3 | chr20:42187608-42216877 | 1.3572334 | 0.0484886 | 0.999912394 |
| WIPF1 | ENSG00000115935.17_4 | chr2:175424300-175547644 | 1.353282 | 0.0052749 | 0.999912394 |
| PXDC1 | ENSG00000168994.13_2 | chr6:3722848-3752260 | 1.3525253 | 0.0457249 | 0.999912394 |
| C5AR1 | ENSG00000197405.7_3 | chr19:47793280-47825323 | 1.3483603 | 0.0297668 | 0.999912394 |
| RNASET2 | ENSG00000026297.15_3 | chr6:167342992-167370679 | 1.3462312 | 0.0442161 | 0.999912394 |

| | | | | | |
|---|---|---|---|---|---|
| ZBED3 | ENSG00000132846.5_4 | chr5:76367897-76383148 | 1.3454267 | 0.0008996 | 0.999912394 |
| PIP4K2A | ENSG00000150867.13_3 | chr10:22823778-23003484 | 1.3430789 | 0.0118545 | 0.999912394 |
| GSG1L | ENSG00000169181.12_4 | chr16:27798856-28074830 | 1.3406162 | 0.0007414 | 0.999912394 |
| SNU13 | ENSG00000100138.13_2 | chr22:42069934-42086508 | 1.338581 | 0.0128383 | 0.999912394 |
| RASSF2 | ENSG00000101265.15_2 | chr20:4760669-4804291 | 1.3380231 | 0.0125459 | 0.999912394 |
| CDKN1C | ENSG00000129757.12_2 | chr11:2904443-2907111 | 1.3369348 | 0.0446486 | 0.999912394 |
| ZBTB43 | ENSG00000169155.9_3 | chr9:129567285-129600489 | 1.3366042 | 0.0234785 | 0.999912394 |
| MTA2 | ENSG00000149480.6_2 | chr11:62360686-62369312 | 1.3318732 | 0.039548 | 0.999912394 |
| MAL | ENSG00000172005.10_2 | chr2:95691422-95719741 | 1.3315349 | 0.0474162 | 0.999912394 |
| SLCO1A2 | ENSG00000084453.16_3 | chr12:21417534-21572528 | 1.3277406 | 0.0111242 | 0.999912394 |
| SOX8 | ENSG00000005513.9_2 | chr16:1031808-1036979 | 1.3246712 | 0.012971 | 0.999912394 |
| SERINC5 | ENSG00000164300.16_2 | chr5:79407474-79551898 | 1.3231909 | 0.0397174 | 0.999912394 |
| SLC25A1 | ENSG00000100075.9_2 | chr22:19163088-19166343 | 1.3223847 | 0.0257429 | 0.999912394 |
| SNAI1 | ENSG00000124216.3_2 | chr20:48599536-48605423 | 1.3216293 | 0.0250616 | 0.999912394 |
| N4BP2L1 | ENSG00000139597.17_3 | chr13:32974860-33002448 | 1.3213328 | 0.0269505 | 0.999912394 |
| TLR2 | ENSG00000137462.6_2 | chr4:154622652-154626851 | 1.3203869 | 0.0323141 | 0.999912394 |
| GTF2H3 | ENSG00000111358.13_3 | chr12:124118286-124147153 | 1.3202115 | 0.0160122 | 0.999912394 |
| OTUD7B | ENSG00000264522.5_2 | chr1:149909705-149982625 | 1.3174402 | 0.0370509 | 0.999912394 |
| RELA | ENSG00000173039.18_3 | chr11:65421067-65430565 | 1.3140115 | 0.0256881 | 0.999912394 |
| MSLN | ENSG00000102854.15_3 | chr16:810762-818865 | 1.3129353 | 0.0317411 | 0.999912394 |
| SPATA13 | ENSG00000182957.15_3 | chr13:24553944-24881212 | 1.3113199 | 0.027528 | 0.999912394 |
| WASF2 | ENSG00000158195.10_2 | chr1:27730730-27816669 | 1.3082764 | 0.032357 | 0.999912394 |
| TNFRSF10B | ENSG00000120889.12_2 | chr8:22877646-22926692 | 1.3079724 | 0.0392618 | 0.999912394 |
| IRF2 | ENSG00000168310.10_3 | chr4:185308867-185395734 | 1.3031339 | 0.0051633 | 0.999912394 |
| B3GNT9 | ENSG00000237172.3_2 | chr16:67182008-67185117 | 1.3028737 | 0.0483316 | 0.999912394 |
| MICALL1 | ENSG00000100139.13_3 | chr22:38301664-38338829 | 1.3026413 | 0.0102863 | 0.999912394 |
| CDC42SE1 | ENSG00000197622.12_2 | chr1:151023447-151042801 | 1.3025141 | 0.043694 | 0.999912394 |
| NUAK2 | ENSG00000163545.8_2 | chr1:205271187-205290919 | 1.2999699 | 0.0153594 | 0.999912394 |
| SSH3 | ENSG00000172830.12_3 | chr11:67070919-67080078 | 1.2998803 | 0.0098709 | 0.999912394 |
| TFEB | ENSG00000112561.17_3 | chr6:41651716-41703997 | 1.2944792 | 0.0053913 | 0.999912394 |
| PTPRH | ENSG00000080031.9_3 | chr19:55692616-55720874 | 1.2943289 | 0.0371086 | 0.999912394 |
| FCHSD1 | ENSG00000197948.10_2 | chr5:141018869-141030986 | 1.2928176 | 0.0212076 | 0.999912394 |
| PTPRC | ENSG00000081237.18_3 | chr1:198607801-198726412 | 1.2926396 | 0.0250106 | 0.999912394 |
| IRF1 | ENSG00000125347.13_3 | chr5:131817301-131826490 | 1.2895121 | 0.049636 | 0.999912394 |
| NFAM1 | ENSG00000235568.6_2 | chr22:42776416-42828401 | 1.2886583 | 0.0359893 | 0.999912394 |

| | | | | | |
|---|---|---|---|---|---|
| JPT2 | ENSG00000206053.12_4 | chr16:1728257-1752281 | 1.2886282 | 0.0119669 | 0.999912394 |
| SMAD9 | ENSG00000120693.13_3 | chr13:37418968-37494902 | 1.2882305 | 0.0147515 | 0.999912394 |
| SH3BP4 | ENSG00000130147.15_3 | chr2:235860617-235964358 | 1.28803 | 0.0174714 | 0.999912394 |
| INPP1 | ENSG00000151689.12_2 | chr2:191208196-191236391 | 1.2871267 | 0.043256 | 0.999912394 |
| ELF1 | ENSG00000120690.15_3 | chr13:41506164-41635576 | 1.2832428 | 0.0154785 | 0.999912394 |
| CORO1B | ENSG00000172725.13_3 | chr11:67202981-67211292 | 1.2821114 | 0.0320307 | 0.999912394 |
| CHMP2B | ENSG00000083937.8_2 | chr3:87276421-87304698 | 1.2804737 | 0.0230209 | 0.999912394 |
| ELK1 | ENSG00000126767.17_2 | chrX:47494920-47510003 | 1.2792851 | 0.0494165 | 0.999912394 |
| NDRG1 | ENSG00000104419.14_4 | chr8:134249414-134314265 | 1.2788627 | 0.0467802 | 0.999912394 |
| KIF13B | ENSG00000197892.12_2 | chr8:28924796-29120641 | 1.2781564 | 0.0141405 | 0.999912394 |
| FAM198A | ENSG00000144649.8_4 | chr3:43020759-43101703 | 1.276496 | 0.0247417 | 0.999912394 |
| TMEM98 | ENSG00000006042.11_2 | chr17:31254928-31272124 | 1.2762245 | 0.0481177 | 0.999912394 |
| ADGRE5 | ENSG00000123146.19_3 | chr19:14491313-14519537 | 1.2758758 | 0.0162438 | 0.999912394 |
| ADIPOR2 | ENSG00000006831.9_2 | chr12:1797740-1897844 | 1.2755488 | 0.0287699 | 0.999912394 |
| H2AFJ | ENSG00000246705.4_3 | chr12:14927317-14930936 | 1.2740466 | 0.0205642 | 0.999912394 |
| DUSP16 | ENSG00000111266.8_3 | chr12:12627144-12715317 | 1.2739273 | 0.0112589 | 0.999912394 |
| AC009690.1 | ENSG00000260729.1_4 | chr15:72577068-72668322 | 1.2736923 | 0.0467613 | 0.999912394 |
| MSR1 | ENSG00000038945.14_3 | chr8:15965387-16424999 | 1.2736773 | 0.0492673 | 0.999912394 |
| LCP1 | ENSG00000136167.13_3 | chr13:46700055-46786006 | 1.2736568 | 0.0283315 | 0.999912394 |
| TMCC3 | ENSG00000057704.12_3 | chr12:94960900-95044338 | 1.2729291 | 0.0284203 | 0.999912394 |
| BHLHE41 | ENSG00000123095.5_2 | chr12:26272959-26278060 | 1.2717088 | 0.0085049 | 0.999912394 |
| RWDD3 | ENSG00000122481.16_3 | chr1:95699711-95712781 | 1.2711802 | 0.0471943 | 0.999912394 |
| HNRNPA1L2 | ENSG00000139675.11_3 | chr13:53214921-53217931 | 1.2695678 | 0.0144244 | 0.999912394 |
| C11orf57 | ENSG00000150776.17_3 | chr11:111944810-111955874 | 1.2688734 | 0.0100756 | 0.999912394 |
| SHMT1 | ENSG00000176974.19_3 | chr17:18231174-18266877 | 1.2677934 | 0.0410607 | 0.999912394 |
| LEMD2 | ENSG00000161904.11_3 | chr6:33738979-33756913 | 1.2642032 | 0.045934 | 0.999912394 |
| RPS15 | ENSG00000115268.9_4 | chr19:1438357-1440493 | 1.2641858 | 0.0312018 | 0.999912394 |
| SLA | ENSG00000155926.13_3 | chr8:134048969-134115298 | 1.2603458 | 0.0320541 | 0.999912394 |
| NPC2 | ENSG00000119655.10_3 | chr14:74942895-74960880 | 1.2570853 | 0.0346297 | 0.999912394 |
| TCP11L2 | ENSG00000166046.10_3 | chr12:106695707-106740793 | 1.2566249 | 0.0280612 | 0.999912394 |
| JMJD4 | ENSG00000081692.12_2 | chr1:227918126-227923112 | 1.2547875 | 0.0138267 | 0.999912394 |
| ST20-MTHFS | ENSG00000259332.3_3 | chr15:80137492-80216096 | 1.2538705 | 0.0465473 | 0.999912394 |
| RNASE4 | ENSG00000258818.3_3 | chr14:21152259-21169374 | 1.2534573 | 0.0148222 | 0.999912394 |
| ERRFI1 | ENSG00000116285.12_2 | chr1:8064464-8086368 | 1.2530838 | 0.0370586 | 0.999912394 |
| SHKBP1 | ENSG00000160410.14_3 | chr19:41082757-41097305 | 1.252883 | 0.0349054 | 0.999912394 |

| | | | | | |
|---|---|---|---|---|---|
| PLA2G16 | ENSG00000176485.11_3 | chr11:63340667-63384355 | 1.2526898 | 0.0395807 | 0.999912394 |
| NDE1 | ENSG00000072864.14_3 | chr16:15737124-15820210 | 1.2505641 | 0.0420034 | 0.999912394 |
| SF3A2 | ENSG00000104897.9_4 | chr19:2236503-2248678 | 1.2494376 | 0.013301 | 0.999912394 |
| IL17RA | ENSG00000177663.13_2 | chr22:17565844-17596584 | 1.2487521 | 0.0225229 | 0.999912394 |
| TOR4A | ENSG00000198113.2_2 | chr9:140172201-140177093 | 1.2480499 | 0.0192268 | 0.999912394 |
| FAM228A | ENSG00000186453.12_4 | chr2:24397938-24423718 | 1.2479403 | 0.0174632 | 0.999912394 |
| ADRA2B | ENSG00000274286.1_2 | chr2:96778623-96781984 | 1.2478672 | 0.0273155 | 0.999912394 |
| H1FX | ENSG00000184897.5_2 | chr3:129033614-129035120 | 1.2478233 | 0.0110975 | 0.999912394 |
| WTAP | ENSG00000146457.14_2 | chr6:160146617-160177351 | 1.2474898 | 0.0412624 | 0.999912394 |
| TRAFD1 | ENSG00000135148.11_3 | chr12:112563305-112591413 | 1.2458801 | 0.0146729 | 0.999912394 |
| FAM107B | ENSG00000065809.13_3 | chr10:14560556-14816896 | 1.2447128 | 0.0191543 | 0.999912394 |
| CCNG1 | ENSG00000113328.18_2 | chr5:162864575-162873157 | 1.2428177 | 0.0178573 | 0.999912394 |
| CPVL | ENSG00000106066.14_3 | chr7:29034847-29235067 | 1.2419374 | 0.0382903 | 0.999912394 |
| FYB1 | ENSG00000082074.15_4 | chr5:39105338-39274630 | 1.2391096 | 0.0303154 | 0.999912394 |
| PSENEN | ENSG00000205155.7_3 | chr19:36236015-36238420 | 1.2386774 | 0.0445087 | 0.999912394 |
| MIIP | ENSG00000116691.10_2 | chr1:12079523-12092102 | 1.2380441 | 0.0292537 | 0.999912394 |
| PARVG | ENSG00000138964.16_3 | chr22:44568836-44615413 | 1.2379095 | 0.0213258 | 0.999912394 |
| CSF3R | ENSG00000119535.17_3 | chr1:36931644-36948879 | 1.237778 | 0.0238275 | 0.999912394 |
| APH1A | ENSG00000117362.12_3 | chr1:150237799-150241980 | 1.2367637 | 0.0093605 | 0.999912394 |
| CDH19 | ENSG00000071991.8_2 | chr18:64168320-64271375 | 1.2355281 | 0.03839 | 0.999912394 |
| LIPT2 | ENSG00000175536.6_2 | chr11:74202757-74204778 | 1.2346426 | 0.0380753 | 0.999912394 |
| TAB2 | ENSG00000055208.18_3 | chr6:149539777-149732749 | 1.234626 | 0.0303709 | 0.999912394 |
| PALM3 | ENSG00000187867.8_2 | chr19:14164177-14169971 | 1.2323999 | 0.0250486 | 0.999912394 |
| TRIM62 | ENSG00000116525.13_2 | chr1:33611003-33647660 | 1.2310591 | 0.0237846 | 0.999912394 |
| ZNF821 | ENSG00000102984.14_3 | chr16:71893583-71929239 | 1.2299161 | 0.0487599 | 0.999912394 |
| MED30 | ENSG00000164758.7_2 | chr8:118532952-118552501 | 1.2291014 | 0.0479023 | 0.999912394 |
| MTSS1 | ENSG00000170873.18_3 | chr8:125563031-125740670 | 1.2281944 | 0.0312497 | 0.999912394 |
| CHD2 | ENSG00000173575.20_1 | chr15:93443419-93571235 | 1.2274986 | 0.0455669 | 0.999912394 |
| HNRNPF | ENSG00000169813.16_3 | chr10:43881065-43904614 | 1.227477 | 0.0416568 | 0.999912394 |
| MYD88 | ENSG00000172936.12_3 | chr3:38179969-38184513 | 1.2266494 | 0.0430256 | 0.999912394 |
| COL9A2 | ENSG00000049089.13_3 | chr1:40766159-40783488 | 1.2255108 | 0.0403993 | 0.999912394 |
| EMILIN2 | ENSG00000132205.10_2 | chr18:2847028-2915991 | 1.2252446 | 0.0092872 | 0.999912394 |
| RGL2 | ENSG00000237441.9_2 | chr6:33259431-33267101 | 1.2247001 | 0.0106655 | 0.999912394 |
| TCF12 | ENSG00000140262.17_2 | chr15:57210821-57591479 | 1.2246067 | 0.0305264 | 0.999912394 |
| MCM5 | ENSG00000100297.15_3 | chr22:35796056-35821423 | 1.2226446 | 0.0233467 | 0.999912394 |

| | | | | | |
|---|---|---|---|---|---|
| PHC2 | ENSG00000134686.18_3 | chr1:33789224-33896653 | 1.2205689 | 0.0040414 | 0.999912394 |
| FAM114A1 | ENSG00000197712.11_2 | chr4:38869298-38947360 | 1.2204702 | 0.0271639 | 0.999912394 |
| TBXAS1 | ENSG00000059377.16_2 | chr7:139476850-139720125 | 1.2176727 | 0.0345651 | 0.999912394 |
| ZNF570 | ENSG00000171827.10_2 | chr19:37958487-37979554 | 1.2173453 | 0.0226402 | 0.999912394 |
| GDE1 | ENSG00000006007.11_2 | chr16:19513011-19533467 | 1.2167078 | 0.0278638 | 0.999912394 |
| BMP8B | ENSG00000116985.11_3 | chr1:40222854-40254533 | 1.2160911 | 0.0294302 | 0.999912394 |
| SLC4A2 | ENSG00000164889.13_3 | chr7:150754297-150773614 | 1.2135736 | 0.0302291 | 0.999912394 |
| RPS6KA2 | ENSG00000071242.11_3 | chr6:166822852-167319939 | 1.2109142 | 0.0109757 | 0.999912394 |
| EIF3F | ENSG00000175390.13_3 | chr11:7991798-8023409 | 1.2101928 | 0.0264594 | 0.999912394 |
| FOXO3 | ENSG00000118689.14_2 | chr6:108881038-109005977 | 1.2096335 | 0.0474803 | 0.999912394 |
| FBXL8 | ENSG00000135722.8_3 | chr16:67193834-67198473 | 1.2068191 | 0.0183952 | 0.999912394 |
| ZNF217 | ENSG00000171940.13_3 | chr20:52183604-52226446 | 1.2067404 | 0.0063415 | 0.999912394 |
| JOSD2 | ENSG00000161677.11_3 | chr19:51009255-51014610 | 1.2047893 | 0.0127898 | 0.999912394 |
| RUNX1 | ENSG00000159216.18_3 | chr21:36160098-37376965 | 1.2047371 | 0.0247299 | 0.999912394 |
| LACTB2 | ENSG00000147592.8_2 | chr8:71547553-71581409 | 1.1988347 | 0.0271241 | 0.999912394 |
| BMF | ENSG00000104081.13_3 | chr15:40380091-40401093 | 1.1977482 | 0.0485777 | 0.999912394 |
| PPP1R35 | ENSG00000160813.6_2 | chr7:100032905-100034188 | 1.1957616 | 0.0338099 | 0.999912394 |
| ZEB2 | ENSG00000169554.18_3 | chr2:145121931-145282150 | 1.1954504 | 0.0353838 | 0.999912394 |
| AC018755.1 | ENSG00000167765.3 | chr19:52095036-52097630 | 1.19383 | 0.0488274 | 0.999912394 |
| TUT1 | ENSG00000149016.15_3 | chr11:62342517-62359649 | 1.1934012 | 0.0438199 | 0.999912394 |
| MED25 | ENSG00000104973.16_3 | chr19:50321536-50342073 | 1.1892178 | 0.022476 | 0.999912394 |
| TMEM187 | ENSG00000177854.7_3 | chrX:153237778-153248646 | 1.1887293 | 0.0487107 | 0.999912394 |
| DISC1 | ENSG00000162946.21_4 | chr1:231762561-232177018 | 1.1857924 | 0.0110872 | 0.999912394 |
| NRBP2 | ENSG00000185189.17_3 | chr8:144915764-144923146 | 1.1838847 | 0.0368363 | 0.999912394 |
| FNBP1 | ENSG00000187239.16_2 | chr9:132649466-132805473 | 1.1837077 | 0.0282587 | 0.999912394 |
| STAT5A | ENSG00000126561.16_2 | chr17:40439565-40463961 | 1.180101 | 0.0371641 | 0.999912394 |
| TIFA | ENSG00000145365.10_2 | chr4:113195698-113207059 | 1.178567 | 0.0376155 | 0.999912394 |
| SELENOM | ENSG00000198832.10_2 | chr22:31500758-31516055 | 1.1785199 | 0.0434439 | 0.999912394 |
| ARL16 | ENSG00000214087.8_3 | chr17:79648204-79650954 | 1.177453 | 0.03142 | 0.999912394 |
| GTPBP1 | ENSG00000100226.15_3 | chr22:39101728-39134304 | 1.1753528 | 0.0135741 | 0.999912394 |
| HAUS4 | ENSG00000092036.18_4 | chr14:23415437-23426370 | 1.1747808 | 0.0072245 | 0.999912394 |
| ARHGAP27 | ENSG00000159314.11_3 | chr17:43471268-43511787 | 1.1734611 | 0.0469769 | 0.999912394 |
| GXYLT2 | ENSG00000172986.12_3 | chr3:72937224-73047289 | 1.1708746 | 0.0223666 | 0.999912394 |
| MACROD1 | ENSG00000133315.10_3 | chr11:63766030-63933578 | 1.1696237 | 0.0475393 | 0.999912394 |
| SMPDL3A | ENSG00000172594.12_2 | chr6:123110194-123130865 | 1.1692425 | 0.0446456 | 0.999912394 |

| | | | | | |
|---|---|---|---|---|---|
| BLVRB | ENSG00000090013.9_2 | chr19:40953696-40971747 | 1.1641493 | 0.0294404 | 0.999912394 |
| RCAN1 | ENSG00000159200.17_2 | chr21:35885440-35987441 | 1.1594898 | 0.0339671 | 0.999912394 |
| SNIP1 | ENSG00000163877.10_2 | chr1:38000050-38019945 | 1.1587107 | 0.0407049 | 0.999912394 |
| GTF2F2 | ENSG00000188342.11_3 | chr13:45694650-45859044 | 1.1553077 | 0.0138344 | 0.999912394 |
| KCTD5 | ENSG00000167977.8_3 | chr16:2732476-2759031 | 1.1511217 | 0.0410237 | 0.999912394 |
| LSM1 | ENSG00000175324.9_2 | chr8:38020839-38034248 | 1.147908 | 0.0453231 | 0.999912394 |
| LLGL2 | ENSG00000073350.13_2 | chr17:73521161-73571289 | 1.1455037 | 0.0497083 | 0.999912394 |
| KRBA2 | ENSG00000184619.3_3 | chr17:8271955-8280029 | 1.1452456 | 0.0460014 | 0.999912394 |
| BAZ2A | ENSG00000076108.11_2 | chr12:56989380-57030600 | 1.1441418 | 0.0482752 | 0.999912394 |
| CDK19 | ENSG00000155111.14_3 | chr6:110931181-111137161 | 1.142423 | 0.0318343 | 0.999912394 |
| WHAMM | ENSG00000156232.7_2 | chr15:83478380-83504860 | 1.1388265 | 0.0204501 | 0.999912394 |
| TBC1D1 | ENSG00000065882.15_3 | chr4:37892708-38140796 | 1.1370191 | 0.0418677 | 0.999912394 |
| TCEAL3 | ENSG00000196507.10_3 | chrX:102862379-102884618 | 1.1365208 | 0.0453117 | 0.999912394 |
| RIN3 | ENSG00000100599.15_4 | chr14:92980118-93155339 | 1.1312604 | 0.044281 | 0.999912394 |
| RALY | ENSG00000125970.11_4 | chr20:32581452-32696114 | 1.127119 | 0.0485894 | 0.999912394 |
| CC2D1A | ENSG00000132024.17_3 | chr19:14017014-14041692 | 1.1255187 | 0.0372756 | 0.999912394 |
| UBE2D2 | ENSG00000131508.15_2 | chr5:138906016-139008018 | 1.125508 | 0.0484474 | 0.999912394 |
| C14orf93 | ENSG00000100802.14_3 | chr14:23455117-23479375 | 1.1185911 | 0.0385821 | 0.999912394 |
| PHF11 | ENSG00000136147.17_4 | chr13:50069746-50103123 | 1.1184468 | 0.0259917 | 0.999912394 |
| CCDC9 | ENSG00000105321.12_2 | chr19:47759237-47775210 | 1.1153723 | 0.0415504 | 0.999912394 |
| SPAG1 | ENSG00000104450.12_2 | chr8:101170134-101271506 | 1.1132633 | 0.0342163 | 0.999912394 |
| KLHL36 | ENSG00000135686.12_2 | chr16:84682131-84701292 | 1.0996935 | 0.0386563 | 0.999912394 |
| ATXN1L | ENSG00000224470.7_3 | chr16:71879894-71919171 | 1.0728368 | 0.0473205 | 0.999912394 |
| **Down-regulated genes** | | | | | |
| RNASEH2B | ENSG00000136104.19_3 | chr13:51483814-51547881 | 0.9263577 | 0.0496634 | 0.999912394 |
| DNAJC24 | ENSG00000170946.14_2 | chr11:31391387-31453396 | 0.9192889 | 0.0274706 | 0.999912394 |
| AP3S2 | ENSG00000157823.16_3 | chr15:90373831-90437870 | 0.9112092 | 0.0498719 | 0.999912394 |
| TET1 | ENSG00000138336.8_3 | chr10:70320413-70454239 | 0.9024881 | 0.0076316 | 0.999912394 |
| ZSCAN29 | ENSG00000140265.12_2 | chr15:43650370-43663223 | 0.8990058 | 0.046785 | 0.999912394 |
| ZNF713 | ENSG00000178665.14_3 | chr7:55955168-56009918 | 0.8873668 | 0.0164467 | 0.999912394 |
| PDE7B | ENSG00000171408.13_3 | chr6:136172834-136516712 | 0.8871713 | 0.0490762 | 0.999912394 |
| ZFP3 | ENSG00000180787.5_2 | chr17:4981543-4999669 | 0.8855522 | 0.0246304 | 0.999912394 |
| ISPD | ENSG00000214960.9_2 | chr7:16127152-16460947 | 0.8830823 | 0.0429459 | 0.999912394 |
| ASAP2 | ENSG00000151693.10_3 | chr2:9346894-9545812 | 0.8790239 | 0.0426633 | 0.999912394 |
| LZIC | ENSG00000162441.11_2 | chr1:9982171-10003465 | 0.8785773 | 0.0350735 | 0.999912394 |

| | | | | | |
|---|---|---|---|---|---|
| KMT5B | ENSG00000110066.14_3 | chr11:67922330-67981295 | 0.8762556 | 0.0410923 | 0.999912394 |
| LYRM2 | ENSG00000083099.10_2 | chr6:90277863-90348472 | 0.8757792 | 0.0216332 | 0.999912394 |
| SGSM3 | ENSG00000100359.20_3 | chr22:40766595-40806293 | 0.874915 | 0.0405052 | 0.999912394 |
| GIN1 | ENSG00000145723.16_2 | chr5:102421704-102455855 | 0.8702349 | 0.0176353 | 0.999912394 |
| CDIP1 | ENSG00000089486.16_3 | chr16:4560676-4588829 | 0.8639487 | 0.0192545 | 0.999912394 |
| MARC1 | ENSG00000186205.12_3 | chr1:220960101-220992999 | 0.863248 | 0.0133391 | 0.999912394 |
| RNF214 | ENSG00000167257.10_3 | chr11:117103341-117157161 | 0.8631369 | 0.0106245 | 0.999912394 |
| PCDHB7 | ENSG00000113212.6_2 | chr5:140552200-140555964 | 0.8596802 | 0.0464523 | 0.999912394 |
| TRAPPC5 | ENSG00000181029.8_3 | chr19:7745729-7752589 | 0.8586476 | 0.0372299 | 0.999912394 |
| SYPL2 | ENSG00000143028.8_2 | chr1:110009250-110024759 | 0.8583532 | 0.0025033 | 0.999912394 |
| GSTM2 | ENSG00000213366.12_3 | chr1:110210644-110252173 | 0.8556602 | 0.0315312 | 0.999912394 |
| THSD1 | ENSG00000136114.15_2 | chr13:52951302-52980629 | 0.845616 | 0.0217433 | 0.999912394 |
| SGCD | ENSG00000170624.13_2 | chr5:155297354-156194799 | 0.8447133 | 0.0351057 | 0.999912394 |
| PLCB3 | ENSG00000149782.11_2 | chr11:64018995-64036622 | 0.8445156 | 0.0303356 | 0.999912394 |
| SEL1L3 | ENSG00000091490.10_3 | chr4:25749049-25865382 | 0.8441025 | 0.0024603 | 0.999912394 |
| SLC35A4 | ENSG00000176087.14_2 | chr5:139944041-139948688 | 0.8382002 | 0.0421184 | 0.999912394 |
| VILL | ENSG00000136059.14_2 | chr3:38029550-38048679 | 0.8355835 | 0.0189474 | 0.999912394 |
| SCGB3A1 | ENSG00000161055.3_2 | chr5:180017103-180018540 | 0.8351407 | 0.0489781 | 0.999912394 |
| TCERG1L | ENSG00000176769.9_2 | chr10:132890654-133109984 | 0.8339763 | 0.0461175 | 0.999912394 |
| CLUAP1 | ENSG00000103351.12_3 | chr16:3550924-3589048 | 0.8274534 | 0.036996 | 0.999912394 |
| DPH3 | ENSG00000154813.9_2 | chr3:16299485-16306479 | 0.8206404 | 0.0066713 | 0.999912394 |
| MON1A | ENSG00000164077.13_4 | chr3:49946295-49967606 | 0.8193118 | 0.0366616 | 0.999912394 |
| CACHD1 | ENSG00000158966.13_2 | chr1:64936475-65158741 | 0.8180571 | 0.0310342 | 0.999912394 |
| NT5E | ENSG00000135318.11_3 | chr6:86159302-86205509 | 0.8171443 | 0.018534 | 0.999912394 |
| TRPM3 | ENSG00000083067.22_3 | chr9:73143979-74061820 | 0.8157727 | 0.0352362 | 0.999912394 |
| SHROOM2 | ENSG00000146950.12_2 | chrX:9754496-9917483 | 0.8083461 | 0.0260327 | 0.999912394 |
| ADHFE1 | ENSG00000147576.15_3 | chr8:67342420-67383836 | 0.8032816 | 0.0306443 | 0.999912394 |
| AC091167.7 | ENSG00000275674.1_4 | chr15:90809549-90857731 | 0.801337 | 0.0433393 | 0.999912394 |
| SPATA24 | ENSG00000170469.10_4 | chr5:138732252-138739777 | 0.7986994 | 0.0050226 | 0.999912394 |
| CDH4 | ENSG00000179242.15_4 | chr20:59827482-60515673 | 0.7962247 | 0.019576 | 0.999912394 |
| CLDN10 | ENSG00000134873.9_2 | chr13:96085858-96232013 | 0.7947775 | 0.0254516 | 0.999912394 |
| N6AMT1 | ENSG00000156239.11_2 | chr21:30244513-30257693 | 0.794196 | 0.0192057 | 0.999912394 |
| FUT9 | ENSG00000172461.10_2 | chr6:96463860-96663488 | 0.7924129 | 0.0261338 | 0.999912394 |
| FZD8 | ENSG00000177283.7_3 | chr10:35927177-35931206 | 0.7911683 | 0.0373176 | 0.999912394 |
| MTA3 | ENSG00000057935.13_2 | chr2:42721709-42984087 | 0.7883872 | 0.0229091 | 0.999912394 |

| | | | | | |
|---|---|---|---|---|---|
| CISD3 | ENSG00000277972.1_2 | chr17:36886488-36891297 | 0.7833351 | 0.0087768 | 0.999912394 |
| WNT7B | ENSG00000188064.9_3 | chr22:46316242-46373009 | 0.7770486 | 0.0398209 | 0.999912394 |
| MRPL34 | ENSG00000130312.6_3 | chr19:17403418-17417652 | 0.7698633 | 0.0371592 | 0.999912394 |
| ASPDH | ENSG00000204653.9_3 | chr19:51014857-51017947 | 0.7658521 | 0.0327412 | 0.999912394 |
| TTPA | ENSG00000137561.4_2 | chr8:63961112-63998612 | 0.7616707 | 0.0046591 | 0.999912394 |
| LFNG | ENSG00000106003.12_2 | chr7:2552163-2568811 | 0.7578215 | 0.0298689 | 0.999912394 |
| KCNJ14 | ENSG00000182324.6_3 | chr19:48958766-48970237 | 0.7523269 | 0.0071809 | 0.999912394 |
| CHST7 | ENSG00000147119.3_2 | chrX:46433219-46457843 | 0.7443651 | 0.0226256 | 0.999912394 |
| CYB561D1 | ENSG00000174151.14_4 | chr1:110036674-110045554 | 0.7427098 | 0.0254082 | 0.999912394 |
| TEN1 | ENSG00000257949.6_3 | chr17:73975301-73996667 | 0.7386488 | 0.0370837 | 0.999912394 |
| CHP1 | ENSG00000187446.11_2 | chr15:41523037-41574088 | 0.7310931 | 0.0382482 | 0.999912394 |
| ARNT2 | ENSG00000172379.20_3 | chr15:80696692-80890278 | 0.7235799 | 0.0358096 | 0.999912394 |
| ACSS1 | ENSG00000154930.14_2 | chr20:24986866-25039616 | 0.7132899 | 0.0013848 | 0.999912394 |
| HES5 | ENSG00000197921.5_2 | chr1:2460184-2461684 | 0.5447051 | 0.0328057 | 0.999912394 |

**Table S5. Gene Ontology analysis performed with up-regulated genes (> 1.3-fold) in synaptic fraction**

| GO ID | Term annotated | Number of genes | Fold enrichment | p-value | FDR |
|---|---|---|---|---|---|
| **Biological Process (BP)** | | | | | |
| GO:0110089 | Regulation of hippocampal neuron apoptotic process | 2 | 99.98 | 3.63E-04 | 4.29E-02 |
| GO:0071223 | Cellular response to lipoteichoic acid | 3 | 59.99 | 3.30E-05 | 9.85E-03 |
| GO:0070391 | Response to lipoteichoic acid | 3 | 59.99 | 3.30E-05 | 9.67E-03 |
| GO:0002523 | Leukocyte migration involved in inflammatory response | 3 | 46.15 | 6.40E-05 | 1.51E-02 |
| GO:0002269 | Leukocyte activation involved in inflammatory response | 4 | 36.36 | 7.95E-06 | 4.84E-03 |
| GO:0001774 | Microglial cell activation | 4 | 36.36 | 7.95E-06 | 4.66E-03 |
| GO:0007250 | Activation of NF-kappab-inducing kinase activity | 3 | 33.33 | 1.49E-04 | 2.29E-02 |
| GO:1903978 | Regulation of microglial cell activation | 3 | 29.99 | 1.97E-04 | 2.78E-02 |
| GO:0010894 | Negative regulation of steroid biosynthetic process | 3 | 26.08 | 2.86E-04 | 3.71E-02 |
| GO:0061900 | Glial cell activation | 4 | 25.8 | 2.69E-05 | 8.35E-03 |
| GO:0042116 | Macrophage activation | 5 | 24.39 | 3.23E-06 | 2.43E-03 |
| GO:0150076 | Neuroinflammatory response | 4 | 24.24 | 3.37E-05 | 9.51E-03 |
| GO:0045939 | Negative regulation of steroid metabolic process | 3 | 24 | 3.58E-04 | 4.26E-02 |
| GO:1901224 | Positive regulation of NIK/NF-kappab signaling | 5 | 13.16 | 5.26E-05 | 1.34E-02 |
| GO:0030593 | Neutrophil chemotaxis | 5 | 12.19 | 7.41E-05 | 1.65E-02 |
| GO:0071621 | Granulocyte chemotaxis | 5 | 11.23 | 1.07E-04 | 1.92E-02 |
| GO:1901222 | Regulation of NIK/NF-kappab signaling | 6 | 11.21 | 2.16E-05 | 7.11E-03 |
| GO:1990266 | Neutrophil migration | 5 | 10.99 | 1.18E-04 | 2.03E-02 |
| GO:0097530 | Granulocyte migration | 5 | 10.1 | 1.72E-04 | 2.60E-02 |
| GO:0042035 | Regulation of cytokine biosynthetic process | 5 | 9.9 | 1.89E-04 | 2.74E-02 |
| GO:0032680 | Regulation of tumor necrosis factor production | 6 | 8.82 | 7.78E-05 | 1.71E-02 |
| GO:1903555 | Regulation of tumor necrosis factor superfamily cytokine production | 6 | 8.57 | 9.07E-05 | 1.86E-02 |
| GO:0030595 | Leukocyte chemotaxis | 6 | 8.51 | 9.42E-05 | 1.86E-02 |
| GO:0002221 | Pattern recognition receptor signaling pathway | 5 | 8.13 | 4.52E-04 | 4.83E-02 |
| GO:0097529 | Myeloid leukocyte migration | 5 | 8.06 | 4.68E-04 | 4.93E-02 |
| GO:0051092 | Positive regulation of NF-kappab transcription factor activity | 6 | 7.89 | 1.40E-04 | 2.24E-02 |
| GO:0030099 | Myeloid cell differentiation | 8 | 7.58 | 1.39E-05 | 5.80E-03 |
| GO:0071219 | Cellular response to molecule of bacterial origin | 7 | 7.29 | 6.23E-05 | 1.52E-02 |
| GO:0060326 | Cell chemotaxis | 7 | 6.83 | 9.26E-05 | 1.88E-02 |
| GO:0034341 | Response to interferon-gamma | 6 | 6.59 | 3.58E-04 | 4.29E-02 |

| | | | | | |
|---|---|---|---|---|---|
| GO:0071216 | Cellular response to biotic stimulus | 7 | 6.54 | 1.20E-04 | 2.04E-02 |
| GO:0071222 | Cellular response to lipopolysaccharide | 6 | 6.49 | 3.89E-04 | 4.46E-02 |
| GO:0051091 | Positive regulation of DNA-binding transcription factor activity | 8 | 6.04 | 6.69E-05 | 1.56E-02 |
| GO:0032496 | Response to lipopolysaccharide | 9 | 5.75 | 3.34E-05 | 9.60E-03 |
| GO:0002237 | Response to molecule of bacterial origin | 9 | 5.5 | 4.65E-05 | 1.21E-02 |
| GO:0043312 | Neutrophil degranulation | 13 | 5.38 | 1.11E-06 | 2.52E-03 |
| GO:0002283 | Neutrophil activation involved in immune response | 13 | 5.34 | 1.22E-06 | 2.41E-03 |
| GO:0002446 | Neutrophil mediated immunity | 13 | 5.26 | 1.42E-06 | 2.25E-03 |
| GO:0110053 | Regulation of actin filament organization | 7 | 5.26 | 4.35E-04 | 4.74E-02 |
| GO:0042119 | Neutrophil activation | 13 | 5.24 | 1.49E-06 | 2.14E-03 |
| GO:0002274 | Myeloid leukocyte activation | 15 | 5.23 | 2.19E-07 | 1.73E-03 |
| GO:0036230 | Granulocyte activation | 13 | 5.19 | 1.65E-06 | 2.18E-03 |
| GO:0032970 | Regulation of actin filament-based process | 10 | 5.18 | 2.86E-05 | 8.70E-03 |
| GO:0043299 | Leukocyte degranulation | 13 | 5.15 | 1.80E-06 | 2.04E-03 |
| GO:0002444 | Myeloid leukocyte mediated immunity | 13 | 5.05 | 2.22E-06 | 2.20E-03 |
| GO:0002275 | Myeloid cell activation involved in immune response | 13 | 5.02 | 2.37E-06 | 2.08E-03 |
| GO:0006954 | Inflammatory response | 12 | 4.95 | 6.85E-06 | 4.51E-03 |
| GO:0050727 | Regulation of inflammatory response | 8 | 4.86 | 2.83E-04 | 3.70E-02 |
| GO:0032956 | Regulation of actin cytoskeleton organization | 8 | 4.66 | 3.72E-04 | 4.36E-02 |
| GO:1902903 | Regulation of supramolecular fiber organization | 8 | 4.51 | 4.65E-04 | 4.93E-02 |
| GO:2001233 | Regulation of apoptotic signaling pathway | 9 | 4.49 | 2.10E-04 | 2.91E-02 |
| GO:0002366 | Leukocyte activation involved in immune response | 13 | 4.23 | 1.42E-05 | 5.75E-03 |
| GO:0051090 | Regulation of DNA-binding transcription factor activity | 9 | 4.21 | 3.30E-04 | 3.98E-02 |
| GO:0002263 | Cell activation involved in immune response | 13 | 4.21 | 1.52E-05 | 5.99E-03 |
| GO:0045055 | Regulated exocytosis | 14 | 4.03 | 1.10E-05 | 5.28E-03 |
| GO:0002757 | Immune response-activating signal transduction | 11 | 4 | 1.11E-04 | 1.93E-02 |
| GO:0002443 | Leukocyte mediated immunity | 15 | 3.95 | 6.71E-06 | 4.61E-03 |
| GO:0009611 | Response to wounding | 11 | 3.91 | 1.36E-04 | 2.21E-02 |
| GO:0002253 | Activation of immune response | 12 | 3.88 | 7.12E-05 | 1.61E-02 |
| GO:0044089 | Positive regulation of cellular component biogenesis | 10 | 3.87 | 3.00E-04 | 3.82E-02 |
| GO:0071396 | Cellular response to lipid | 10 | 3.82 | 3.28E-04 | 4.02E-02 |
| GO:0002764 | Immune response-regulating signaling pathway | 11 | 3.75 | 1.94E-04 | 2.78E-02 |
| GO:0002252 | Immune effector process | 20 | 3.73 | 3.79E-07 | 1.50E-03 |
| GO:0051493 | Regulation of cytoskeleton organization | 10 | 3.72 | 4.02E-04 | 4.51E-02 |
| GO:0051347 | Positive regulation of transferase activity | 12 | 3.7 | 1.10E-04 | 1.94E-02 |

| | | | | | |
|---|---|---|---|---|---|
| GO:0006887 | Exocytosis | 14 | 3.58 | 4.02E-05 | 1.09E-02 |
| GO:0045321 | Leukocyte activation | 16 | 3.55 | 1.20E-05 | 5.57E-03 |
| GO:0006915 | Apoptotic process | 16 | 3.53 | 1.28E-05 | 5.62E-03 |
| GO:0001817 | Regulation of cytokine production | 12 | 3.5 | 1.81E-04 | 2.67E-02 |
| GO:0002682 | Regulation of immune system process | 28 | 3.44 | 5.88E-09 | 9.30E-05 |
| GO:0001775 | Cell activation | 17 | 3.24 | 1.93E-05 | 6.95E-03 |
| GO:0050776 | Regulation of immune response | 18 | 3.21 | 1.25E-05 | 5.64E-03 |
| GO:0045087 | Innate immune response | 12 | 3.19 | 4.17E-04 | 4.61E-02 |
| GO:1902533 | Positive regulation of intracellular signal transduction | 16 | 3.18 | 4.42E-05 | 1.16E-02 |
| GO:0030334 | Regulation of cell migration | 13 | 3.14 | 2.73E-04 | 3.60E-02 |
| GO:0000122 | Negative regulation of transcription by RNA polymerase II | 13 | 3.11 | 3.02E-04 | 3.82E-02 |
| GO:0051270 | Regulation of cellular component movement | 15 | 3.1 | 1.05E-04 | 1.92E-02 |
| GO:0051338 | Regulation of transferase activity | 15 | 3.09 | 1.08E-04 | 1.92E-02 |
| GO:0050778 | Positive regulation of immune response | 13 | 3.08 | 3.27E-04 | 4.04E-02 |
| GO:0012501 | Programmed cell death | 16 | 3.08 | 6.37E-05 | 1.53E-02 |
| GO:0033993 | Response to lipid | 13 | 3.03 | 3.82E-04 | 4.44E-02 |
| GO:0016477 | Cell migration | 14 | 2.99 | 2.56E-04 | 3.40E-02 |
| GO:0008219 | Cell death | 16 | 2.98 | 9.34E-05 | 1.87E-02 |
| GO:0034097 | Response to cytokine | 16 | 2.97 | 9.85E-05 | 1.85E-02 |
| GO:0033043 | Regulation of organelle organization | 19 | 2.97 | 2.02E-05 | 6.96E-03 |
| GO:0051707 | Response to other organism | 14 | 2.93 | 3.23E-04 | 4.02E-02 |
| GO:0043207 | Response to external biotic stimulus | 14 | 2.92 | 3.29E-04 | 4.01E-02 |
| GO:0051130 | Positive regulation of cellular component organization | 17 | 2.85 | 9.43E-05 | 1.84E-02 |
| GO:0009607 | Response to biotic stimulus | 14 | 2.84 | 4.29E-04 | 4.71E-02 |
| GO:0002684 | Positive regulation of immune system process | 16 | 2.83 | 1.68E-04 | 2.56E-02 |
| GO:0032940 | Secretion by cell | 14 | 2.83 | 4.47E-04 | 4.84E-02 |
| GO:0071345 | Cellular response to cytokine stimulus | 14 | 2.83 | 4.51E-04 | 4.85E-02 |
| GO:0044093 | Positive regulation of molecular function | 25 | 2.83 | 1.78E-06 | 2.16E-03 |
| GO:0031327 | Negative regulation of cellular biosynthetic process | 21 | 2.81 | 1.58E-05 | 6.11E-03 |
| GO:0009890 | Negative regulation of biosynthetic process | 21 | 2.76 | 2.00E-05 | 7.04E-03 |
| GO:0051253 | Negative regulation of RNA metabolic process | 18 | 2.75 | 9.06E-05 | 1.88E-02 |
| GO:0045892 | Negative regulation of transcription, DNA-templated | 16 | 2.75 | 2.37E-04 | 3.22E-02 |
| GO:0006952 | Defense response | 18 | 2.73 | 1.01E-04 | 1.85E-02 |
| GO:0045934 | Negative regulation of nucleobase-containing compound metabolic process | 19 | 2.7 | 7.06E-05 | 1.62E-02 |

| | | | | | |
|---|---|---|---|---|---|
| GO:0043085 | Positive regulation of catalytic activity | 19 | 2.68 | 7.97E-05 | 1.73E-02 |
| GO:1902531 | Regulation of intracellular signal transduction | 24 | 2.65 | 9.08E-06 | 4.79E-03 |
| GO:2000113 | Negative regulation of cellular macromolecule biosynthetic process | 18 | 2.64 | 1.48E-04 | 2.30E-02 |
| GO:0010558 | Negative regulation of macromolecule biosynthetic process | 19 | 2.63 | 1.00E-04 | 1.86E-02 |
| GO:1903507 | Negative regulation of nucleic acid-templated transcription | 16 | 2.63 | 3.86E-04 | 4.45E-02 |
| GO:1902679 | Negative regulation of RNA biosynthetic process | 16 | 2.62 | 3.93E-04 | 4.47E-02 |
| GO:0051240 | Positive regulation of multicellular organismal process | 22 | 2.56 | 3.91E-05 | 1.08E-02 |
| GO:0051128 | Regulation of cellular component organization | 31 | 2.54 | 7.11E-07 | 1.87E-03 |
| GO:0001932 | Regulation of protein phosphorylation | 18 | 2.54 | 2.45E-04 | 3.31E-02 |
| GO:0010941 | Regulation of cell death | 21 | 2.5 | 8.19E-05 | 1.75E-02 |
| GO:1901700 | Response to oxygen-containing compound | 19 | 2.5 | 1.95E-04 | 2.78E-02 |
| GO:0009967 | Positive regulation of signal transduction | 20 | 2.44 | 1.73E-04 | 2.58E-02 |
| GO:0002376 | Immune system process | 33 | 2.41 | 1.25E-06 | 2.20E-03 |
| GO:0006955 | Immune response | 22 | 2.41 | 9.50E-05 | 1.83E-02 |
| GO:0007166 | Cell surface receptor signaling pathway | 29 | 2.38 | 8.83E-06 | 4.81E-03 |
| GO:0010628 | Positive regulation of gene expression | 23 | 2.34 | 1.26E-04 | 2.10E-02 |
| GO:0045595 | Regulation of cell differentiation | 21 | 2.34 | 2.87E-04 | 3.69E-02 |
| GO:0048584 | Positive regulation of response to stimulus | 28 | 2.33 | 1.69E-05 | 6.38E-03 |
| GO:0031399 | Regulation of protein modification process | 21 | 2.32 | 3.06E-04 | 3.84E-02 |
| GO:0031324 | Negative regulation of cellular metabolic process | 29 | 2.31 | 1.28E-05 | 5.49E-03 |
| GO:0009605 | Response to external stimulus | 24 | 2.31 | 9.67E-05 | 1.84E-02 |
| GO:0051172 | Negative regulation of nitrogen compound metabolic process | 26 | 2.26 | 6.07E-05 | 1.52E-02 |
| GO:0051173 | Positive regulation of nitrogen compound metabolic process | 35 | 2.24 | 2.78E-06 | 2.31E-03 |
| GO:0065009 | Regulation of molecular function | 36 | 2.21 | 2.15E-06 | 2.27E-03 |
| GO:0031325 | Positive regulation of cellular metabolic process | 36 | 2.2 | 2.30E-06 | 2.14E-03 |
| GO:0050793 | Regulation of developmental process | 28 | 2.19 | 6.10E-05 | 1.51E-02 |
| GO:2000026 | Regulation of multicellular organismal development | 22 | 2.18 | 3.99E-04 | 4.51E-02 |
| GO:0050790 | Regulation of catalytic activity | 25 | 2.17 | 2.18E-04 | 3.00E-02 |
| GO:0010604 | Positive regulation of macromolecule metabolic process | 35 | 2.12 | 9.68E-06 | 4.94E-03 |
| GO:0009893 | Positive regulation of metabolic process | 37 | 2.07 | 6.23E-06 | 4.48E-03 |
| GO:0032879 | Regulation of localization | 28 | 2.07 | 1.84E-04 | 2.69E-02 |
| GO:0051239 | Regulation of multicellular organismal process | 32 | 2.07 | 4.17E-05 | 1.12E-02 |
| GO:0070887 | Cellular response to chemical stimulus | 29 | 2.05 | 1.37E-04 | 2.22E-02 |
| GO:0048583 | Regulation of response to stimulus | 45 | 2.05 | 4.18E-07 | 1.32E-03 |
| GO:0009892 | Negative regulation of metabolic process | 29 | 2.04 | 1.48E-04 | 2.32E-02 |

| | | | | | |
|---|---|---|---|---|---|
| GO:0009966 | Regulation of signal transduction | 32 | 1.95 | 1.26E-04 | 2.12E-02 |
| GO:0007165 | Signal transduction | 46 | 1.85 | 8.03E-06 | 4.54E-03 |
| GO:0023051 | Regulation of signaling | 34 | 1.85 | 2.47E-04 | 3.31E-02 |
| GO:0048518 | Positive regulation of biological process | 56 | 1.84 | 2.30E-07 | 1.21E-03 |
| GO:0048522 | Positive regulation of cellular process | 49 | 1.83 | 2.84E-06 | 2.24E-03 |
| GO:0010646 | Regulation of cell communication | 33 | 1.82 | 4.07E-04 | 4.53E-02 |
| GO:0023052 | Signaling | 48 | 1.81 | 7.48E-06 | 4.73E-03 |
| GO:0048731 | System development | 39 | 1.79 | 1.48E-04 | 2.33E-02 |
| GO:0007154 | Cell communication | 48 | 1.78 | 9.89E-06 | 4.89E-03 |
| GO:0048523 | Negative regulation of cellular process | 41 | 1.76 | 1.30E-04 | 2.13E-02 |
| GO:0048519 | Negative regulation of biological process | 46 | 1.76 | 2.30E-05 | 7.41E-03 |
| GO:0048856 | Anatomical structure development | 47 | 1.76 | 2.37E-05 | 7.50E-03 |
| GO:0032502 | Developmental process | 49 | 1.72 | 2.15E-05 | 7.25E-03 |
| GO:0007275 | Multicellular organism development | 42 | 1.69 | 1.99E-04 | 2.78E-02 |
| GO:0051716 | Cellular response to stimulus | 54 | 1.65 | 1.92E-05 | 7.07E-03 |
| GO:0050896 | Response to stimulus | 62 | 1.48 | 8.22E-05 | 1.73E-02 |
| **Molecular Function (MF)** | | | | | |
| GO:0035325 | Toll-like receptor binding | 3 | 49.99 | 5.22E-05 | 4.85E-02 |
| GO:0019901 | Protein kinase binding | 13 | 3.95 | 2.88E-05 | 4.47E-02 |
| GO:0019900 | Kinase binding | 14 | 3.78 | 2.22E-05 | 5.16E-02 |
| GO:0044877 | Protein-containing complex binding | 17 | 3.08 | 3.70E-05 | 4.31E-02 |
| GO:0005515 | Protein binding | 81 | 1.36 | 1.71E-05 | 7.94E-02 |
| **Cellular Component (CC)** | | | | | |
| GO:0002102 | Podosome | 4 | 27.58 | 2.12E-05 | 2.16E-02 |
| GO:0030667 | Secretory granule membrane | 10 | 6.64 | 3.51E-06 | 7.14E-03 |
| **PANTHER pathway** | | | | | |
| P00054 | Toll receptor signaling pathway | 6 | 18.75 | 1.33E-06 | 2.18E-04 |

**Table S6. The response to stimulus, immune system process, and cell death related Gene Ontology (GO) terms of biological process based on upregulated genes (>1.3-fold) in synaptosome**

| GO ID | Term annotated | Fold enrichment | p-value | FDR | Number of genes |
|---|---|---|---|---|---|
| **Response to stimulus related GO terms** | | | | | |
| GO:0050896 | Response to stimulus | 1.48 | 8.22E-05 | 1.73E-02 | 62 |
| GO:0006952 | ➢ defense response | 6.66 | 1.12E-04 | 2.11E-02 | 18 |
| GO:0006954 | - inflammatory response | 2.48 | 8.37E-06 | 4.16E-03 | 12 |
| GO:0002523 | - leukocyte migration involved in inflammatory response | 46.15 | 6.40E-05 | 1.51E-02 | 3 |
| GO:0002269 | - leukocyte activation involved in inflammatory response | 2.48 | 7.95E-06 | 4.84E-03 | 5 |
| GO:0050727 | - regulation of inflammatory response | 4.86 | 2.83E-04 | 3.70E-02 | 8 |
| GO:0150076 | - neuroinflammatory response | 24.24 | 3.37E-05 | 9.51E-03 | 4 |
| GO:0061900 | - glial cell activation | 25.8 | 2.69E-05 | 8.35E-03 | 4 |
| GO:0001774 | - microglial cell activation | 36.36 | 7.95E-06 | 4.66E-03 | 4 |
| GO:1903978 | - regulation of microglial cell activation | 29.99 | 1.97E-04 | 2.78E-02 | 3 |
| GO:0098542 | - defense response to other organism | 4.7 | 2.70E-04 | 3.58E-02 | 14 |
| GO:0045087 | - innate immune response | 3.19 | 4.17E-04 | 4.61E-02 | 12 |
| GO:0034341 | - response to interferon-gamma | 6.59 | 3.58E-04 | 4.29E-02 | 6 |
| GO:0009607 | ➢ response to biotic stimulus | 2.84 | 4.29E-04 | 4.71E-02 | 14 |
| GO:0043207 | - response to external biotic stimulus | 2.92 | 3.29E-04 | 4.01E-02 | 14 |
| GO:0009605 | ➢ response to external stimulus | 2.31 | 9.67E-05 | 1.84E-02 | 24 |
| GO:1901700 | ➢ response to oxygen-containing compound | 7.87 | 3.00E-04 | 3.88E-02 | 19 |
| **Immune system process related GO terms** | | | | | |
| GO:0002376 | Immune system process | 2.41 | 1.25E-06 | 2.20E-03 | 33 |
| GO:0002252 | ➢ immune effector process | 3.73 | 3.79E-07 | 1.50E-03 | 20 |
| GO:0002443 | - leukocyte mediated immunity | 3.95 | 6.71E-06 | 4.61E-03 | 15 |
| GO:0002444 | - myeloid leukocyte mediated immunity | 5.34 | 1.22E-06 | 2.41E-03 | 13 |
| GO:0002446 | - neutrophil mediated immunity | 5.26 | 1.42E-06 | 2.25E-03 | 13 |
| GO:0006955 | ➢ immune response | 2.41 | 9.50E-05 | 1.83E-02 | 22 |
| GO:0002263 | - cell activation involved in immune response | 4.21 | 1.52E-05 | 5.99E-03 | 13 |
| GO:0002366 | - leukocyte activation involved in immune response | 4.23 | 1.42E-05 | 5.75E-03 | 13 |
| GO:0002275 | - myeloid cell activation involved in immune response | 5.02 | 2.37E-06 | 2.08E-03 | 13 |

| | | | | | |
|---|---|---|---|---|---|
| GO:0002283 | - neutrophil activation involved in immune response | 5.34 | 1.22E-06 | 2.41E-03 | 13 |
| GO:0050776 | - regulation of immune response | 3.21 | 1.25E-05 | 5.64E-03 | 18 |
| GO:0002764 | - immune response-regulating signaling pathway | 3.75 | 1.94E-04 | 2.78E-02 | 11 |
| GO:0050778 | - positive regulation of immune response | 3.08 | 3.27E-04 | 4.04E-02 | 13 |
| GO:0002253 | ➢ activation of immune response | 3.88 | 7.12E-05 | 1.61E-02 | 12 |
| GO:0002757 | - immune response-activating signal transduction | 4 | 1.11E-04 | 1.93E-02 | 11 |
| GO:0002682 | ➢ regulation of immune system process | 3.44 | 5.88E-09 | 9.30E-05 | 28 |
| GO:0002684 | ➢ positive regulation of immune system process | 2.83 | 1.68E-04 | 2.56E-02 | 16 |
| **Cell death related GO terms** | | | | | |
| GO:0008219 | Cell death | 2.98 | 9.34E-05 | 1.87E-02 | 16 |
| GO:0010941 | ➢ regulation of cell death | 2.5 | 8.19E-05 | 1.75E-02 | 21 |
| GO:0012501 | ➢ programmed cell death | 3.08 | 6.37E-05 | 1.53E-02 | 16 |
| GO:0006915 | - apoptotic process | 4.52 | 4.90E-05 | 1.26E-02 | 15 |
| GO:0110089 | - regulation of hippocampal neuron apoptotic process | 99.98 | 3.63E-04 | 4.29E-02 | 2 |
| GO:2001233 | - regulation of apoptotic signaling pathway | 4.49 | 2.10E-04 | 2.91E-02 | 9 |

**Table S7. Correlation between gene expressions and confounding variables in the total fraction**

| Variables | RASD1 | H1FX | SYN1 | IGFBP4 | SYN2 | HPSD1 | RBBP4 | ZFN32 |
|---|---|---|---|---|---|---|---|---|
| Age | r=0.049<br>*p*=0.86 | r=0.16<br>*p*=0.57 | r=0.39<br>*p*=0.15 | r=-0.04<br>*p*=0.87 | r=0.18<br>*p*=0.52 | r=-0.02<br>*p*=0.92 | r=0.12<br>*p*=0.65 | r=-0.17<br>*p*=0.54 |
| PMI | r=0.01<br>*p*=0.97 | r=-0.08<br>*p*=0.77 | r=-0.14<br>*p*=0.61 | r=0.12<br>*p*=0.67 | r=0.21<br>*p*=0.44 | r=0.24<br>*p*=0.39 | r=-0.003<br>*p*=0.99 | r=-0.22<br>*p*=0.41 |
| Brain pH | r=-0.25<br>*p*=0.39 | r=0.13<br>*p*=0.64 | r=0.14<br>*p*=0.62 | r=-0.19<br>*p*=0.49 | r=0.07<br>*p*=0.79 | r=-0.49<br>*p*=0.06 | r=-0.44<br>*p*=0.10 | r=-0.17<br>*p*=0.55 |
| Sex | t=1.3<br>*p*=0.19 | t=0.83<br>*p*=0.42 | t=1.2<br>*p*=0.24 | t=1.4<br>*p*=0.17 | t=0.91<br>*p*=0.38 | t=1.4<br>*p*=0.17 | t=1.1<br>*p*=0.27 | t=0.20<br>*p*=0.84 |
| Alcohol Abuse | t=0.26<br>*p*=0.79 | t=1.41<br>*p*=0.18 | t=0.68<br>*p*=0.51 | t=1.44<br>*p*=0.17 | t=1.26<br>*p*=0.23 | t=1.07<br>*p*=0.30 | t=4.38<br>*p*=0.001 | t=2.25<br>*p*=0.04 |
| Antidepressant | t=1.2<br>*p*=0.22 | t=0.63<br>*p*=0.53 | t=0.006<br>*p*=0.99 | t=0.32<br>*p*=0.75 | t=0.13<br>*p*=0.89 | t=0.72<br>*p*=0.48 | t=0.07<br>*p*=0.95 | t=0.12<br>*p*=0.91 |

Values denote p-value. Ct: control, MDD: Major Depressive Disorder, dlPFC: Dorsolateral prefrontal cortex, PMI: postmortem interval, miR: microRNA. The asterisk indicates a statistically significant correlation ($p < 0.05$).

**Table S8. Correlation between gene expressions and confounding variables in the synaptic fraction**

| Variables | TLR2 | ELK1 | ZFP36 | IRF1 | HES5 | ASPDH | CYB561D1 | FUT9 |
|---|---|---|---|---|---|---|---|---|
| Age | r=-0.06<br>*p*=0.85 | r=0.18<br>*p*=0.55 | r=0.30<br>*p*=0.34 | r=0.12<br>*p*=0.72 | r=0.14<br>*p*=0.63 | r=-0.01<br>*p*=0.96 | r=-0.01<br>*p*=0.96 | r=0.001<br>*p*=0.99 |
| PMI | r=-0.32<br>*p*=0.29 | r=-0.001<br>*p*=1 | r=-0.30<br>*p*=0.34 | r=0.05<br>*p*=0.87 | r=0.20<br>*p*=0.47 | r=-0.23<br>*p*=0.41 | r=0.23<br>*p*=0.41 | r=0.28<br>*p*=0.32 |
| Brain pH | r=0.09<br>*p*=0.77 | r=0.24<br>*p*=0.43 | r=-0.24<br>*p*=0.45 | r=-0.19<br>*p*=0.56 | r=-0.18<br>*p*=0.52 | r=-0.07<br>*p*=0.81 | r=0.15<br>*p*=0.60 | r=-0.19<br>*p*=0.51 |
| Sex | t=0.98<br>*p*=0.34 | t=1.2<br>*p*=0.25 | t=0.87<br>*p*=0.40 | t=0.77<br>*p*=0.45 | t=0.92<br>*p*=0.37 | t=1.6<br>*p*=0.13 | t=0.50<br>*p*=0.62 | t=1.1<br>*p*=0.26 |
| Antidepressant | t=0.35<br>*p*=0.73 | t=0.63<br>*p*=0.54 | t=0.13<br>*p*=0.89 | t=1.4<br>*p*=0.19 | t=0.61<br>*p*=0.55 | t=0.52<br>*p*=0.61 | t=0.16<br>*p*=0.87 | t=1.5<br>*p*=0.14 |
| Alcohol Abuse | t=0.99<br>*p*=0.34 | t=1.05<br>*p*=0.31 | t=0.79<br>*p*=0.69 | t=1.10<br>*p*=0.29 | t=1.98<br>*p*=0.07 | t=.35<br>*p*=0.73 | t=0.2.19<br>*p*=0.05 | t=1.59<br>*p*=0.13 |

Values denote p-value. Ct: control, MDD: Major Depressive Disorder. PMI: postmortem interval. The asterisk indicates a statistically significant correlation (p <0.05).

**Table S9. Synaptic/total fraction ratio of genes in control subjects (top and bottom 20)**

| Gene symbol | Ensembl ID | Synaptosome/total fraction ratio | Chromosomal location | Strand |
|---|---|---|---|---|
| **The ratio of enrichment genes top 20** | | | | |
| EPDR1 | ENSG00000086289.11_3 | 8.869108 | chr7:37723446-37991543 | + |
| AC025594.2 | ENSG00000272899.3_4 | 7.371736 | chr7:128506362-128512098 | + |
| PMP2 | ENSG00000147588.6_2 | 7.325013 | chr8:82352561-82359758 | - |
| LYNX1 | ENSG00000180155.19_2 | 7.18706 | chr8:143852615-143859226 | - |
| GIPC1 | ENSG00000123159.15_3 | 6.425831 | chr19:14588572-14606961 | - |
| E2F1 | ENSG00000101412.12_2 | 4.523676 | chr20:32263489-32274210 | - |
| AC091959.3 | ENSG00000275740.1_3 | 4.492684 | chr5:145583113-145718814 | + |
| EBF4 | ENSG00000088881.20_3 | 3.781456 | chr20:2673524-2740754 | + |
| GJC2 | ENSG00000198835.3_2 | 3.732905 | chr1:228337553-228347527 | + |
| TMEM189-UBE2V1 | ENSG00000124208.16_3 | 3.572151 | chr20:48697661-48770174 | - |
| C1QA | ENSG00000173372.16_3 | 3.543936 | chr1:22962999-22966101 | + |
| FIP1L1 | ENSG00000145216.15_3 | 3.511716 | chr4:54243810-54327028 | + |
| CAVIN1 | ENSG00000177469.12_3 | 3.456352 | chr17:40554470-40575535 | - |
| DZIP1 | ENSG00000134874.17_2 | 3.447099 | chr13:96230456-96296957 | - |
| FLII | ENSG00000177731.15_2 | 3.377484 | chr17:18148150-18162230 | - |
| OGDH | ENSG00000105953.14_2 | 3.082193 | chr7:44646171-44748665 | + |
| DYSF | ENSG00000135636.13_2 | 2.67007 | chr2:71680852-71913898 | + |
| POLR2J2 | ENSG00000267645.5_3 | 2.619659 | chr7:102277496-102312088 | - |
| GINM1 | ENSG00000055211.12_2 | 2.53371 | chr6:149887430-149912884 | + |
| INO80B-WBP1 | ENSG00000274049.4_3 | 2.489328 | chr2:74682215-74688011 | + |
| **The ratio of depleted genes bottom 20** | | | | |
| C1orf64 | ENSG00000183888.4_2 | 0.251085 | chr1:16330731-16335302 | + |
| GIMAP7 | ENSG00000179144.4_2 | 0.249776 | chr7:150211918-150218161 | + |
| ACVRL1 | ENSG00000139567.12_2 | 0.248565 | chr12:52300692-52317145 | + |
| GIT2 | ENSG00000139436.20_3 | 0.243924 | chr12:110367597-110434194 | - |
| FLT1 | ENSG00000102755.11_2 | 0.241182 | chr13:28874481-29069282 | - |
| ABCG2 | ENSG00000118777.10_3 | 0.232325 | chr4:89011416-89152474 | - |
| FGR | ENSG00000000938.12_2 | 0.224675 | chr1:27938575-27961788 | - |
| CP | ENSG00000047457.13_2 | 0.224644 | chr3:148880197-148939842 | - |

| | | | | |
|---|---|---|---|---|
| TM4SF1 | ENSG00000169908.11_3 | 0.222281 | chr3:149086809-149095652 | - |
| ABCB1 | ENSG00000085563.14_2 | 0.218971 | chr7:87132949-87342639 | - |
| CDH5 | ENSG00000179776.17_3 | 0.218477 | chr16:66400525-66438689 | + |
| CXCL1 | ENSG00000163739.4_2 | 0.205596 | chr4:74735110-74736959 | + |
| EDN1 | ENSG00000078401.6_2 | 0.199844 | chr6:12290596-12297427 | + |
| POLR2J2 | ENSG00000228049.7_3 | 0.195706 | chr7:102305815-102312076 | - |
| AC004922.1 | ENSG00000284292.1_2 | 0.192021 | chr7:98923502-98992276 | + |
| LYNX1 | ENSG00000284505.1_1 | 0.188766 | chr8:143845752-143859640 | - |
| GINS1 | ENSG00000101003.9_2 | 0.182566 | chr20:25388363-25433264 | + |
| HIGD1B | ENSG00000131097.6_2 | 0.179365 | chr17:42923721-42927848 | + |
| CSF3 | ENSG00000108342.12_2 | 0.172712 | chr17:38171614-38174066 | + |
| C7orf55-LUC7L2 | ENSG00000269955.2_3 | 0.137439 | chr7:139026106-139107345 | + |

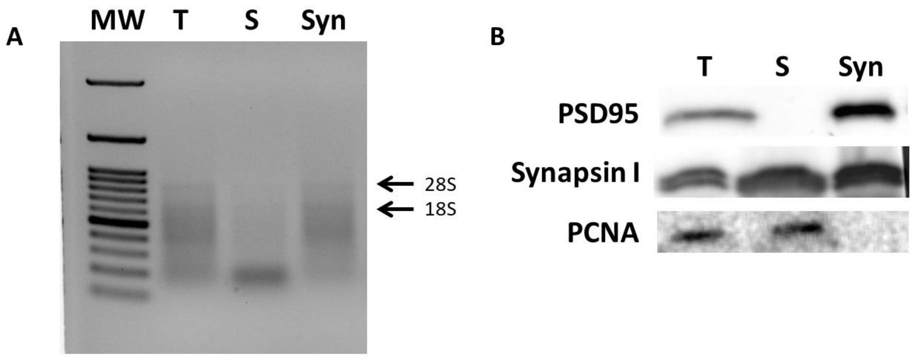


**Figure S1. Verification of RNA quality with gel electrophoresis and validation of synaptosome with Western Blot.** The result of western blot about validation tests and gel electrophoresis about isolated RNA. (A) An equal amount of RNAs (500 ng) was loaded in 0.8% TAE Agarose gel and stained with Ethidium Bromide. MW is 100bp plus ladder size marker. (B) An equal amount of proteins (20 ug) were loaded to subject to PAGE and blotted. MW, molecular weight; T, total fraction; S, S fraction; Syn, synaptosome.

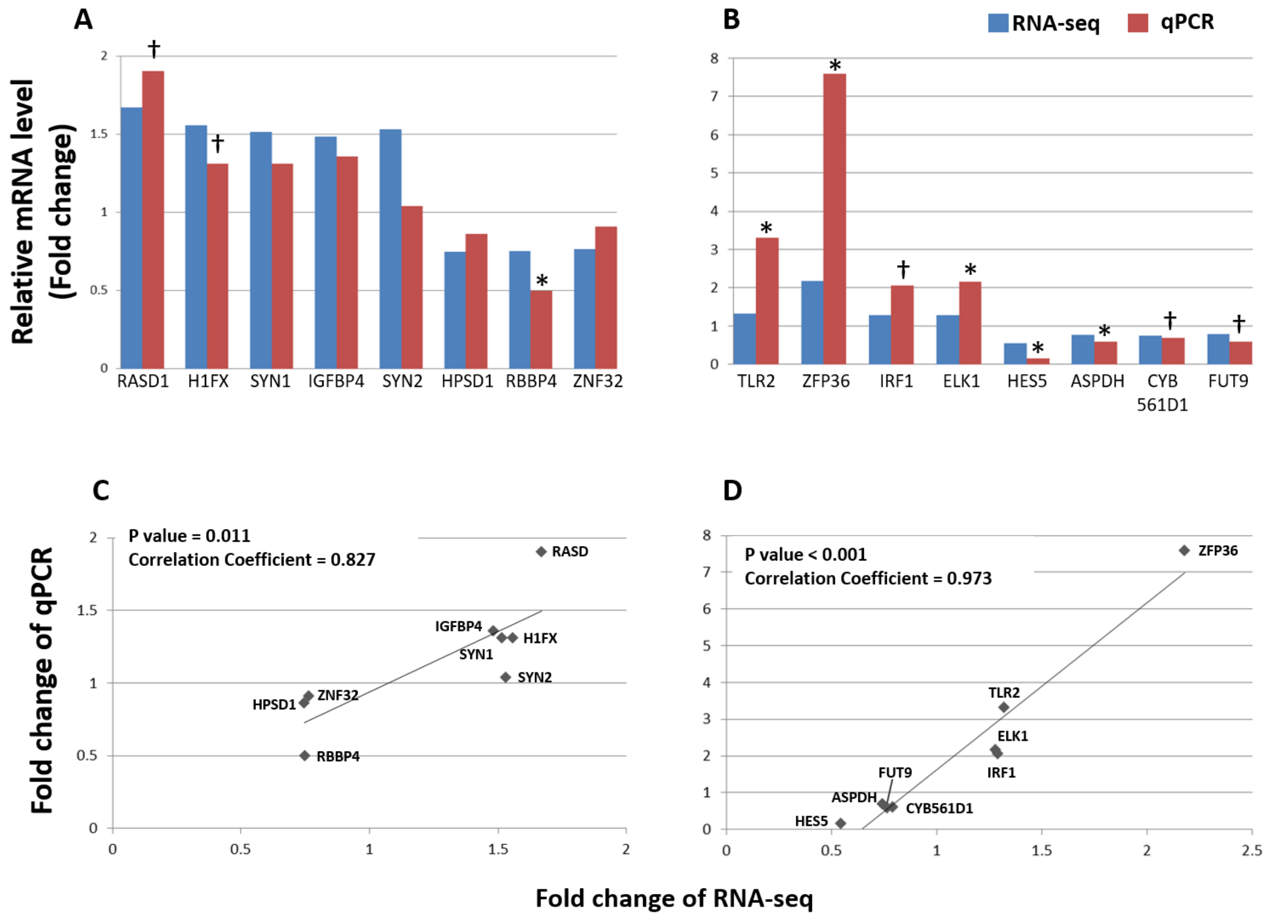


**Figure S2. The fold change comparison based on relative quantification of gene expression and scatter plot of correlation between fold change of RNA-seq and qPCR**. A) The gene expression differences measured by qPCR of total fraction are as follows: RASD1: t = -1.788, df = 17.5, p = 0.091; H1FX: t = -1.948, df = 28, p = 0.061; SYN1: t = -0.982, df = 28, p = 0.335; IGFBP4: t = -1.598, df = 21.3, p = 0.125; SYN2: t = -0.128, df = 28, p = 0.899; HPSD1: t = 0.801, df = 28, p = 0.430; RBBP4: t = 2.433, df = 16.3, p = 0.027; ZNF32: t = 0.787, df = 28, p = 0.438. B) The gene expression differences measured by qPCR of synaptic fraction are as follows: TLR2: t = -2.592, df = 12.5, p = 0.023; ZFP36: t = -2.411, df = 13.1, p = 0.031; IRF1: t = -1.923, df = 14.5, p = 0.074; ELK1:t = -3.220, df = 19.3, p = 0.004; HES5: t = 2.795, df = 14.2, p = 0.014; ASPDH: t = 2.593, df = 28, p = 0.015; CYB561D1: t = 1.754, df = 28, p = 0.090; FUT9: t = 1.825, df = 28, p = 0.079. Values denote average ± SEM. ‘a’ denotes the statistical significance between control and MDD subjects. (n=15 in control, n=15 in MDD; outliers excluded). The geometric mean (GAPDH, ACTB, and 18s rRNA) was used as normalizer. * $p < 0.05$, † $p < 0.01$. The scatter plot of correlation between fold change of qPCR and RNA-seq in total fraction (C) and synaptic fraction (D). X-axis indicates the fold change of RNA-seq, while Y-axis shows that of qPCR data (MDD/Ct). MDD, Major Depressive Disorder

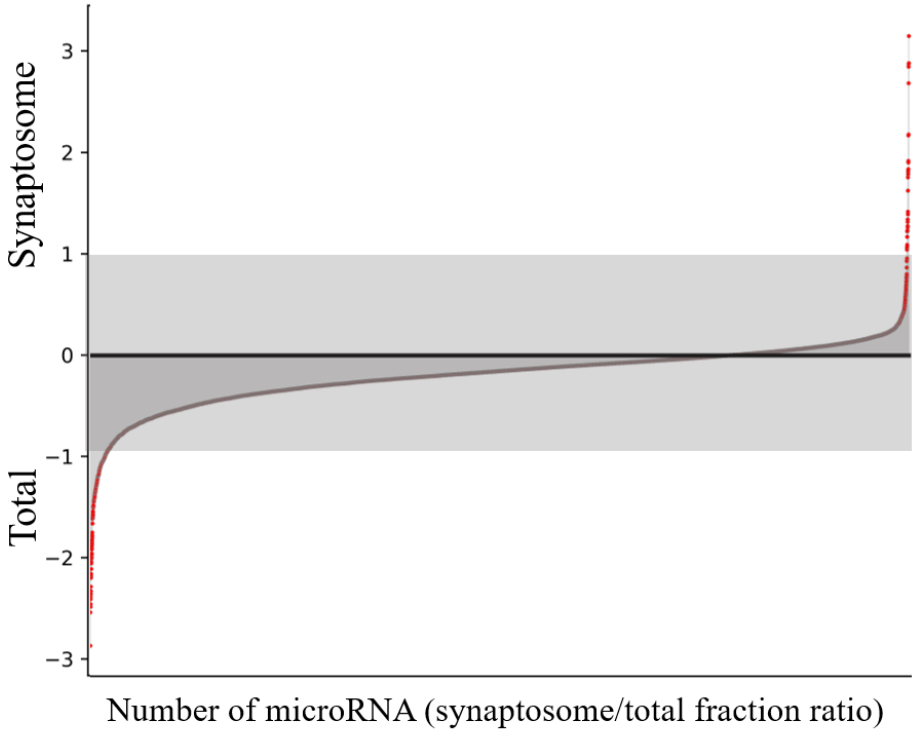


**Figure S3. Enrichment ratios of synaptic fraction gene expression compared to total fraction**. Gene expressions ratio of synaptic/total fraction about control subjects as measured by RNA-seq. The synaptic/total ratio are shown as log(base=2) transformed values.

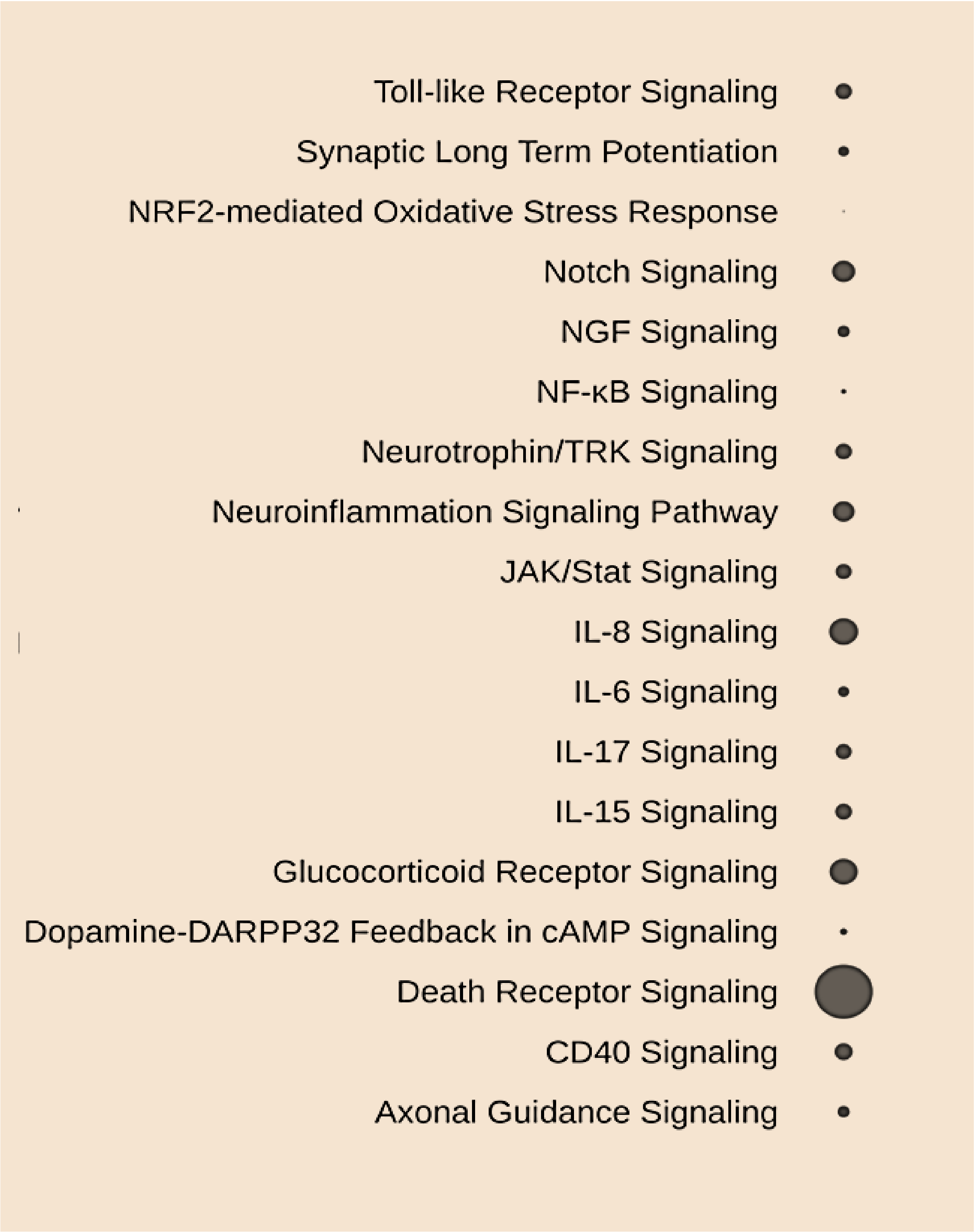


**Figure S4. The result of canonical pathway in significantly altered shift genes**. The analysis was performed by 119 shifted ratio genes (84 up and 35 down together). Spots/circles size is function of –log(base=10) of fisher exact test enrichment p-value.
